# The spectrum of health conditions in community-based cross-sectional surveys in Southeast Asia 2010-21: a scoping review

**DOI:** 10.1186/s12889-024-19347-3

**Published:** 2024-07-11

**Authors:** Meiwen Zhang, Hannah Kozlowski, Rusheng Chew, Nan Shwe Nwe Htun, Shaun K. Morris, Carolyn Akladious, Abdur Razzaque Sarker, Yoel Lubell, Thomas J. Peto

**Affiliations:** 1 Mahidol-Oxford Tropical Medicine Research Unit, Faculty of Tropical Medicine, Mahidol University, Bangkok, Thailand; 2 Centre for Tropical Medicine and Global Health, Nuffield Department of Medicine, University of Oxford, Oxford, UK; 3 University of Toronto Temerty Faculty of Medicine, Toronto, Canada; 4 Division of Infectious Diseases and Centre for Global Child Health, Hospital for Sick Children, Toronto, ON Canada; 5 Department of Paediatrics, University of Toronto, Toronto, Canada; 6 Faculty of Medicine, University of Queensland, Brisbane, Australia; 7 Bangladesh Institute of Development Studies (BIDS), Dhakar, Bangladesh

**Keywords:** Southeast Asia, Cross-sectional surveys, Community-based survey, Epidemiology

## Abstract

**Background:**

Southeast Asia is undergoing an epidemiological transition with non-communicable illnesses becoming increasingly important, yet infectious diseases (tuberculosis, HIV, hepatitis B, malaria) remain widely prevalent in some populations, while emerging and zoonotic diseases threaten. There are also limited population-level estimates of many important heath conditions. This restricts evidence-based decision-making for disease control and prevention priorities. Cross-sectional surveys can be efficient epidemiological tools to measure the prevalence of a wide range of diseases, but no systematic assessment of their coverage of different health conditions has been produced for the region.

**Methods:**

We conducted a systematic search in Medline, Embase, Global Health, CINAHL, Scopus, Web of Science Core Collection, and Global Index Medicus, and additionally Google Scholar. Our inclusion criteria were cross-sectional surveys conducted with community-based recruitment, in Bangladesh, Cambodia, Laos, Myanmar, and Thailand, published between January 1, 2010 and January 27, 2021, and reporting the prevalence of any health condition.

**Results:**

542 publications from 337 surveys were included. Non-communicable conditions (*n* = 205) were reported by more surveys than infectious conditions (*n* = 124). Disability (*n* = 49), self-report history of any disease or symptoms (*n* = 35), and self-perceived health status (*n* = 34), which reflect a holistic picture of health, were studied by many fewer surveys. In addition, 45 surveys studied symptomatic conditions which overlap between non-communicable and infectious conditions. The most surveyed conditions were undernutrition, obesity, hypertension, diabetes, intestinal parasites, malaria, anemia, diarrhea, fever, and acute respiratory infections. These conditions overlap with the most important causes of death and disability in the Global Burden of Disease study. However, other high-burden conditions (e.g. hearing loss, headache disorder, low back pain, chronic liver and kidney diseases, and cancers) were rarely studied.

**Conclusion:**

There were relatively few recent surveys from which to estimate representative prevalences and trends of health conditions beyond those known to be high burden. Expanding the spectrum of health conditions in cross-sectional surveys could improve understanding of evolving disease patterns in the region.

**Supplementary Information:**

The online version contains supplementary material available at 10.1186/s12889-024-19347-3.

## Background

South and Southeast Asia encompass a geographical area comprising 15 countries with diverse geography, unique history, and distinct social and economic features. These factors have contributed to variations and similarities in health status and healthcare systems across these countries [1]. Bangladesh, Cambodia, Myanmar, Laos, and Thailand, located centrally within the region, represent a mix of Lower-Middle-Income Countries and an Upper-Middle-Income Country. The demographic and economic makeup of these countries is changing rapidly, and the current disease epidemiological data do not reflect these changes. As the traditional leading causes of mortality, communicable diseases (e.g. malaria, tuberculosis, and diarrheal diseases) and maternal and child health, have been successfully addressed by vertical programs in many parts of the region [1–8]. The increasing burden of non-communicable diseases (e.g. diabetes, cardiovascular disease, cancer), and emerging infectious diseases (e.g., chikungunya, or dengue) have put new parts of the population at risk, while historically at-risk parts of the population remain vulnerable to other neglected tropical diseases [9–13].

Although the existing data collection mechanism capture some of the changes, there are major gaps for certain diseases and populations. The data on traditionally high-burden diseases are updated by monitoring and evaluation systems within the corresponding vertical programs [2–6]. Prevalence of non-communicable disease, such as cardiovascular disease and diabetes, are reported by regularly conducted population-based national surveys (e.g., Demographic and Health Survey (DHS), WHO’s STEPwise approach to NCD risk factor surveillance) [14, 15]. However, there is scattered data on other diseases. Information on a wide range of diseases and populations can be obtained from existing surveillance or registration systems (e.g. health information systems, disease-specific registration systems, and death registration systems), but the coverage of these systems is limited [16, 17]. Regularly updated population-level disease burden estimates, such as the Global Burden of Disease Study (GBD) also exist, but the lack of primary data sources impedes the accuracy of the modeling results [18, 19]. The incomplete knowledge of current epidemiology in the region constrains decision makers’ ability to make evidence-based decisions- to identify health priorities, allocate resources, set targets, and monitor progress at a health system level.

In this context, cross-sectional surveys are an effective research method to provide population-level prevalence estimates. These surveys can capture a wide range of health-related factors within a relatively short implementation period and at a relatively low cost compared to other population-based data sources. No systematic assessment of the coverage of different health conditions by cross-sectional surveys has been produced for the region. To inform the design of future prevalence surveys, we conducted a scoping review with three key aims. Firstly, to understand where and when community based cross-sectional surveys have recently been conducted within the region. Secondly, to identify the health conditions studied in these surveys including variations across geographies and populations. Thirdly, to identify knowledge gaps that may serve as areas of focus in future surveys.

## Methods

This study was conducted according to the Preferred Reporting Items for Systematic Review and Meta-analysis Extension for Scoping Review (PRISMA-ScR) and the JBI Manual for Evidence Synthesis [20, 21]. The study protocol is registered with the Open Science Framework (OSF registration 10.17605/OSF.IO/9GBNK).

### Literature search strategy

A systematic search was conducted in seven databases: Medline, Embase, Global Health, CINAHL, Scopus, Web of Science Core Collection, and Global Index Medicus. Additionally, we searched Google Scholar for grey literature. We employed a broad search string that consisted of Medical Subject Headings (MeSH) and free-text terms, and excluded publications published before 2010. The full search terms are included in Appendix S1.

### Study selection and full-text review

**Fig. 1:**
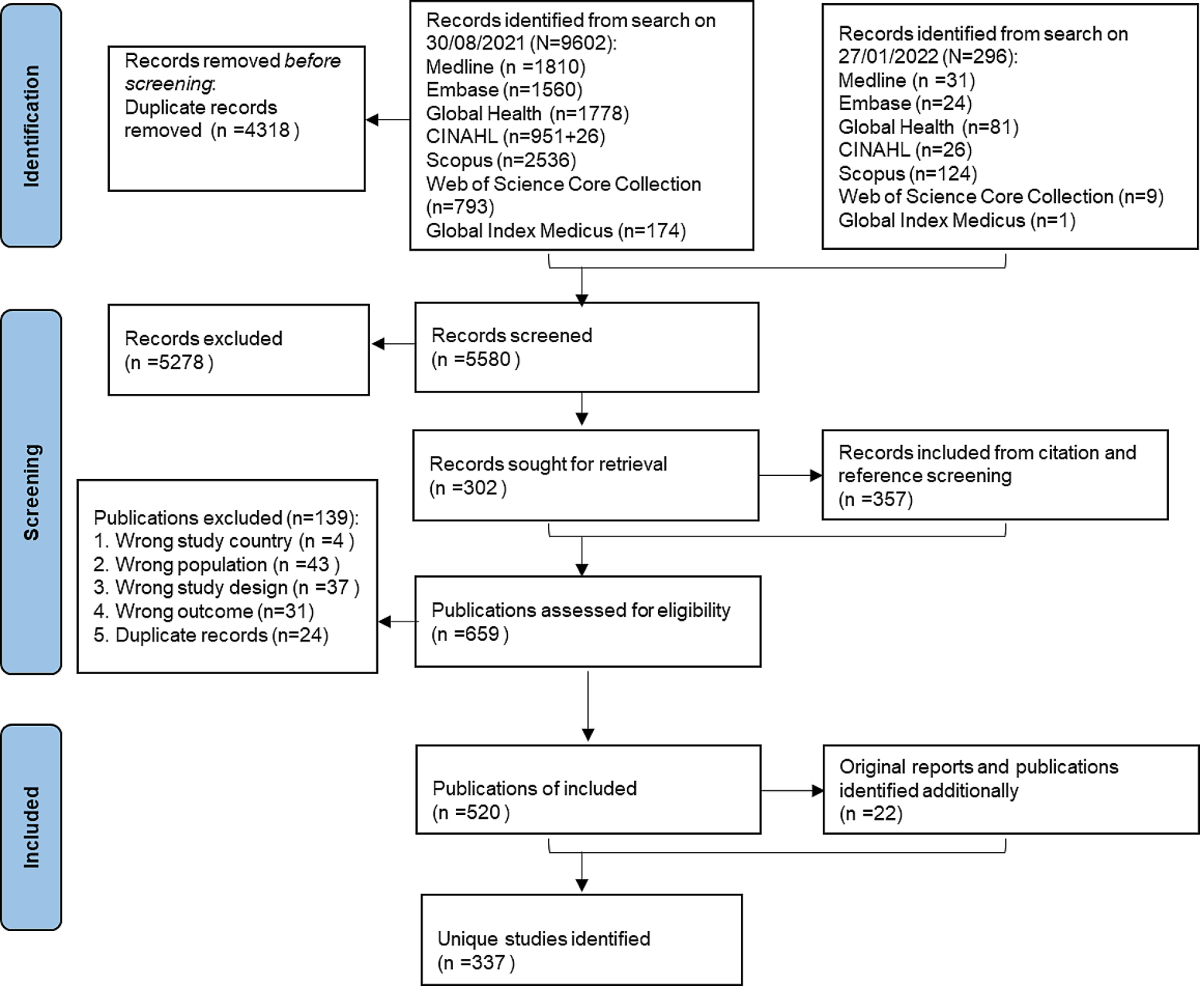
PRISMA flowchart showing selection of studies

The screening process followed JBI recommendations, and the details are in Appendix S2. Only publications that meet the predefined inclusion criteria: studies conducted in Bangladesh, Cambodia, Laos, Myanmar, and Thailand; published between January 1, 2010, and January 27, 2022; and cross-sectional health surveys with participants recruited from the community, irrespective of symptoms or disease status; reported the number of participants with a condition and the total number of participants assessed. The PRISMA flowchart for the review is in Fig. 1.

### Data extraction and analysis

Data from publications deemed eligible for the review were extracted into an Excel sheet with predefined variables, including bibliographic metrics, study methods, and health conditions reported as prevalence, along with the age and sex of the study population.

The original survey from which the data was primarily collected for each publication was identified, and all publications were grouped by “survey” (e.g. multiple publications used data collected from a particular DHS survey in Bangladesh). The identification of original data source of a publication is detailed in Appendix S2.

### Definitions and categorization

The urbanicity coverage of the studies was recorded as it was described in the publications, as urban, rural, both urban and rural, or nationally representative. If a study did not describe the coverage, it was recorded as “unspecified.”

The age distribution of the study population for each condition was organized into a context appropriate classification. The age groups were categorized as: Lifespan, Childhood, and Adulthood. Child and Adulthood category contains subcategories of age groups that with narrower age bands (Table 1). The exact match of inclusion of age group of a survey and the corresponding age group applied in this review is detailed in Appendix S3.

**Table 1:** Categorizations of age groups and health conditions

Age groups (years)	Subcategories (years)
Lifespan	When a condition was studied among participants recruited without specific age criteria or across both the “Childhood” and “Adulthood” groups
Childhood (≤ 19):	− Child (≤ 19) − Preschool child(≤ 5) − School-age child (5–19)
Adulthood (≥ 15)	− Adulthood (≥ 15) − Reproductive age (15–49) − Reproductive age and older adulthood (15–64) − Older adulthood and retirement age (≥ 50)
**Health condition categories**	**Categorization and definition**
1. Non-communicable conditions	Non-communicable conditions reflecting the same underlying pathophysiology but representing different degrees of severity were grouped together(e.g. elevated blood glucose level, prediabetes, and diabetes were grouped as one type of health condition and referred to as “diabetes”).
2. Infectious	Infectious conditions were categorized based on a. Direct microbiologic identification of a pathogen (e.g., through culture or molecular tests) or b. Indirect measures (e.g., serologic tests). c. Infective syndromes without a reported microbiologic cause, such as acute respiratory infection (ARI), were also categorized as infectious conditions. Based on the microbiologic results or lack thereof the subcategories applied: Bacterial, viral, parasitic, non-specified
3. Symptoms and abnormal findings (Symptoms)	Conditions that could not be determined as infectious or non-infectious causes (e.g., fever, diarrhea).
4. Disability and functional limitation (Disability)	Disability was defined either directly by the study that referring a condition as disability, or categorized by the review authors when a condition fit the WHO definition of disability ^22^. Based on the aspects of disability, the conditions were grouped into: a. Body structure: impairments of anatomical parts of the body such as organs, limbs and their components b. Body function: impairments of physiological functions of body systems (including psychological functions) c. Activity and participation (Activity): difficulties and problems of execution of a task or action, and/ or involvement in a life situation ^23^. d. Activity of daily living: Fundamental skills, related with body function and activity, required to independently care for oneself ^24,25^ .
1. Self-perceived health status	Self-perceived health status encompassed the questionnaire-based assessments of a. Self-rated health: a single question assessing an individual’s own opinion on their health or b. health -related quality of life: the impact of health status on quality of life ^26–29^.
2. General health condition inquiries (General health)	It describes the data collected from the questions asked to the survey participants, soliciting self-reported presence or history without limiting the answers to specific diseases or symptoms. E.g. Have you been ill in the past 30 days? Could you tell me all the symptoms or diagnosis you had?

Health conditions, symptoms, laboratory or point-of-care test results, and physical examination results were recorded as reported in the publications. They were then placed into mutually exclusive categories, based primarily on the International Classification of Primary Care 3rd Edition (ICPC-3), including non-communicable conditions, infectious conditions, symptoms, disability and functional limitation (disability), self-perceived health status, general health condition inquires (general health) [22–30] (Table 1). The details of the categorization are described in Appendix S3.

### Comparison to global burden of disease estimates

To understand how the conditions studied by prevalence surveys compared to the highest burden conditions as determined by the Global Burden of Disease project, we:

1. Extracted the conditions that ranked the top 20 causes of death and disability (Causes) with highest disability-adjusted-life-years (DALY) rate (/100,000 population) of each country [31].
2. Summed the number of times a cause was studied in all included surveys of each country.
3. Identified country-specific leading disease burdens that were studied by a high number, a low number, or by no surveys.

To understand how well these causes were studied or described by other types of studies or data sources not included in our review, we:

1. Extracted the metadata of GDB of the top high-burden causes of each country, and summed the number of references of a cause which the data collection completed from 2010 onwards [32].
2. We assumed the number indicated the availability of a wider range of data source that consistent with the inclusion criteria of data source used in GBD (e.g. vital registration system, reports), and identify causes with high number, and low number, or no available data from data sources, beyond the surveys that are included in the review.

### Statistical analysis

The unit of analysis was the “survey,” which represented the original data source of multiple publications. Each study was characterized by the study country, rural or urban coverage, and whether it was a repeated survey. Each health condition was further described by the target population’s age group. All analyses were carried out using R software version 4.2.1 (R Foundation for Statistical Computing, Vienna, Austria), and graphical presentations were done using the ggplot2 library.

## Results

The screening process and publication inclusion are shown in Fig. 1. A total of 542 publications were finally included, reporting results from 337 surveys (Supplementary material: Summary of publications and surveys, and conditions studied).

Most surveys were conducted in Bangladesh (*n* = 123, 36.5%), followed by Thailand (*n* = 98, 29.1%), Laos (*n* = 41, 12.2%), Cambodia (*n* = 37,11.0%), and Myanmar (*n* = 33, 9.8%). The majority of surveys had rural coverage, including surveys conducted only in rural areas (*n* = 131, 38.8%), nationally representative surveys (*n* = 78, 23.1%), and surveys conducted in both rural and urban areas (*n* = 50, 14.8%). Fewer surveys (*n* = 34, 10.1%) had urban only coverage, and 45 (13.4%) surveys did not specify the coverage. Across countries, majority surveys (32.5 -54.1%) had rural only coverage; 17.1-30.3% surveys were national representative; and less than 10% of surveys had urban only coverage, except for Bangladesh (*n* = 24, 19.4%)( Appendix S4).

Most surveys (*n* = 201) studied conditions among participants in their adulthood, including adult (*n* = 107), of reproductive age (*n* = 29), reproductive age and older adult (*n* = 27), and older adult and retirement age (*n* = 48). Seventy-one surveys were done among participants in their childhood, including child (*n* = 13), preschool-children (*n* = 50), and school-age-children (*n* = 13). Seventy-one surveys were conducted participants’ age across the lifespan, and 25 surveys did not specify participants’ age.

Non-communicable conditions were reported by 205 surveys, followed by infectious conditions (*n* = 124), disability (*n* = 49), symptoms (*n* = 45), general health (*n* = 35), and self-perceived health (*n* = 34) .

While 70.0% (*n* = 236) of surveys studied conditions from a single health condition category, 18.1% (*n* = 61) studied two categories, and 11.9% (*n* = 40) studied three to five categories. The majority of surveys studied non-communicable conditions (*n* = 119, 58.0%) and infectious conditions (*n* = 90, 72.6%), studied conditions from a single health conditions category. In contrast, most surveys studying symptoms (*n* = 40, 88.9%), disability (*n* = 42, 85.7%), self-perceived health (*n* = 27, 79.4%) and general health (*n* = 27, 77.1%), also studied conditions of other categories.

The most studied conditions across age groups and condition categories are: undernutrition (*n* = 77), obesity (*n* = 75), hypertension (*n* = 60), diabetes (*n* = 56), and intestinal parasites (*n* = 38).

### Non-communicable conditions

**Fig. 2:**
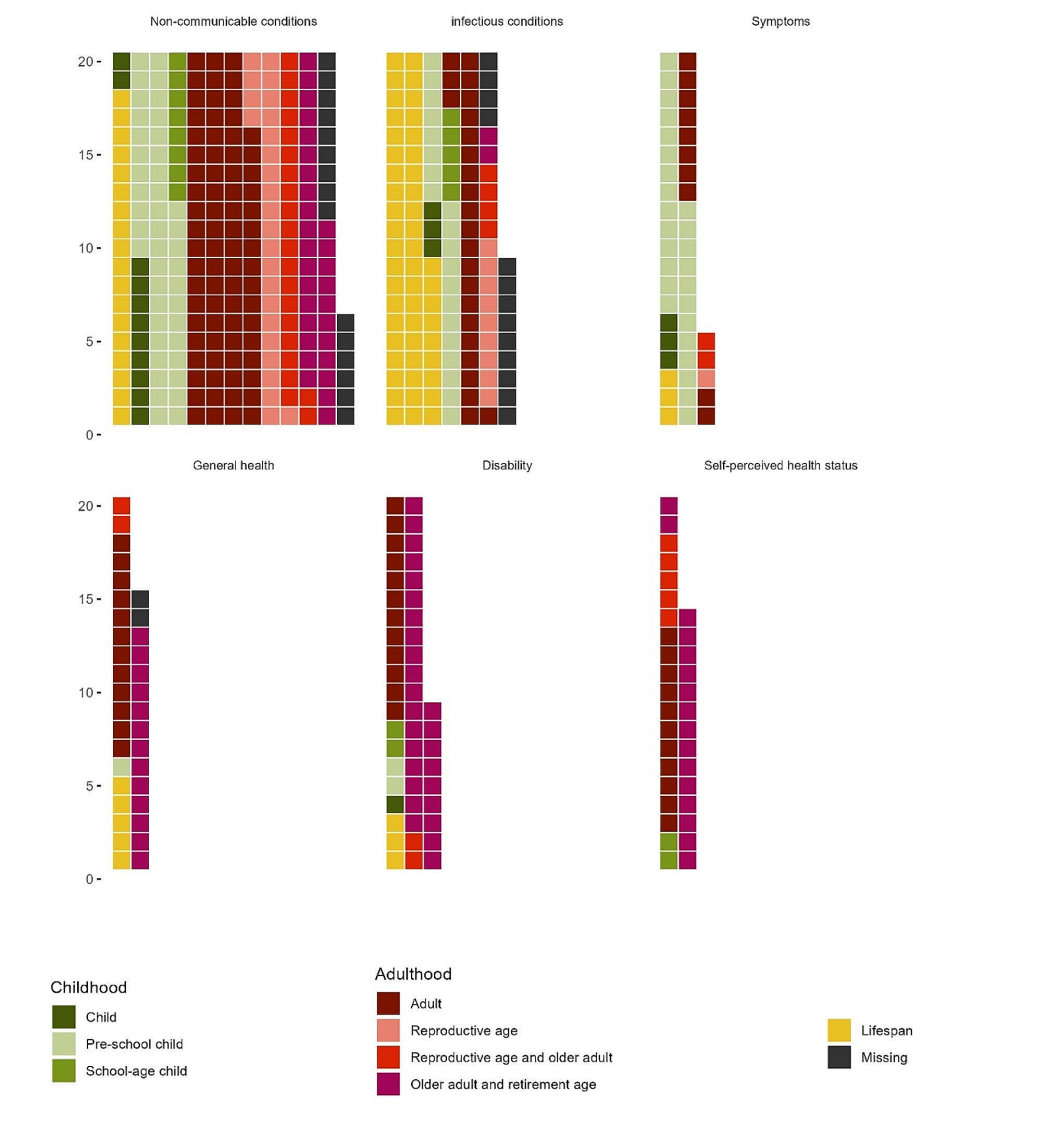
Waffle plot showing the distribution of health conditions by age categories. Figure showing (from the top left to bottom right) the number of surveys studied among participants of each age group, on Non-communicable conditions, Infectious conditions, Symptoms and abnormal findings (Symptoms), General health condition inquiries (General health), Disability and functional limitation (Disability), and Self-perceived health status Each square represents one survey. However, there are more squares than the total number of surveys, because multiple age groups can be studied by a survey. The number of squares of each condition category are: non-communicable condition (*n* = 236), infectious condition (*n* = 129), Symptoms (*n* = 45), General health (*n* = 35), disability (*n* = 49), to self-perceived health status (*n* = 34)

Non-communicable conditions were those most studied across countries (Bangladesh: *n* = 94, Thailand: *n* = 54, Cambodia: *n* = 20, Myanmar: *n* = 17), except Laos (*n* = 16), and geographic coverages (rural: *n* = 69, national representative: *n* = 61, rural and urban: *n* = 30, and urban: *n* = 22). They were studied the most among participants in adults (*n* = 76) and preschool child (*n* = 43) age categories (Fig. 2). The most studied non-communicable conditions are described in Table 2.

**Table 2:** The top 5 most studied conditions of each health condition category, by age groups

Overall across age groups	Lifespan	Childhood	Adulthood	Missing
**Non-communicable conditions**				
Undernutrition (*n* = 77) Obesity (*n* = 75) Hypertension (*n* = 60) Diabetes (*n* = 56) Anemia(*n* = 30)	Injury- animal cause (*n* = 3) Injury- electrocution cause (*n* = 2) Epilepsy/seizure (*n* = 2) Injury-non-specific cause (*n* = 2) Anemia (*n* = 1) Injury- eye (*n* = 1) Depression (*n* = 1) Micronutrition deficiency (*n* = 1) Obesity (*n* = 1) Periodontal conditions (*n* = 1) Psoriasis (*n* = 1) Suicidality (*n* = 1) Teeth related oral health condition (*n* = 1)	Undernutrition (*n* = 45) Obesity (*n* = 16) Anemia (*n* = 11) Micronutrition deficiency (*n* = 4) Hemoglobinopathy (*n* = 3) Aggressive behavior (*n* = 3)	Hypertension (*n* = 56) Obesity (*n* = 56) Diabetes (*n* = 54) Undernutrition (*n* = 39) Hyperlipidemia (*n* = 22)	Obesity (*n* = 3) Undernutrition (*n* = 3) Hypertension (*n* = 3) Depression (*n* = 3) Anxiety (*n* = 3)
**Infectious conditions**				
Intestinal parasites (*n* = 38) Malaria(*n* = 32) Acute respiratory infection (*n* = 13) Tuberculosis (*n* = 11) Hepatitis B virus (*n* = 11)	Malaria(*n* = 20) Intestinal parasites (*n* = 18) Soil-transmitted-helminth (*n* = 2) Hookworm (*n* = 2) Tapeworm (Taeniasis, Cysticercosis) (*n* = 2) Dengue (*n* = 2) Japanese encephalitis (*n* = 2) Hepatitis B virus (*n* = 2)	Acute respiratory infection (*n* = 12) Intestinal parasites (*n* = 7) Malaria(*n* = 6) Tuberculosis (*n* = 3) Hepatitis B virus (*n* = 2) Pneumonia (*n* = 2)	Intestinal parasites (*n* = 8) Tuberculosis (*n* = 7) Hepatitis C virus (*n* = 7) Malaria(*n* = 5) Hepatitis B virus (*n* = 5)	Intestinal parasites (*n* = 5) Hepatitis B virus (*n* = 2) Hepatitis C virus (*n* = 1) Malaria (*n* = 1) Dengue (*n* = 1) Acute respiratory infection (*n* = 1) Trematodes (O. viverrini, H. taichui) (*n* = 1) Trematodes (O. viverrini) (*n* = 1)
**Symptoms**				
Diarrhea (*n* = 25) Fever (*n* = 19) Cough(*n* = 7) Proteinuria (*n* = 2) Shortness of breath (*n* = 2) Impaired lung function (*n* = 2) Wheezing (*n* = 2)	Fever (*n* = 2) Diarrhea (*n* = 1)	Diarrhea (*n* = 24) Fever (*n* = 16) Cough (*n* = 5) Shortness of breath (*n* = 1) Multiple symptoms of digestive system (*n* = 1) Palpable spleen (*n* = 1)	Cough (*n* = 2) Impaired lung function (*n* = 2) Proteinuria (*n* = 2) Wheezing (*n* = 2) Constipation (*n* = 1) Dizziness (*n* = 1) Shortness of breath (*n* = 1) Fever (*n* = 1) Headache (*n* = 1) Presence of inflammation (*n* = 1) Raised serum creatinine (*n* = 1) Abnormal pulse rate (*n* = 1) Weakness (*n* = 1)	-
**Disability**				
Activity of daily living (*n* = 16) Function- &Activity- related (*n* = 10) Function-Vision (*n* = 7) Non-specified (*n* = 6) Body structure- &function-related (*n* = 5)	Function- &activity- related (*n* = 2) Body-structure (*n* = 1)	Activity- Oral health related (*n* = 2) Function- & activity- related (*n* = 2) Activity of daily living (*n* = 1) Body structure- & function related (*n* = 1)	Activity of daily living (*n* = 15) Function- Vision (*n* = 7) Function- &Activity-related (*n* = 6) Non-specified (*n* = 6) Body structure- &function- related (*n* = 4) Function-related (*n* = 4)	-
**Self-perceived health status**				
Quality of life (*n* = 20) Self-rated health (*n* = 19)	-	Quality of life (*n* = 2) Self-rated health (*n* = 1)	Quality of life (*n* = 18) Self-rated health (*n* = 18)	-

### Infectious diseases

Infectious conditions were those most studied in Laos (*n* = 28) and Cambodia (*n* = 20). They were also studied by about half of the surveys conducted in Thailand (*n* = 41) and Myanmar (*n* = 13), and surveys with rural only coverage (*n* = 62), and unspecified coverage (*n* = 21). They were studied most among participant age across the lifespan (*n* = 49), adult (*n* = 24) and preschool child (*n* = 20) categories (Fig. 2). Infectious conditions were categorized into parasites (*n* = 83), viruses (*n* = 23), bacteria (*n* = 14), and unspecified pathogens (*n* = 17).

### Symptoms

Symptoms were studied by relatively larger number of survey from Laos (*n* = 10) and Myanmar (*n* = 7), and surveys with national representative samples (*n* = 20), compare surveys conducted in other countries, especially in Thailand (*n* = 2), and geographic coverage. Symptoms were studied mainly among preschool children (*n* = 26) (Fig. 2).

### Disability, self-perceived health status, and general health

While general health, disability, and self-perceived status were studied by fewer surveys across countries and geographic coverage compare with other condition categories, relatively more surveys studied on these categories were identified from Myanmar (self-perceived health status (*n* = 7), general health (*n* = 6), and disability (*n* = 7). Disability specifically, was studied by relatively more surveys conducted in Thailand (*n* = 20), with nationally representative samples (*n* = 22), or rural and urban coverage (*n* = 10).

Older adult and retirement age were the most common study population for general health (*n* = 13), disability (*n* = 27), and self-perceived health (*n* = 16) (Fig. 2). In addition, adult populations were also the common target group for surveys of general health (*n* = 12) and self-perceived health (*n* = 11) .

### Leading causes of disability and death

**Fig. 3:**
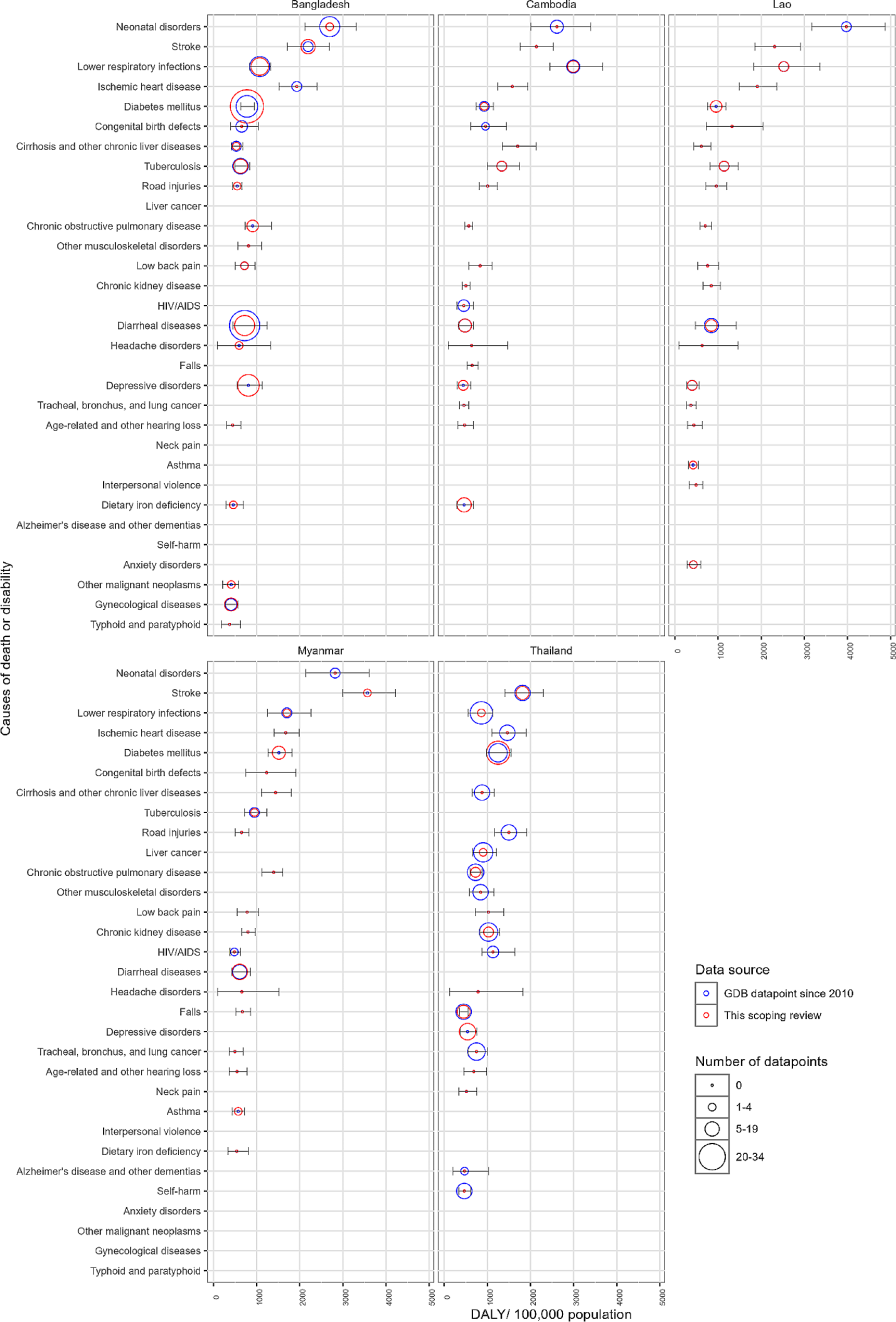
Available datapoints and surveys covering the top 20 causes of disability and death according to disability-adjusted-life-years (DALY) rate from the Global Burden of Disease Study (GBD) 2019, by study country. The size of circle indicating number of available data source, with blue indicating data source used in the GBD study and collected since 2010, and red indicated the number of surveys included in this review. The position of the circles on x axis represents the DALY rate (100,000 population), and the bars on the circles represent the 95% confidence interval of DALY rate of each cause from the GBD study The top 20 high-burden causes vary across countries. Common top-burden causes across all countries include: lower respiratory infections, diabetes mellitus, cirrhosis and other chronic liver diseases, ischemic heart disease, stroke, road injuries, chronic obstructive pulmonary disease, low back pain, headache disorders, age-related and other hearing loss; across 4 countries include: diarrheal diseases, tuberculosis, tracheal, bronchus, and lung cancer, congenital birth defects, chronic kidney disease, neonatal disorders, and depressive disorders. Other causes include: HIV/AIDS, falls, dietary iron deficiency, other musculoskeletal disorders, asthma, liver cancer, gynecological diseases, self-harm, Alzheimer’s disease and other dementias, typhoid and paratyphoid, interpersonal violence, anxiety disorders, other malignant neoplasms, and neck pain

From the GBD study, the top 20 causes with the highest DALY rate largely overlapped across study countries (Fig. 3). Among the 20 top burden causes of Bangladesh, 15 causes were studied by at least one survey included in this review, and it is 6 for Cambodia, 7 for Laos, 6 for Myanmar, and 8 for Thailand. According to the references used in GBD, 11 causes from Bangladesh had available datapoints, 7 for Cambodia, 6 for Laos, 5 for Myanmar, and 15 for Thailand.

Consistent findings were observed from both the surveys included in this review and the metadata from the GBD regarding the study coverage of common top burden causes across the countries. Diabetes, respiratory infections, tuberculosis, and diarrheal disease showed good study coverage. They have been studied by at least one survey, and identified with datapoints in GBD metadata, from all or most affected countries. In contrast, headache disorders, hearing loss, and lower back pain were rarely studied by the surveys included in the review, and had little or no data available from GBD metadata from the affected countries.

Discrepancies in study coverage were also recognized. Other common causes, including ischemic heart disease, chronic liver diseases, tracheal/ bronchus/ lung cancer, congenital birth defects, neonatal disorders, and road injuries were studied by a few or no surveys. However, GBD metadata included more datapoints for these causes, especially from Thailand and Bangladesh. Whereas depressive disorder, despite having only 2 datapoints from GBD metadata available from Laos, was among the most commonly studied conditions in the surveys (*n* = 24) and in all countries.

## Discussion

This scoping review included 337 community-based and population-based cross-sectional surveys conducted in Bangladesh, Cambodia, Laos, Myanmar, and Thailand published between 2010 and 22. This review highlighted firstly that the primary focus of these surveys are known high-burden causes of death and disability aligning with the recent GBD estimates, with gaps on conditions that relate to aging populations and other demographic changes in the region. Secondly, that little has been studied outside of the known regional high-burden diseases. The findings suggested while continuous monitoring well-studied high burden diseases is important for tracking the progress of the current intervention strategies, the research focus of cross-sectional surveys could expend to addressing the knowledge gaps.

Non-communicable conditions are among the leading causes of morbidity and mortality across these countries according to GBD estimates [18, 31]. They were also studied in the majority of surveys from each country. Risk factors attributable to cardiovascular disease (i.e. hypertension, underweight, and obesity) and diabetes are the most studied and are among the causes contribute to the most significant loss of DALYs. The surveys also extensively covered regionally high-burden infectious diseases, including tuberculosis, acute respiratory infection, and diarrhea. Data on these causes were also recorded from the sources beyond the prevalence surveys we reviewed (e.g. censuses, vital statistics, and other health-related data), as indicated by the rich references used in GBD study which include a wider range of data source [33]. While it is still important to continuously collect data on these conditions to monitor progress of the disease control and intervention strategies, considering the existing data sources for these conditions, the scope of community-based surveys could expand to other disease and health conditions.

The scope of future cross-sectional surveys could fill gaps in knowledge by including some of the regional high-burden causes that are predicted to increase prevalence due to the aging population and socioeconomic changes in the regions, but have not yet been widely studied [34–36]. This includes hearing loss, headache disorder, and lower back pain. Gaps in the survey, and little data used in GBDs suggest that current research and other data collection methods have potentially overlooked other emerging risk factors. Population groups with rising risks but are not considered high-risk previously for certain diseases could be included in future surveys. For example, with an evolving lifestyle and increasing prevalence of obesity among children, the prevalence of diabetes is rising in younger populations [37]. Children or adolescents could be the target population in surveys studying diabetes, whereas hitherto adults were the focus of the recent surveys. More attention could also be directed towards the elderly to understand their health problems. Compared to the other countries there were many surveys from Thailand which studied the elderly population by evaluating their activity of daily living. This is likely attributable to Thailand’s more rapid aging population [38]. However, countries with younger population structures such as Cambodia and Laos could also benefit from the health data from the elderly to better anticipate their future healthcare needs [38]. Including these conditions can fill gaps in current population-level disease estimates, and help health systems prepare for demographic change.

Some important causes of DALYs are not suitable to be studied directly by a cross-sectional survey design, albeit prevalence of known risk factors or early stage manifestation of disease can potentially be surveyed in community settings. Such conditions include ischemic heart disease, liver cirrhosis, chronic kidney disease, and tracheal, bronchus and lung cancer, have resource demanding diagnosis criteria, often rapid mortality, and were therefore studied by limited number of surveys. However, the risk factors of some of them were commonly studied. For instance, chronic viral hepatitis as a known risk factor for liver cirrhosis are with well-established point-of-care tests and procedures has surveyed [39, 40]. Depression is one of the most studied conditions in surveys, but limited data were use in GBD. It is can be explained by the questionnaire-based screening tools often used in surveys versus a strict diagnosis criteria for depressive disorder applied in GBD [41, 42]. This type of data will not only provide background information on the diseases but also support the detection of diseases at an early stage, informing optimal strategies for prevention and control, proving to be a more cost-effective public health approach [43, 44].

Other less studied high-burden causes in surveys can be explained by their low-case-number in population (e.g. neonatal disorders and congenital birth defect), which can be more efficiently studied in health facility-based studies, or using existing population data source such as vital registration, or police report (e.g. road traffic injury). It is less critical to incorporating these conditions in future cross-sectional surveys, although, collecting data on knowledge, attitudes, and behavior around such conditions may enrich the understanding and potentially improve the population-level estimations by addressing underreporting and health service utilization patterns [45, 46].

In addition to the known high-burden diseases, the scope of surveys could be broadened to include other regionally important diseases and explore potential unknown risk factors for high-burden diseases. Neglected tropical diseases (NTD), especially those related to non-malarial febrile illnesses (e.g. scrub typhus, melioidosis), still contribute significantly to morbidity and mortality in parts of the region [12, 47]. However, except for intestinal parasites, NTDs have not been well studied in the surveys identified in this review. This may be due to the challenge of capturing an acute infection within a limited data collection period through cross-sectional surveys. Despite the challenges, seroprevalence studies have proven useful in rapidly generating data on disease transmission (e.g. for COVID-19, or dengue) [48–50]. Current studies often rely on self-reported information on fever and related diseases (e.g., Acute respiratory infection), with the etiology often absent. This presents an opportunity to utilize seroprevalence surveys to detect the hidden burden of the causes of fever, and provide efficient and scalable approaches to generate population-based incidence data in resource-limited settings [51, 52].

While expanding the current research focus could pose challenges in resource limited settings, questionnaire-based evaluations which can collect data on a board range of health problems within limited time could be accessible and efficient means for surveys, compared to the resource-intense examinations and tests [53]. General health inquiries can offer direct prevalence data on the full range of existing health problems; evaluations on self-perceived health and disability can provide a comprehensive picture of health, though lacking specificity, can predict morbidity, mortality, and future healthcare needs [54–57]. In addition, analyzing the data with the results of a clinical diagnosis or objective measures (e.g. laboratory results) can aid in the identification of determinants (e.g. disease status, socioeconomic status) of self-perceived health, and thus informing public health planning for an improved self-perceived health status [58]. However, despite their utilization in surveys across countries, the inclusion of these measures remains relatively low. Most of the surveys targeting on non-communicable conditions and infectious conditions were not incorporated these measures, and is only commonly studied among the older adulthood and retirement age groups. It presents an opportunity to consistently incorporate the measures in future research and apply them to wider population.

### Limitations

Our study is limited by its nature of secondary data analysis. Significant heterogeneity of definitions of health conditions were applied across studies, an exact match to a specific internal classification guidelines was not always possible. Therefore, we have primarily followed the ICPC-3 in the categorization of health conditions, while allowing for content appropriate difference that due to variances of definitions used across studies. When matching the health conditions with the causes of the death and disability from GBD similar principles applied.

Another limitation was the lack of sufficient study data to map geographically where surveys took place. We were not able to constantly distinguish between urban and rural study locations, as about 13% of the studies did not specify the urbanicity of the study location from all related publications. Meanwhile, the names of the study area were sometimes ambiguous (e.g. the same name can refer to a city and a district). Consequentially, this limitation restricted our ability to determine the sample representativeness of each study and to establish the association between quantity and quality of studies on each health aspects. A higher number of surveys covering certain health conditions does not necessarily indicate robust surveillance of those conditions. However, the findings of this scoping review achieved its aim of depicting the health spectrum studied in these surveys and to identify knowledge gaps for future studies. As the next step, systematic reviews evaluating the sufficiency of surveillance for diseases studied by a high number of surveys could be conducted, including critical appraisal of study quality and more extensive data synthesis. Following the inclusion criteria, studies using health facilities or school-based recruitment were not included in the review. This omission partially explains the gaps in reported health conditions related to maternal and neonatal health, as well as among school-aged children. however, the inclusion criteria include only community-based studies, in which chiefly healthy participants were recruited, and avoids studies with potential selection bias such as facility-based surveys.

The inclusion criteria also restricted to quantitative studies that reported the prevalence of at least a health condition. Although community-based qualitative studies exist, their research methods and outcomes differ from those of quantitative studies. To maintain the focus of our review and to guide future prevalence surveys, we included only quantitative studies.

### Implications

Cross-sectional surveys are an efficient method to obtain population-based data in Southeast Asia, where the epidemiology of many conditions is incomplete and the coverage of health reporting and surveillance systems is limited. While regionally high-burden diseases deserve continuous attention to monitor trends and progress, increased attention could be given to address population-based epidemiology gaps, particularly those associated with the aging populations and socioeconomic and environmental changes. This includes known high-burden diseases- hearing loss, headache disorder, musculoskeletal pain, and other chronic conditions, including cancer, and potentially emerging risk factors or high-burden diseases that can be anticipated based on the evolving trend of epidemiology in the region. Meanwhile, regional relevant diseases such as non-malaria febrile illness, have been understudied by prevalence surveys.

To overcome the limitations of cross-sectional surveys, and address resource constraints, some study approaches and evaluation methods could be applied. Symptoms or early onset of diseases, indications of infection (i.e. serological responses) that are suitable to be studied in the field could be surveyed; questionnaire-based evaluation, including self-reported health conditions, self-perceived health and disability, that can provide improved disease estimates cost-effectively, could also be used in surveys. This expansion of study scope may inform new areas for disease prevention and control priorities at an early stage and prepare health system readiness to meet modern needs.

## Conclusion

Rich prevalence data is available for the traditionally high burden of diseases in the region, such as diabetes or tuberculosis. However, knowledge gaps were identified among conditions that with high or emerging burden related to the dynamic epidemiology of the region. The findings of this review suggest that future surveys and other data collection methods could expand their scope to new and under-studied conditions, to provide improved population-level prevalence estimates and to reflect the evolving regional epidemiology. Through better disease prevalence estimates, evidence-based resource allocation can be made to direct interventions and health system development in Southeast Asia.

### Electronic supplementary material

Below is the link to the electronic supplementary material.

Supplementary Material 1

Supplementary Material 2

## Data Availability

Data is provided within the manuscript or supplementary information files.

## Abbreviations

DALY: Disability-adjusted-life-years
DHS: Demographic and health survey
GBD: Global Burden of Disease Study
MeSH: Medical Subject Headings
NTD: Neglected tropical disease
OSF: Open Science Framework
PRISMA-ScR: Preferred Reporting Items for Systematic Review and Meta-analysis Extension for Scoping Review
