# Supplementary material for: The spectrum of health conditions in community-based cross-sectional surveys in Southeast Asia 2010-21: a scoping review
Source: BMC Public Health. 2024 Jul 11;24:1853. doi: 10.1186/s12889-024-19347-3 (PMC11238468; doi:10.1186/s12889-024-19347-3)
Supplement: Supplementary file 1 — Supplementary Material 1 [file 12889_2024_19347_MOESM1_ESM.docx]

# S1. Database search terms

**Database: Medline (Ovid MEDLINE® Epub Ahead of Print, In-Process & Other Non-Indexed Citations, Ovid MEDLINE® Daily and Ovid MEDLINE®) 1946 to present**

Search Strategy:

--------------------------------------------------------------------------------

1 asia, southeastern/ or cambodia/ or laos/ or myanmar/ or thailand/ (42762)

2 asia, western/ or bangladesh/ (14281)

3 (thai* or cambodia* or kampudja or khmer or lao* or myanmar or burma or burmese or Bangladesh* or bengali or bangla or "south asia*" or "southeast asia*" or "south-east asia*").ti,ab. (91738)

4 1 or 2 or 3 (104285)

5 exp Health Surveys/ (594676)

6 ("health survey*" or (("health care" or healthcare) adj2 (surveillance or registration or "quality control")) or "population surveillance" or "public health surveillance" or "health status indicator*" or "chronic disease indicator*" or "disease activity score*" or "global disease burden" or "organ dysfunction score*" or "patient acuity" or "severity of illness index").ti,ab. (48336)

7 5 or 6 (624053)

8 Residence Characteristics/ (35970)

9 exp Population/ (132070)

10 Family Characteristics/ (27587)

11 (community-based or community or population-based or population or household).ti,ab. (1999942)

12 8 or 9 or 10 or 11 (2097339)

13 4 and 7 and 12 (2892)

14 13 (2892)

15 limit 14 to yr="2010 -Current" (1808)

**Database: Medline (Ovid MEDLINE® Epub Ahead of Print, In-Process & Other Non-Indexed Citations, Ovid MEDLINE® Daily and Ovid MEDLINE®) 1946 to present**

Search Strategy:

--------------------------------------------------------------------------------

1 asia, southeastern/ or cambodia/ or laos/ or myanmar/ or thailand/ (42762)

2 asia, western/ or bangladesh/ (14281)

3 (thai* or cambodia* or kampudja or khmer or lao* or myanmar or burma or burmese or Bangladesh* or bengali or bangla or "south asia*" or "southeast asia*" or "south-east asia*").ti,ab. (91738)

4 1 or 2 or 3 (104285)

5 exp Health Surveys/ (594676)

6 ("health survey*" or (("health care" or healthcare) adj2 (surveillance or registration or "quality control")) or "population surveillance" or "public health surveillance" or "health status indicator*" or "chronic disease indicator*" or "disease activity score*" or "global disease burden" or "organ dysfunction score*" or "patient acuity" or "severity of illness index").ti,ab. (48336)

7 5 or 6 (624053)

8 Residence Characteristics/ (35970)

9 exp Population/ (132070)

10 Family Characteristics/ (27587)

11 (community-based or community or population-based or population or household).ti,ab. (1999942)

12 8 or 9 or 10 or 11 (2097339)

13 4 and 7 and 12 (2892)

14 13 (2892)

15 limit 14 to yr="2010 -Current" (1808)

**Database: Global Health <1973 to 2021 Week 34>**

Search Strategy:

--------------------------------------------------------------------------------

1 south east asia/ or myanmar/ or thailand/ (97452)

2 cambodia/ (3125)

3 laos/ (1821)

4 exp bangladesh/ or south asia/ (155719)

5 (thai* or cambodia* or kampudja or khmer or lao* or myanmar or burma or burmese or Bangladesh* or bengali or bangla or "south asia*" or "southeast asia*" or "south-east asia*").ti,ab. (54226)

6 1 or 2 or 3 or 4 or 5 (258027)

7 disease surveys/ (21009)

8 ("health survey*" or (("health care" or healthcare) adj2 (surveillance or registration or "quality control")) or "population surveillance" or "public health surveillance" or "health status indicator*" or "chronic disease indicator*" or "disease activity score*" or "global disease burden" or "organ dysfunction score*" or "patient acuity" or "severity of illness index").ti,ab. (18165)

9 7 or 8 (38754)

10 communities/ (15580)

11 populations/ (2864)

12 households/ (23547)

13 (community-based or community or population-based or population or household).ti,ab. (539288)

14 10 or 11 or 12 or 13 (547167)

15 6 and 9 and 14 (2316)

16 15 (2316)

17 limit 16 to yr="2010 -Current" (1778)

**CINAHL EBSCOhost**

| **#** | **Query** | **Results** |
| --- | --- | --- |
| S13 | S4 AND S7 AND S11 | 951 |
| S12 | S4 AND S7 AND S11 | 1,200 |
| S11 | S8 OR S9 OR S10 | 945,720 |
| S10 | TI ( community-based or community or population-based or population or household ) OR AB ( community-based or community or population-based or population or household ) | 679,311 |
| S9 | (MH "Population+") | 331,729 |
| S8 | (MH "Communities+") | 52,930 |
| S7 | S5 OR S6 | 166,789 |
| S6 | TI ( "health survey*" or (("health care" or healthcare) n2 (surveillance or registration or "quality control")) or "population surveillance" or "public health surveillance" or "health status indicator*" or "chronic disease indicator*" or "disease activity score*" or "global disease burden" or "organ dysfunction score*" or "patient acuity" or "severity of illness index" ) OR AB ( "health survey*" or (("health care" or healthcare) n2 (surveillance or registration or "quality control")) or "population surveillance" or "public health surveillance" or "health status indicator*" or "chronic disease indicator*" or "disease activity score*" or "global disease burden" or "organ dysfunction score*" or "patient acuity" or "severity of illness index" ) | 21,447 |
| S5 | (MH "Surveys") | 151,021 |
| S4 | S1 OR S2 OR S3 | 26,230 |
| S3 | TI ( thai* or cambodia* or kampudja or khmer or lao* or myanmar or burma or burmese or Bangladesh* or bengali or bangla or "south asia*" or "southeast asia*" or "south-east asia*" ) OR AB ( thai* or cambodia* or kampudja or khmer or lao* or myanmar or burma or burmese or Bangladesh* or bengali or bangla or "south asia*" or "southeast asia*" or "south-east asia*" ) | 23,025 |
| S2 | (MH "Bangladesh") OR (MH "Asia, Western") | 4,762 |
| S1 | (MH "Asia, Southeastern") OR (MH "Cambodia") OR (MH "Laos") OR (MH "Myanmar") OR (MH "Thailand") | 11,430 |

**Web of Science Core Collection**

thai* or cambodia* or kampudja or khmer or lao* or myanmar or burma or burmese or Bangladesh* or bengali or bangla or "south asia*" or "southeast asia*" or "south-east asia*" (Topic) and ("health survey*" or (("health care" or healthcare) Near/2 (surveillance or registration or "quality control")) or "population surveillance" or "public health surveillance" or "health status indicator*" or "chronic disease indicator*" or "disease activity score*" or "global disease burden" or "organ dysfunction score*" or "patient acuity" or "severity of illness index") (Topic) and community-based or community or population-based or population or household (Topic) and 2021 or 2020 or 2019 or 2018 or 2017 or 2011 or 2012 or 2013 or 2014 or 2015 or 2016 or 2010 (Publication Years)

**SCOPUS**

( TITLE-ABS-KEY ( thai* OR cambodia* OR kampudja OR khmer OR lao* OR myanmar OR burma OR burmese OR bangladesh* OR bengali OR bangla OR "south asia*" OR "southeast asia*" OR "south-east asia*" ) AND TITLE-ABS-KEY ( ( "health survey*" OR ( ( "health care" OR healthcare ) W/2 ( surveillance OR registration OR "quality control" ) ) OR "population surveillance" OR "public health surveillance" OR "health status indicator*" OR "chronic disease indicator*" OR "disease activity score*" OR "global disease burden" OR "organ dysfunction score*" OR "patient acuity" OR "severity of illness index" ) ) AND TITLE-ABS-KEY ( community-based OR community OR population-based OR population OR household ) ) AND ( LIMIT-TO ( PUBYEAR , 2021 ) OR LIMIT-TO ( PUBYEAR , 2020 ) OR LIMIT-TO ( PUBYEAR , 2019 ) OR LIMIT-TO ( PUBYEAR , 2018 ) OR LIMIT-TO ( PUBYEAR , 2017 ) OR LIMIT-TO ( PUBYEAR , 2016 ) OR LIMIT-TO ( PUBYEAR , 2015 ) OR LIMIT-TO ( PUBYEAR , 2014 ) OR LIMIT-TO ( PUBYEAR , 2013 ) OR LIMIT-TO ( PUBYEAR , 2012 ) OR LIMIT-TO ( PUBYEAR , 2011 ) OR LIMIT-TO ( PUBYEAR , 2010 ) )

**WHO Global Index Medicus**

(tw:(thai* or cambodia* or kampudja or khmer or lao* or myanmar or burma or burmese or Bangladesh* or bengali or bangla or "south asia*" or "southeast asia*" or "south-east asia*")) AND (tw:("health survey*" or (("health care" or healthcare) and (surveillance or registration or "quality control")) or "population surveillance" or "public health surveillance" or "health status indicator*" or "chronic disease indicator*" or "disease activity score*" or "global disease burden" or "organ dysfunction score*" or "patient acuity" or "severity of illness index")) AND (tw:(community-based or community or population-based or population or household))

Limited: 2010 to 2021

**Google scholar**

(thai*|cambodia*|kampudja|khmer|lao*|myanmar|burma|burmese|Bangladesh*|bengali|bangla)("health survey*"|"health care surveillance”|”healthcare surveillance")(community-based|community|population-based|population|household)

<https://scholar.google.co.uk/scholar?hl=en&as_sdt=0%2C5&q=%28thai*%7Ccambodia*%7Ckampudja%7Ckhmer%7Clao*%7Cmyanmar%7Cburma%7Cburmese%7CBangladesh*%7Cbengali%7Cbangla%29%28%22health+survey*%22%7C%22health+care+surveillance%E2%80%9D%7C%E2%80%9Dhealthcare+surveillance%22%29%28community-based%7Ccommunity%7Cpopulation-based%7Cpopulation%7Chousehold%29&btnG>=

(thai*|cambodia*|kampudja|khmer|lao*|myanmar|burma|burmese|Bangladesh*|bengali|bangla)("population surveillance"|"public health surveillance"|"health status indicator*")(community-based|community|population-based|population|household)

<https://scholar.google.co.uk/scholar?hl=en&as_sdt=0%2C5&q=%28thai*%7Ccambodia*%7Ckampudja%7Ckhmer%7Clao*%7Cmyanmar%7Cburma%7Cburmese%7CBangladesh*%7Cbengali%7Cbangla%29%28%22population+surveillance%22%7C%22public+health+surveillance%22%7C%22health+status+indicator*%22%29%28community-based%7Ccommunity%7Cpopulation-based%7Cpopulation%7Chousehold%29&btnG>=

(thai*|cambodia*|kampudja|khmer|lao*|myanmar|burma|burmese|Bangladesh*|bengali|bangla)("public health surveillance"|"health status indicator*"|"chronic disease indicator*")(community-based|community|population-based|population|household)

<https://scholar.google.co.uk/scholar?hl=en&as_sdt=0%2C5&q=%28thai*%7Ccambodia*%7Ckampudja%7Ckhmer%7Clao*%7Cmyanmar%7Cburma%7Cburmese%7CBangladesh*%7Cbengali%7Cbangla%29%28%22public+health+surveillance%22%7C%22health+status+indicator*%22%7C%22chronic+disease+indicator*%22%29%28community-based%7Ccommunity%7Cpopulation-based%7Cpopulation%7Chousehold%29&btnG>=

(thai*|cambodia*|kampudja|khmer|lao*|myanmar|burma|burmese|Bangladesh*|bengali|bangla)("disease activity score*"|"global disease burden"|"organ dysfunction score*")(community-based|community|population-based|population|household)

<https://scholar.google.co.uk/scholar?hl=en&as_sdt=0%2C5&q=%28thai*%7Ccambodia*%7Ckampudja%7Ckhmer%7Clao*%7Cmyanmar%7Cburma%7Cburmese%7CBangladesh*%7Cbengali%7Cbangla%29%28%22disease+activity+score*%22%7C%22global+disease+burden%22%7C%22organ+dysfunction+score*%22%29%28community-based%7Ccommunity%7Cpopulation-based%7Cpopulation%7Chousehold%29&btnG>=

# S2. Publication screening and inclusion process

**Screening process:**

At each stage, publications were independently assessed by two reviewers. Any conflicts or discrepancies between reviewers were first attempted to be resolved through consensus discussions. Any remaining discrepancies were resolved by a third senior reviewer. The screen process and the responsible author initials are listed below (alphabetical order of initials):

1. Reviewers conducted Abstract screening:

- Pair one: MZ, NSNH
- Pair two: RC, MZ

1. Reviewers conducted Abstract screening of the references and citations of the inclusions from abstracts screening

- ARS, MZ

1. Reviewers conducted Full text screening

- Pair one: CA, MZ
- Pair two: HK, MZ
- Pair three: CA, HK
- Pair four: RC, SKM

Senior reviewers who had a role for resolving discrepancies: MZ, RC, NSNH, SKM

**Identification of primary data source (Survey)**

The original survey from which the data was primarily collected for each publication was identified, and all publications were grouped by "survey" (e.g. multiple publications used data collected from a particular DHS survey in Bangladesh). If the publications explicitly mentioned the primary data source, searches were conducted to locate the reports or publications where the data were originally published, within already included publications and beyond. Only those published within the same inclusion period of the review were additionally included. In cases where the name of the original study was not explicitly mentioned, publications reporting on the same population, with the same study design, and conducted during the same time period were considered part of the same original study and grouped together.

# S3 Age group categorizations and health condition definitions

**Table. Age group categorization and corresponding age criteria of included surveys**

| **Age groups (years)** | **Subcategories (years)** | **Age described in the study** |
| --- | --- | --- |
| **Lifespan** | **Lifespan** | All, <60, ≥0.5, ≥0.7, 0.5-60, ≥1 |
|  |  | ≥1.5, ≥2, ≥3, 5-86, ≥5, ≥6, ≥10, 12-82, 13-64, 14-90, |
| **Childhood (**≤19) | **Child (**≤19) | 0-18, 1-14, 2-6, 0.5-12, 0.5-6, 2-9 |
|  | **Preschool child(**≤**5)** | <0.5 month, <1 month, <1.5, <23 month, <2, ≤2, <4, <5, ≤5, 6-23.9 months 6-23 months, 0.5-2, 6-36 months, 6-47.9 months, 0.5-5, 6-59 months, 6-60 months, 6-69 months, 6-72 months, 12-35 months, 1-3, 12-47 months, 12-59 months, 1-5, 2-4, 24-59 months, 24-60 months, 3-5 |
|  | **School-age child (5-19)** | 4-14, 5, 5-9, 5-14, 6-12, 5-17, 6-14, 9-15, 10-16, 10-19, 11-15.3, 12, 15, 15-17 |
|  |  |  |
| **Adulthood (**≥15) | **Adult (**≥15) | ≥15, >15, 15-80, 15-99, 16-80, ≥16, 18-69, 18-70, ≥18, 20-70, 20-80, ≥20, ≥21, 25-74, ≥25, 30-69, ≥30, 34-77, ≥35, 40-74, 40-80, ≥40, >40 |
|  |  |  |
|  | **Reproductive age (14-49)** | **14-29**, 15-24, 15-34, 15-39, 15-45, 15-49, 16-25, 18-24, 18-30, 18-34, 18-45, 18-49, 20-45, 20-49, 35-44, 30-49 |
|  |  |  |
|  | **Reproductive age and older adulthood (15-69)** | 15-59, 15-64, 15-65, 16-55, 18-60, 18-64, 20-59, 20-60, 20-65, 25-54, 25-64, 30-59, 30-64, 35-64, 35-65, 40-59, 40-69 |
|  |  |  |
|  | **Older adulthood and retirement age (**≥50) | 50-65, ≥55, ≥50, ≥60, 60-75, 60-79, ≥65, 65-74 |

**Definition of health condition categories**

Non-communicable conditions reflecting the same underlying pathophysiology but representing different degrees of severity were grouped together(e.g. elevated blood glucose level, prediabetes, and diabetes were grouped as one type of health condition and referred to as “diabetes”).

Infectious conditions were categorized based on direct microbiologic identification of a pathogen (e.g., through culture or molecular tests) or indirect measures (e.g., serologic tests). Infective syndromes without a reported microbiologic cause, such as acute respiratory infection (ARI), were also categorized as infectious conditions. Distinctions were not made between present and past infections or between the species of the pathogens (e.g. infection with *Plasmodium falciparum and P. vivax* were both grouped as “malaria”). "Intestinal parasites" was used to categorize conditions that reported the presence of any parasites in fecal samples. When specific intestinal parasites were reported, the names of the parasites were included, such as "Intestinal parasite- Trematodes (*Opisthorchis viverrini*)." Infectious conditions were further subcategorized into bacterial, viral, parasitic, and non-specified based on the microbiological results or lack thereof.

Symptoms and abnormal findings that could not be determined as infectious or non-infectious causes (e.g., fever, diarrhea, abnormal pulse rate, raised serum creatine) were categorized as "Symptoms."

Conditions categorized as disability were either specified by the study or categorized by the review authors when a condition fit the WHO definition of disability, which includes impairments, activity limitations, and participation restrictions referring to the negative aspects of the interaction between an individual and the individual's contextual factors(1). Based on the aspects of disability, conditions were grouped into body structure, body function, and activities and participation related disability, according to the International Classification of Functioning, Disability and Health (2). Subcategorization of activity of daily living defined as fundamental skills required to independently care for oneself (3,4). It was applied, when multiple body function and activity related aspects were evaluated and/or reported as ADL by the included surveys.

Self-perceived health status encompassed the assessments of an individual's own opinion on their health, which can be generic, or disease-specific. They were grouped into two subcategories: self-rated health, which was based on a single-question assessment of self-rated health, and health-related quality of life, which was evaluated by instrument-specific assessment, such as SF12, EQ-5, or multi-item health status index derived from the World Health Survey (5–7).

General health describes the data collected from the questions asked to the survey participants, soliciting self-reported presence or history without limiting the answers to specific diseases or symptoms (e.g. E.g. Have you been ill in the past 30 days? Could you tell me all the symptoms or diagnosis you had?).

**References:**

1. Leonardi M, Bickenbach J, Ustun TB, Kostanjsek N, Chatterji S, MHADIE Consortium. The definition of disability: what is in a name? Lancet Lond Engl. 2006 Oct 7;368(9543):1219–21.

2. International Classification of Functioning, Disability and Health (ICF) [Internet]. [cited 2022 Nov 3]. Available from: https://www.who.int/standards/classifications/international-classification-of-functioning-disability-and-health

3. Katz S. Assessing self-maintenance: activities of daily living, mobility, and instrumental activities of daily living. J Am Geriatr Soc. 1983 Dec;31(12):721–7.

4. Edemekong PF, Bomgaars DL, Sukumaran S, Schoo C. Activities of Daily Living. In: StatPearls [Internet]. Treasure Island (FL): StatPearls Publishing; 2022 [cited 2022 Nov 10]. Available from: http://www.ncbi.nlm.nih.gov/books/NBK470404/

5. A single-vs. multi-item self-rated health status measure: a 21-country study [Internet]. [cited 2023 Feb 1]. Available from: https://apps.who.int/healthinfo/systems/surveydata/index.php/citations/52427

6. Gandek B, Ware JE, Aaronson NK, Apolone G, Bjorner JB, Brazier JE, et al. Cross-Validation of Item Selection and Scoring for the SF-12 Health Survey in Nine Countries: Results from the IQOLA Project. J Clin Epidemiol. 1998 Nov 1;51(11):1171–8.

7. Janssen MF, Szende A, Cabases J, Ramos-Goñi JM, Vilagut G, König HH. Population norms for the EQ-5D-3L: a cross-country analysis of population surveys for 20 countries. Eur J Health Econ. 2019;20(2):205–16.

# S4. Table. Number of studies conducted in each country, by rural, urban, and national representative coverage.

| Coverage* | Overall | Bangladesh | Thailand | Laos | Cambodia | Myanmar |
| --- | --- | --- | --- | --- | --- | --- |
| n(%) | 337 | 123 | 98 | 41 | 37 | 33 |
| National representative | 77 (22.8) | 32 (26.0) | 18 (18.4) | 7 (17.1) | 9 (24.3) | 10 (30.3) |
| Rural and urban | 50 (14.8) | 16 (13.0) | 16 (16.3) | 7 (17.1) | 3 (8.1) | 7 (21.2) |
| Urban | 34 (10.1) | 24 (19.5) | 7 (7.1) | 1 (2.4) | 1 (2.7) | 1 (3.0) |
| Rural | 131 (38.9) | 40 (32.5) | 34 (34.7) | 20 (48.8) | 20 (54.1) | 14 (42.4) |
| Not specified | 45 (13.4) | 11 (8.9) | 23 (23.5) | 6 (14.6) | 4 (10.8) | 1. (3.0) |

^*^Study conducted in multiple countries are not shown in the table. Including national representative: Laos and Thailand (n=1); Rural and urban: Laos and Thailand (n=1); Rural: Cambodia, Myanmar, and Thailand (n=1); Cambodia and Thailand (n=1): Laos and Thailand (n=1)


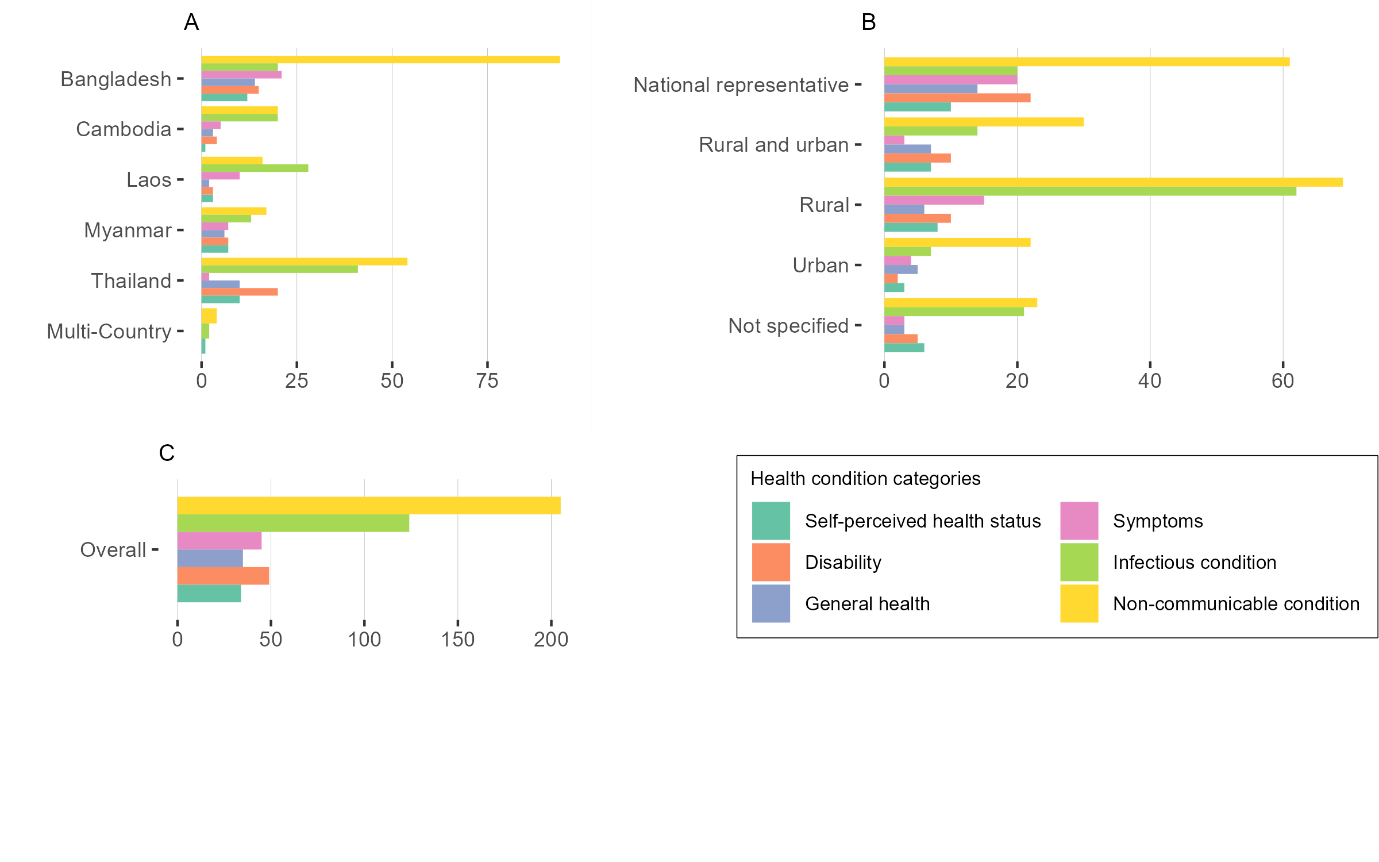


# **S5. Figure. Number of surveys by health condition category**

A. Number of surveys by health condition category and study country. B. Number of surveys reported by health condition category and by urban-rural coverage. C. Overall number of surveys by health condition categories.

Studies conducted in multiple countries include: Cambodia, Myanmar, and Thailand (n=1); Cambodia and Thailand (n=1); Laos and Thailand (n=3).
