# Supplementary material for: The spectrum of health conditions in community-based cross-sectional surveys in Southeast Asia 2010-21: a scoping review
Source: BMC Public Health. 2024 Jul 11;24:1853. doi: 10.1186/s12889-024-19347-3 (PMC11238468; doi:10.1186/s12889-024-19347-3)
Supplement: Supplementary file 2 — Supplementary Material 2 [file 12889_2024_19347_MOESM2_ESM.pdf]

Publication count	Citation	study_count	study_name	country_combine	rural_urban_num	categroy	subcategory	condition	age_group1	age_group2
377	Priest, J. W., et al. (2016). "Integration of Multiplex Bead Assays for Parasitic Diseases into a National, Population-Based Serosurvey of Women 15-39 Years of Age in Cambodia." PLoS neglected tropical diseases 10(5): e0004699.	1		Cambodia	Natioanl representative	NCD	nutritional status	micronutrition deficiency	adulthood	reproductive age
521	Wieringa, F. T., et al. (2016). "Low prevalence of iron and vitamin a deficiency among cambodian women of reproductive age." Nutrients 8(4): 197.					ID	bacteria	tetanus	adulthood	reproductive age
435	Scobie, H. M., et al. (2016). "Tetanus Immunity among Women Aged 15 to 39 Years in Cambodia: a National Population-Based Serosurvey, 2012." Clin Vaccine Immunol 23(7): 546-554.					ID	parasite	IP-STH (Strongyloidiasis)	adulthood	reproductive age
						ID	parasite	IP-tapeworm (cysticercosis)	adulthood	reproductive age
						ID	parasite	intestine parasite	adulthood	reproductive age
						ID	parasite	lymphatic filariasis	adulthood	reproductive age
						ID	parasite	malaria	adulthood	reproductive age
483	Tangtrakulwanich, B. and P. Suwanno (2012). "Epidemiology and risk factors of patellofemoral osteoarthritis in adults: a population-based study in southern Thailand." J Med Assoc Thai 95(8): 1048-1052.	2		Thailand	Rural and urban	NCD	musculoskeleton	Arthritis	adulthood	adulthood
						Disability/Lim	Function	Function Sensor	adulthood	adulthood
327	Muktabhant, B., et al. (2013). "Benefits and constraints in screening for non-communicable diseases in the rural area of Thailand's northeast." J Med Assoc Thai 96(6): 722-729.	3		Thailand	Rural	NCD	cvd/circulatory	elevated blood pressure/ hypertension	Missing	Missing
						NCD	endocrine and metabolic	elevated glucose/ prediabetes/ diabetes	Missing	Missing
						NCD	nutritional status	overweight/obese	Missing	Missing
386	Rahman, M. M., et al. (2013). "Self-reported illness and household strategies for coping with health-care payments in Bangladesh." Bull World Health Organ 91(6): 449-458.	4		Bangladesh	Urban	General health			adulthood	adulthood
145	Deeruksa, L. and K. Sanchaisuriya (2017). "Anemia in the Elderly in Northeastern Thailand: A Community-Based Study Investigating Prevalence, Contributing Factors, and Hematologic Features." Acta Haematol 138(2): 96-102.	5		Thailand	Rural	NCD	blood and blood forming organ	Anemia	adulthood	older adulthood and retirement age
						NCD	blood and blood forming organ	Hemoglobinpathy	adulthood	older adulthood and retirement age
244	Kang, Y., et al. (2021). "Perceived Health Status and its Correlates among Middle-Aged Laotians." Clin Nurs Res 30(8): 1202-1210.	6		Laos	Urban	NCD	cvd/circulatory	elevated blood pressure/ hypertension	adulthood	reproductive age and older adulthood
						SPH	srh	srh	adulthood	reproductive age and older adulthood
248	Kerkhof, K., et al. (2016). "Geographical patterns of malaria transmission based on serological markers for falciparum and vivax malaria in Ratanakiri, Cambodia." Malar J 15(1): 510.	7		Cambodia	Rural	ID	parasite	malaria	lifespan	lifespan
323	Mostafa Zaman, M., et al. (2020). "Prevalence of diabetes mellitus as obtained by nationwide screening in urban areas of Bangladesh." British Journal of Diabetes 20(1): 58-60.	8		Bangladesh	Urban	NCD	endocrine and metabolic	elevated glucose/ prediabetes/ diabetes	adulthood	adulthood

363	Phyo, S. W., et al. (2014). "Factors related to stunting status of the children aged uncer two years in Magway Township, Myanmar." Journal of Public Health and Development 12(3).	9		Myanmar	Rural and urban	NCD	nutritional status	undernutrition	childhood	Pre-school child
22	Akhter, A. (2011). "Prevalence of Diabetes Mellitus and its Associated Risk Indicators in a Rural Bangladeshi Population." The Open Diabetes Journal 4(1): 6-13.	10		Bangladesh	Not specified	NCD	endocrine and metabolic	elevated glucose/ prediabetes/ diabetes	adulthood	adulthood
524	Wongsaroj, T., et al. (2014). "Brief communication (Original). National survey of helminthiasis in Thailand." Asian Biomedicine 8(6): 779-783.	11		Thailand	Natioanl representative	ID	parasite	intestine parasite	lifespan	lifespan
529	Yong, T. S., et al. (2014). "Prevalence of intestinal helminths among inhabitants of Cambodia (2006-2011)." Korean J Parasitol 52(6): 661-666.	12		Cambodia	Not specified	ID	parasite	intestine parasite	childhood	School-age child
120	Chowdhury, F., et al. (2015). "Diarrheal Illness and Healthcare Seeking Behavior among a Population at High Risk for Diarrhea in Dhaka, Bangladesh." PLoS One 10(6): e0130105.	13		Bangladesh	Urban	Symptoms	digestive	diarrhea	lifespan	lifespan
431	Sato, M., et al. (2015). "Patterns of trematode infections of Opisthorchis viverrini (Opisthorchiidae) and Haplorchis taichui (Heterophyidae) in human populations from two villages in Savannakhet Province, Lao PDR." J Helminthol 89(4): 439-445.	14		Laos	Rural	ID	parasite	IP-tramatodes (O. viverrini, H. Taichu)	lifespan	lifespan
317	Mohammad, Q. D., et al. (2011). "Prevalence of stroke above forty years." Mymensingh Med J 20(4): 640-644.	15		Bangladesh	Not specified	NCD	cvd/circulatory	Stroke	adulthood	adulthood
97	Buathong, S., et al. (2020). "Genetic Differentiation of Opisthorchis-Like Eggs in Northern Thailand Using Stool Specimens Under National Strategic Plan to Control Liver Fluke Infection and Cholangiocarcinoma." Am J Trop Med Hyg 103(3): 1118-1124.	16		Thailand	Rural	ID	parasite	IP-tramatodes (O. viverrini, H. Taichu)	Missing	Missing
336	Nasreen, H. E., et al. (2010). "Low birth weight in offspring of women with depressive and anxiety symptoms during pregnancy: results from a population based study in Bangladesh." BMC Public Health 10: 515.	17		Bangladesh	Rural	NCD	psycological/ mental	anxiety	Missing	Missing
						NCD	psycological/ mental	depression	Missing	Missing
260	Khanam, F., et al. (2019). "Prevalence and Risk Factors of Cardiovascular Diseases among Bangladeshi Adults: Findings from a Cross-sectional Study." J Epidemiol Glob Health 9(3): 176-184.	18		Bangladesh	Natioanl representative	NCD	cvd/circulatory	Stroke	adulthood	adulthood
						NCD	cvd/circulatory	elevated blood pressure/ hypertension	adulthood	adulthood
						NCD	endocrine and metabolic	elevated glucose/ prediabetes/ diabetes	adulthood	adulthood
						NCD	nutritional status	overweight/obese	adulthood	adulthood
						Disability/Lim	Function	Function Sleep	adulthood	adulthood
460	Srithai, C., et al. (2021). "Prevalence of Opisthorchis viverrini and Its Associated Risk Factors in the Phon Sawan District of Nakhon Phanom Province, Thailand." Iran J Parasitol 16(3): 474-482.	19		Thailand	Not specified	ID	parasite	intestine parasite	adulthood	adulthood
314	Mistry, S. K., et al. (2021). "Older adults with non-communicable chronic conditions and their health care access amid COVID-19 pandemic in Bangladesh: Findings from a cross-sectional study." PLoS One 16(7): e0255534.	20		Bangladesh	Rural and urban	General health			adulthood	older adulthood and retirement age
324	Mridha, M. K., et al. (2021). "Prevalence and associated factors of depression among adolescent boys and girls in Bangladesh: findings from a nationwide survey." BMJ Open 11(1): e038954.	21		Bangladesh	Rural and urban	NCD	nutritional status	overweight/obese	childhood	School-age child

						NCD	psycological/ mental	depression	childhood	School-age child
129	Chuangchaiya, S., et al. (2019). "Prevalence and associated risk factors of Opisthorchis viverrini infections in rural communities along the Nam Kam River of Northeastern Thailand." Trop Biomed 36(1): 81-93.	22		Thailand	Rural	ID	parasite	IP-trematodes (O. viverrini)	adulthood	adulthood
353	Pansuwan, A., et al. (2011). "Anemia, iron deficiency and thalassemia among adolescents in Northeast Thailand: Results from two independent surveys." Acta Haematologica 125(4): 186-192.	23		Thailand	Not specified	NCD	blood and blood forming organ	Anemia	adulthood	reproductive age
						NCD	blood and blood forming organ	Hemoglobinpathy	adulthood	reproductive age
						NCD	nutritional status	micronutrition deficiency	adulthood	reproductive age
541	Zhao, Y., et al. (2018). "Risk factors for asymptomatic malaria infections from seasonal cross-sectional surveys along the China-Myanmar border." Malar J 17(1): 247.	24		Myanmar	Rural	ID	parasite	malaria	lifespan	lifespan
394	Rahman, M., et al. (2017). "Socio-demographic differences of disability prevalence among the population aged 60 years and over in Bangladesh." Asian Population Studies 14(1): 77-95.	25		Bangladesh	Natioanl representative	Disability/Lim	Body structure&function	Body structure&function	adulthood	older adulthood and retirement age
222	Iwagami, M., et al. (2017). "The detection of cryptic Plasmodium infection among villagers in Attapeu province, Lao PDR." PLoS Negl Trop Dis 11(12): e0006148.	26		Laos	Rural	ID	parasite	malaria	lifespan	lifespan
						Symptoms	multiple/non-specific	fever	lifespan	lifespan
342	Nguitragool, W., et al. (2017). "Very high carriage of gametocytes in asymptomatic low-density Plasmodium falciparum and P. vivax infections in western Thailand." Parasit Vectors 10(1): 512.	27		Thailand	Rural	ID	parasite	malaria	lifespan	lifespan
403	Rawal, L. B., et al. (2017). "Non-communicable disease (NCD) risk factors and diabetes among adults living in slum areas of Dhaka, Bangladesh." PLoS One 12(10): e0184967.	28		Bangladesh	Urban	NCD	cvd/circulatory	elevated blood pressure/ hypertension	adulthood	reproductive age and older adulthood
						NCD	endocrine and metabolic	elevated glucose/ prediabetes/ diabetes	adulthood	reproductive age and older adulthood
						NCD	nutritional status	overweight/obese	adulthood	reproductive age and older adulthood
150	Dunn, J. C., et al. (2017). "A cross-sectional survey of soil-transmitted helminthiases in two Myanmar villages receiving mass drug administration: epidemiology of infection with a focus on adults." Parasit Vectors 10(1): 374.	29		Myanmar	Rural	ID	parasite	IP-STH	lifespan	lifespan
361	Phoolcharoen, N., et al. (2017). "A population-based study of cervical cytology findings and human papillomavirus infection in a suburban area of Thailand." Gynecol Oncol Rep 21: 73-77.	30		Thailand	Not specified	NCD	genitourinary	abnormal cervical cytology	adulthood	adulthood
						ID	virus	hpv	adulthood	adulthood
500	Tripura, R., et al. (2017). "Submicroscopic Plasmodium prevalence in relation to malaria incidence in 20 villages in western Cambodia." Malar J 16(1): 56.	31		Cambodia	Rural	ID	parasite	malaria	adulthood	adulthood
369	Posuwan, N., et al. (2016). "The Success of a Universal Hepatitis B Immunization Program as Part of Thailand's EPI after 22 Years' Implementation." PLoS One 11(3): e0150499.	32		Thailand	Not specified	ID	virus	hbv	childhood	Pre-school child
146	DiMiceli, L. E., et al. (2016). "Methamphetamine use is associated with high levels of depressive symptoms in adolescents and young adults in Rural Chiang Mai Province, Thailand." BMC Public Health 16: 168.	33		Thailand	Rural	NCD	psycological/ mental	depression	lifespan	lifespan

307	Marella, M., et al. (2015). "Prevalence and correlates of disability in Bogra district of Bangladesh using the rapid assessment of disability survey." BMC Public Health 15: 867.	34		Bangladesh	Rural	Disability/Lim	Function &Activity	Function &Activity	adulthood	adulthood
153	Eom, K. S., et al. (2014). "Prevalence of helminthic infections among inhabitants of Lao PDR." Korean J Parasitol 52(1): 51-56.	35		Laos	Not specified	ID	parasite	intestine parasite	Missing	Missing
103	Chai, J. Y., et al. (2015). "Intestinal Helminths Recovered from Humans in Xieng Khouang Province, Lao PDR with a Particular Note on Haplorchis pumilio Infection." Korean J Parasitol 53(4): 439-445.	36		Laos	Rural	ID	parasite	malaria	lifespan	lifespan
452	Sohn, W. M., et al. (2014). "Prevalence of Haplorchis taichui among humans and fish in Luang Prabang Province, Lao PDR." Acta Trop 136: 74-80.	37		Laos	Rural	ID	parasite	intestine parasite	adulthood	adulthood
245	Karakochuk, C. D., et al. (2015). "Genetic hemoglobin disorders rather than iron deficiency are a major predictor of hemoglobin concentration in women of reproductive age in rural prey Veng, Cambodia." J Nutr 145(1): 134-142.	38		Cambodia	Rural	NCD	blood and blood forming organ	Anemia	adulthood	reproductive age
						NCD	blood and blood forming organ	Hemoglobinpathy	adulthood	reproductive age
						NCD	nutritional status	micronutrition deficiency	adulthood	reproductive age
						NCD	nutritional status	overweight/obese	adulthood	reproductive age
						NCD	nutritional status	undernutrition	adulthood	reproductive age
246	Karakochuk, C. D., et al. (2015). "The Homozygous Hemoglobin EE Genotype and Chronic Inflammation Are Associated with High Serum Ferritin and Soluble Transferrin Receptor Concentrations among Women in Rural Cambodia." J Nutr 145(12): 2765-2773.	39		Cambodia	Not specified	NCD	blood and blood forming organ	Anemia	adulthood	reproductive age
						NCD	blood and blood forming organ	Hemoglobinpathy	adulthood	reproductive age
						NCD	nutritional status	micronutrition deficiency	adulthood	reproductive age
94	Boonjaraspinyo, S., et al. (2013). "A cross-sectional study on intestinal parasitic infections in rural communities, northeast Thailand." Korean J Parasitol 51(6): 727-734.	40		Thailand	Rural	ID	parasite	intestine parasite	lifespan	lifespan
454	Songserm, N., et al. (2012). "Prevalence and co-infection of intestinal parasites among thai rural residents at high-risk of developing cholangiocarcinoma: a cross-sectional study in a prospective cohort study." Asian Pac J Cancer Prev 13(12): 6175-6179.	41		Thailand	Rural	ID	parasite	intestine parasite	adulthood	adulthood
71	Bartlett, E., et al. (2013). "Sources and prevalence of self-reported asthma diagnoses in adults in urban and rural settings of Bangladesh." Glob Public Health 8(1): 79-89.	42		Bangladesh	Rural and urban	NCD	respiratory	asthma	adulthood	adulthood
514	Wasitthankasem, R., et al. (2020). "Prevalence of Hepatitis C Virus in an Endemic Area of Thailand: Burden Assessment toward HCV Elimination." Am J Trop Med Hyg 103(1): 175-182.	43		Thailand	Rural and urban	ID	virus	hcv	adulthood	reproductive age and older adulthood
96	Buathong, S., et al. (2017). "Molecular discrimination of Opisthorchis-like eggs from residents in a rural community of central Thailand." PLoS Negl Trop Dis 11(11): e0006030.	44		Thailand	Rural	ID	parasite	IP-trematodes (O. viverrini)	Missing	Missing
512	Wasitthankasem, R., et al. (2017). "Assessment of hepatitis C virus infection in two adjacent Thai provinces with drastically different seroprevalence." PLoS One 12(5): e0177022.	45		Thailand	Not specified	ID	virus	hcv	adulthood	reproductive age and older adulthood

196	Huang, F., et al. (2017). "Prevalence of Clinical and Subclinical Plasmodium falciparum and Plasmodium vivax Malaria in Two Remote Rural Communities on the Myanmar-China Border." Am J Trop Med Hyg 97(5): 1524-1531.	46	Myanmar	Rural	ID	parasite	malaria	lifespan	lifespan
109	Chaiputcha, K., et al. (2015). "Prevalence and Risk Factors for Infection by Opisthorchis viverrini in an Urban Area of Mahasarakham Province, Northeast Thailand." Asian Pac J Cancer Prev 16(10): 4173-4176.	47	Thailand	Urban	ID	parasite	IP-trematodes (O. viverrini)	adulthood	adulthood
221	Islam, S. M., et al. (2015). "Prevalence of risk factors for hypertension: A cross-sectional study in an urban area of Bangladesh." Glob Cardiol Sci Pract 2015(4): 43.	48	Bangladesh	Urban	NCD	cvd/circulatory	elevated blood pressure/ hypertension	adulthood	adulthood
					NCD	nutritional status	overweight/obese	adulthood	adulthood
265	Khieu, V., et al. (2014). "High prevalence and spatial distribution of Strongyloides stercoralis in rural Cambodia." PLoS Negl Trop Dis 8(6): e2854.	49	Cambodia	Rural	ID	parasite	intestine parasite	lifespan	lifespan
266	Khieu, V., et al. (2014). "Prevalence and risk factors of Strongyloides stercoralis in Takeo Province, Cambodia." Parasit Vectors 7: 221.	50	Cambodia	Rural	ID	parasite	intestine parasite	lifespan	lifespan
451	Sohn, W. M., et al. (2012). "Prevalence of Opisthorchis viverrini infection in humans and fish in Kratie Province, Cambodia." Acta Trop 124(3): 215-220.	51	Cambodia	Rural	ID	parasite	intestine parasite	lifespan	lifespan
528	Yong, T. S., et al. (2012). "High prevalence of Opisthorchis viverrini infection in a riparian population in Takeo Province, Cambodia." Korean J Parasitol 50(2): 173-176.	52	Cambodia	Rural	ID	parasite	intestine parasite	lifespan	lifespan
240	Kaewpitoon, S. J., et al. (2012). "Prevalence of Opisthorchis viverrini infection in Nakhon Ratchasima province, Northeast Thailand." Asian Pac J Cancer Prev 13(10): 5245-5249.	53	Thailand	Not specified	ID	parasite	intestine parasite	lifespan	lifespan
232	Jiraanankul, V., et al. (2011). "Incidence and risk factors of hookworm infection in a rural community of central Thailand." Am J Trop Med Hyg 84(4): 594-598.	54	Thailand	Rural	ID	parasite	IP-STH (hookworm)	lifespan	lifespan
433	Sayasone, S., et al. (2011). "Helminth and intestinal protozoa infections, multiparasitism and risk factors in Champasack province, Lao People's Democratic Republic." PLoS Negl Trop Dis 5(4): e1037.	55	Laos	Rural	ID	parasite	intestine parasite	lifespan	lifespan
231	Jin, H., et al. (2021). "Prevalence and Risk Factors of Intestinal Helminthiasis in Remote Mountainous Villages of Northern Lao PDR: A Cross-Sectional Study." Korean J Parasitol 59(2): 131-138.	56	Laos	Rural	ID	parasite	intestine parasite	adulthood	adulthood
112	Chayaopas, N., et al. (2021). "The effective screening tools for detecting hearing loss in elderly population: HHIE-ST Versus TSQ." BMC Geriatr 21(1): 37.	57	Thailand	Rural and urban	NCD	cvd/circulatory	elevated blood pressure/ hypertension	adulthood	older adulthood and retirement age
					NCD	endocrine and metabolic	elevated cholesterol/ hyperlipidemia	adulthood	older adulthood and retirement age
					NCD	endocrine and metabolic	elevated glucose/ prediabetes/ diabetes	adulthood	older adulthood and retirement age
					Disability/Lim	Function	Function Hearing	adulthood	older adulthood and retirement age
388	Rahman, M. M., et al. (2021). "Frailty indexed classification of Bangladeshi older adults' physio-psychosocial health and associated risk factors- a cross-sectional survey study." BMC Geriatr 21(1): 3.	58	Bangladesh	Not specified	Disability/Lim	Function	Frailty	adulthood	older adulthood and retirement age
238	Kache, R., et al. (2020). "Prevalence of soil-transmitted helminth infections and associated risk factors among elderly individuals living in rural areas of southern Thailand." BMC Public Health 20(1): 1882.	59	Thailand	Not specified	ID	parasite	IP-STH	adulthood	older adulthood and retirement age

130	Chuangchaiya, S., et al. (2020). "Current prevalence of Opisthorchis viverrini infection and associated risk factors in Nakhon Phanom Province, Northeastern Thailand." Trop Biomed 37(4): 986-999.	60		Thailand	Not specified	ID	parasite	intestine parasite	adulthood	adulthood
440	Shimizu, S., et al. (2020). "Malaria cross-sectional surveys identified asymptomatic infections of Plasmodium falciparum, Plasmodium vivax and Plasmodium knowlesi in Surat Thani, a southern province of Thailand." Int J Infect Dis 96: 445-451.	61		Thailand	Rural	ID	parasite	malaria	lifespan	lifespan
497	Thinuan, P., et al. (2020). "Prevalence and Potential Predictors of Frailty among Community-Dwelling Older Persons in Northern Thailand: A Cross-Sectional Study." Int J Environ Res Public Health 17(11).	62		Thailand	Rural and urban	NCD	nutritional status	overweight/obese	adulthood	older adulthood and retirement age
						NCD	nutritional status	undernutrition	adulthood	older adulthood and retirement age
						Disability/Lim	Function	Frailty	adulthood	older adulthood and retirement age
51	Apidechkul, T. (2019). "Sexual behaviors and seroprevalence of HIV, HBV, and HCV among hill tribe youths of Northern Thailand." BMC Public Health 19(1): 1101.	63		Thailand	Not specified	ID	non_specific	STI	adulthood	reproductive age
						ID	virus	hbv	adulthood	reproductive age
						ID	virus	hcv	adulthood	reproductive age
						ID	virus	hiv	adulthood	reproductive age
288	Kubota, Y., et al. (2021). "Underweight and early childhood caries among young children in rural Cambodia: a pilot study." BDJ Open 7(1): 33.	64		Cambodia	Rural	NCD	Oral health	teeth	childhood	Pre-school child
						NCD	nutritional status	undernutrition	childhood	Pre-school child
296	Lithanatudom, P., et al. (2016). "The prevalence of alpha-thalassemia amongst Tai and Mon-Khmer ethnic groups residing in northern Thailand: A population-based study." Hematology 21(8): 480-485.	65		Thailand	Rural	NCD	blood and blood forming organ	Hemoglobinpathy	adulthood	adulthood
239	Kaewpitoon, S. J., et al. (2012). "Community-based cross-sectional study of carcinogenic human liver fluke in elderly from Surin province, Thailand." Asian Pac J Cancer Prev 13(9): 4285-4288.	66		Thailand	Not specified	ID	parasite	intestine parasite	adulthood	older adulthood and retirement age
352	P, S., et al. (2014). "High prevalence of asymptomatic malaria in south-eastern Bangladesh." Malaria journal 13.	67		Bangladesh	Not specified	ID	parasite	malaria	childhood	Child
						Symptoms	blood and blood forming organ	palpable spleen	childhood	Child
420	SKK, A., et al. (2019). "Multimorbidity and health seeking behaviours among older people in Myanmar: A community survey." PLoS One 14(7).	68		Myanmar	Rural and urban	General health			adulthood	older adulthood and retirement age
						Disability/Lim	Activity ADL	Activity ADL	adulthood	older adulthood and retirement age
						Disability/Lim	Function	Function Sensor	adulthood	older adulthood and retirement age
						SPH	srh	srh	adulthood	older adulthood and retirement age
61	B, G., et al. (2021). "Prevalence of glaucoma in the Lao People's Democratic Republic: the Vientiane Eye Study." The British journal of ophthalmology.	69		Laos	Rural and urban	NCD	cvd/circulatory	elevated blood pressure/ hypertension	adulthood	adulthood
						NCD	endocrine and metabolic	elevated glucose/ prediabetes/ diabetes	adulthood	adulthood
						Disability/Lim	Function	Function Vision	adulthood	adulthood

199	I, P., et al. (2014). "Prevalence of irritable bowel syndrome and functional dyspepsia, overlapping symptoms, and associated factors in a general population of Bangladesh." Indian journal of gastroenterology : official journal of the Indian Society of Gastroenterology 33(3).	70		Bangladesh	Not specified	NCD	digestive	Dyspepsia	adulthood	adulthood
						NCD	digestive	Irratable Bowel Syndrome	adulthood	adulthood
166	H, Y., et al. (2015). "Seroprevalence, genotypic distribution and potential risk factors of hepatitis B and C virus infections among adults in Siem Reap, Cambodia." Hepatology research : the official journal of the Japan Society of Hepatology 45(4).	71		Cambodia	Not specified	ID	virus	hbv	adulthood	adulthood
						ID	virus	hcv	adulthood	adulthood
151	E, B., et al. (2015). "Submicroscopic and asymptomatic Plasmodium falciparum and Plasmodium vivax infections are common in western Thailand - molecular and serological evidence." Malaria journal 14.	72		Thailand	Rural	ID	parasite	malaria	lifespan	lifespan
200	Imwong, M., et al. (2015). "The epidemiology of subclinical malaria infections in South-East Asia: findings from cross-sectional surveys in Thailand-Myanmar border areas, Cambodia, and Vietnam." Malar J 14: 381.	73		Cambodia M	Rural	NCD	blood and blood forming organ	Anemia	lifespan	lifespan
						ID	parasite	malaria	lifespan	lifespan
298	Lopez, J. R., et al. (2014). "Environmental exposures, lung function, and respiratory health in rural Lao PDR." Southeast Asian J Trop Med Public Health 45(1): 198-206.	74		Laos	Rural	Symptoms	respiratory	cough	adulthood	adulthood
						Symptoms	respiratory	impaired lung function	adulthood	adulthood
515	Whitfield, K. C., et al. (2015). "Poor thiamin and riboflavin status is common among women of childbearing age in rural and urban Cambodia." J Nutr 145(3): 628-633.	75		Cambodia	Rural and urban	NCD	nutritional status	micronutrition deficiency	adulthood	reproductive age
540	Zhao, A., et al. (2015). "Potential Contribution of Iron Deficiency and Multiple Factors to Anemia Among 6- to 72-Month-Old Children in the Kokang Area of Myanmar." Am J Trop Med Hyg 93(4): 836-840.	76		Myanmar	Not specified	NCD	blood and blood forming organ	Anemia	childhood	Pre-school child
						NCD	child development	small head circumference	childhood	Pre-school child
						NCD	nutritional status	undernutrition	childhood	Pre-school child
						ID	parasite	IP-STH (Ascaris spp.)	childhood	Pre-school child
						ID	virus	measles	childhood	Pre-school child
						Symptoms	digestive	diarrhea	childhood	Pre-school child
						Symptoms	multiple/non-specific	fever	childhood	Pre-school child
506	Vongphoumy, I., et al. (2015). "Snakebites in Two Rural Districts in Lao PDR: Community-Based Surveys Disclose High Incidence of an Invisible Public Health Problem." PLoS Negl Trop Dis 9(6): e0003887.	77		Laos	Not specified	NCD	Injury	Animal/insect	lifespan	lifespan
449	Sluydts, V., et al. (2014). "Spatial clustering and risk factors of malaria infections in Ratanakiri Province, Cambodia." Malar J 13: 387.	78		Cambodia	Rural	ID	parasite	malaria	lifespan	lifespan
299	Lorent, N., et al. (2014). "Community-based active tuberculosis case finding in poor urban settlements of Phnom Penh, Cambodia: a feasible and effective strategy." PLoS One 9(3): e92754.	79		Cambodia	Urban	ID	bacteria	TB	adulthood	adulthood
293	Laymanivong, S., et al. (2014). "Current status of human hookworm infections, ascariasis, trichuriasis, schistosomiasis mekongi and other trematodiases in Lao People's Democratic Republic." Am J Trop Med Hyg 90(4): 667-669.	80		Laos	Rural and urban	ID	parasite	intestine parasite	Missing	Missing

18	Ahmed, A. M., et al. (2012). "Determinants of undernutrition in children under 2 years of age from rural Bangladesh." Indian Pediatr 49(10): 821-824.	81		Bangladesh	Rural	NCD	nutritional status	undernutrition	childhood	Pre-school child
70	Banu, S., et al. (2013). "Epidemiology of tuberculosis in an urban slum of Dhaka City, Bangladesh." PLoS One 8(10): e77721.	82		Bangladesh	Urban	ID	bacteria	TB	lifespan	lifespan
202	Inpankaew, T., et al. (2014). "High prevalence of Ancylostoma ceylanicum hookworm infections in humans, Cambodia, 2012." Emerg Infect Dis 20(6): 976-982.	83		Cambodia	Rural	ID	parasite	IP-STH (hookworm)	lifespan	lifespan
422	Saengsawang, P., et al. (2013). "Infection with Opisthorchis viverrini and use of praziquantel among a working-age population in northeast Thailand." Asian Pac J Cancer Prev 14(5): 2963-2966.	84		Thailand	Rural	ID	parasite	IP-trematodes (O. viverrini)	adulthood	reproductive age and older adulthood
349	Oddo, V. M., et al. (2012). "Predictors of maternal and child double burden of malnutrition in rural Indonesia and Bangladesh." The American journal of clinical nutrition 95(4): 951-958.	85		Bangladesh	Natioanl representative	NCD	nutritional status	overweight/obese	childhood	Pre-school child
						NCD	nutritional status	undernutrition	childhood	Pre-school child
						Symptoms	digestive	diarrhea	childhood	Pre-school child
105	Chailurkit, L. O., et al. (2011). "Vitamin D status and bone health in healthy Thai elderly women." Nutrition 27(2): 160-164.	86		Thailand	Urban	NCD	nutritional status	micronutrition deficiency	adulthood	older adulthood and retirement age
525	Xeuatvongsa, A., et al. (2014). "Chronic hepatitis B prevalence among children and mothers: results from a nationwide, population-based survey in Lao People's Democratic Republic." PLoS One 9(2): e88829.	87		Laos	Not specified	ID	virus	hbv	adulthood	Reproductive age
155	Fatema, K., et al. (2015). "Application of two versions of the WHO/international society of hypertension absolute cardiovascular risk assessment tools in a rural Bangladeshi population." BMJ Open 5(10): e008140.	88		Bangladesh	Rural	NCD	cvd/circulatory	elevated blood pressure/ hypertension	adulthood	adulthood
						NCD	cvd/circulatory	non-specific	adulthood	adulthood
						NCD	endocrine and metabolic	elevated cholesterol/ hyperlipidemia	adulthood	adulthood
						NCD	endocrine and metabolic	elevated glucose/ prediabetes/ diabetes	adulthood	adulthood
183	Hiscox, A., et al. (2010). "Short report: Serological investigations of flavivirus prevalence in Khammouane Province, Lao People's Democratic Republic, 2007-2008." American Journal of Tropical Medicine and Hygiene 83(5): 1166-1169.	89		Laos	Rural	ID	virus	dengue	lifespan	lifespan
						ID	virus	je	lifespan	lifespan
275	Koly, K. N., et al. (2021). "Health-Related Quality of Life among Rural-Urban Migrants Living in Dhaka Slums: A Cross-Sectional Survey in Bangladesh." International journal of environmental research and public health 18(19).	90		Bangladesh	Urban	SPH	qol	qol	adulthood	adulthood
346	Nishino, K., et al. (2021). "Social capital and health-related quality of life of older adults living in thai rural areas." Universal Journal of Public Health 9(5): 306-316.	91		Thailand	Not specified	General health			adulthood	older adulthood and retirement age
						SPH	qol	qol	adulthood	older adulthood and retirement age
95	Boulom, S., et al. (2020). "Factors associated with child malnutrition in mountainous ethnic minority communities in Lao PDR." Glob Health Action 13(sup2): 1785736.	92		Laos	Rural	NCD	nutritional status	undernutrition	childhood	Pre-school child
						ID	parasite	malaria	childhood	Pre-school child
						Symptoms	digestive	diarrhea	childhood	Pre-school child

						Symptoms	multiple/non-specific	fever	childhood	Pre-school child
35	Alam, S., et al. (2018). "Prevalence and risk factors of non-alcoholic fatty liver disease in Bangladesh." JGH Open 2(2): 39-46.	93		Bangladesh	Rural and urban	NCD	cvd/circulatory	elevated blood pressure/ hypertension	Missing	Missing
						NCD	digestive	NAFLD	Missing	Missing
						NCD	endocrine and metabolic	elevated glucose/ prediabetes/ diabetes	Missing	Missing
						ID	virus	hbv	Missing	Missing
344	Nguyen, P. H., et al. (2017). "The nutrition and health risks faced by pregnant adolescents: Insights from a cross-sectional study in Bangladesh." PLoS One 12(6): e0178878.	94		Bangladesh	Rural	NCD	nutritional status	undernutrition	Missing	Missing
						NCD	pregnancy, childbirth and the puerperium	postpartum	Missing	Missing
496	Thiamwong, L. and J. Suwanno (2017). "Fear of Falling and Related Factors in a Community-based Study of People 60 Years and Older in Thailand." International Journal of Gerontology 11(2): 80-84.	95		Thailand	Rural	NCD	Injury	Fall	adulthood	older adulthood and retirement age
						NCD	cvd/circulatory	Stroke	adulthood	older adulthood and retirement age
						NCD	cvd/circulatory	elevated blood pressure/ hypertension	adulthood	older adulthood and retirement age
						NCD	neurology	parkinson's disease	adulthood	older adulthood and retirement age
						Disability/Lim	Activity ADL	Activity ADL	adulthood	older adulthood and retirement age
						Disability/Lim	Function &Activity	Function &Activity	adulthood	older adulthood and retirement age
						SPH	srh	srh	adulthood	older adulthood and retirement age
23	Akiyama, T., et al. (2016). "Asymptomatic malaria, growth status, and anaemia among children in Lao People's Democratic Republic: a cross-sectional study." Malar J 15(1): 499.	96		Laos	Rural	NCD	blood and blood forming organ	Anemia	childhood	Child
						NCD	nutritional status	undernutrition	childhood	Child
						ID	parasite	malaria	childhood	Child
476	Swaddiwudhipong, W., et al. (2015). "HUMAN HEALTH EFFECTS FROM CADMIUM EXPOSURE: COMPARISON BETWEEN PERSONS LIVING IN CADMIUM-CONTAMINATED AND NON-CONTAMINATED AREAS IN NORTHWESTERN THAILAND." Southeast Asian Journal of Tropical Medicine and Public Health 46(1): 133-142.	97		Thailand	Rural	NCD	cvd/circulatory	elevated blood pressure/ hypertension	adulthood	adulthood
						NCD	endocrine and metabolic	elevated cholesterol/ hyperlipidemia	adulthood	adulthood
						NCD	endocrine and metabolic	elevated glucose/ prediabetes/ diabetes	adulthood	adulthood
						NCD	genitourinary	urinary stone	adulthood	adulthood
						NCD	musculoskeleton	osteoporosis	adulthood	adulthood
163	Gruebner, O., et al. (2012). "Mental health in the slums of Dhaka - a geoepidemiological study." BMC Public Health 12: 177.	98		Bangladesh	Urban	NCD	psycological/ mental	wellbeing	adulthood	adulthood
						General health			adulthood	adulthood
						SPH	srh	srh	adulthood	adulthood

278	Kounnavong, S., et al. (2011). "Anemia and Related Factors in Preschool Children in the Southern Rural Lao People's Democratic Republic." Trop Med Health 39(4): 95-103.	99		Laos	Rural	NCD	blood and blood forming organ	Anemia	childhood	Pre-school child
						NCD	nutritional status	undernutrition	childhood	Pre-school child
						Symptoms	digestive	diarrhea	childhood	Pre-school child
						Symptoms	respiratory	cough	childhood	Pre-school child
387	Rahman, M. M., et al. (2021). "Assessing the psychological condition among general people of Bangladesh during COVID-19 pandemic." Journal of Human Behavior in the Social Environment 31(1-4): 449-463.	100		Bangladesh	Urban	NCD	psycological/ mental	anxiety	adulthood	adulthood
						NCD	psycological/ mental	depression	adulthood	adulthood
						NCD	psycological/ mental	stress	adulthood	adulthood
198	I, M., et al. (2018). "Children living in the slums of Bangladesh face risks from unsafe food and water and stunted growth is common." Acta paediatrica (Oslo, Norway : 1992) 107(7): 1230-1239.	101		Bangladesh	Urban	NCD	nutritional status	undernutrition	childhood	Pre-school child
542	แสงเงิน, เ. and ถ. มาตัน (2018). "Comparisons of Quality of Life among the Elderly in Urban, Semi-urban and Rural Communities in Sukhothai Province, Thailand." https://he02.tci-thaijo.org/index.php/jph.	102		Thailand	Rural and urban	SPH	qol	qol	adulthood	older adulthood and retirement age
415	Rojpaisarnkit, K. (2019). "Well-being of the elderly living in urban and rural areas of Thailand." The Public Health Journal of Burapha University 13(1): 113-127.	103		Thailand	Rural and urban	NCD	psycological/ mental	stress	adulthood	older adulthood and retirement age
						Disability/Lim	Activity ADL	Activity ADL	adulthood	older adulthood and retirement age
93	Boondit, J., et al. (2020). "An Epidemiological Survey of Opisthorchis viverrini Infection in a Lightly Infected Community, Eastern Thailand." American Journal of Tropical Medicine and Hygiene 102(4): 838-843.	104		Thailand	Rural and urban	ID	parasite	IP-trematodes (O. viverrini)	lifespan	lifespan
						ID	parasite	intestine parasite	lifespan	lifespan
4	Abdul Wadood, M., et al. (2020). "Bipolar disorder among married women in Bangladesh: Survey in Rajshahi city." PLoS One 15(2): e0229539.	105		Bangladesh	Urban	NCD	psycological/ mental	Psychosis	Missing	Missing
						NCD	psycological/ mental	anxiety	Missing	Missing
						NCD	psycological/ mental	stress	Missing	Missing
						General health			Missing	Missing
211	Islam, F., et al. (2020). "Prevalence and associated risk factors of general and abdominal obesity in rural and urban women in Bangladesh." PLoS One 15(5): e0233754.	106		Bangladesh	Rural and urban	NCD	nutritional status	overweight/obese	adulthood	adulthood
						NCD	nutritional status	undernutrition	adulthood	adulthood
318	Mohammad, Q. D., et al. (2020). "Prevalence of epilepsy in Bangladesh: Results from a national household survey." Epilepsia Open 5(4): 526-536.	107		Bangladesh	Natioanl representative	NCD	neurology	Epilepsy/seizure	childhood	Pre-school child
98	Bunthupanich, R., et al. (2020).	108		Thailand	Rural	NCD	blood and blood forming organ	Hemoglobinpathy	adulthood	adulthood
80	Bhuiyan, M. S. I., et al. (2020). "Prevalence of psoriasis in Bangladesh: A community based survey." Journal of Pakistan Association of Dermatologists 30(1): 39-45.	109		Bangladesh	Natioanl representative	NCD	skin and subcutaneous	psoriasis,	lifespan	lifespan
478	Sznajder, K. K., et al. (2021). "Labor migration is associated with lower rates of underweight and higher rates of obesity among left-behind wives in rural Bangladesh: a cross-sectional study." Globalization and health 17(1): 81.	110		Bangladesh	Urban	NCD	blood and blood forming organ	Anemia	adulthood	reproductive age and older adulthood

						NCD	cvd/circulatory	elevated blood pressure/ hypertension	adulthood	reproductive age and older adulthood
						NCD	endocrine and metabolic	elevated glucose/ prediabetes/ diabetes	adulthood	reproductive age and older adulthood
						NCD	nutritional status	micronutrition deficiency	adulthood	reproductive age and older adulthood
						NCD	nutritional status	overweight/obese	adulthood	reproductive age and older adulthood
389	Rahman, M. M., et al. (2021). "Functional Dyspepsia, Peptic Ulcer, and Helicobacter pylori Infection in a Rural Community of South Asia: An Endoscopy-Assisted Household Survey." Clinical and translational gastroenterology 12(4): e00334.	111		Bangladesh	Rural	NCD	digestive	Dyspepsia	adulthood	adulthood
						NCD	digestive	gastric ulsers	adulthood	adulthood
						NCD	psycological/ mental	depression	adulthood	adulthood
						NCD	psycological/ mental	stress	adulthood	adulthood
						ID	bacteria	H. pylori	adulthood	adulthood
						ID	non_specific	Gastroenteritis	adulthood	adulthood
303	Lynch, E., et al. (2021). "Hepatitis C viraemic and seroprevalence and risk factors for positivity in Northwest Cambodia: a household cross-sectional serosurvey." BMC Infectious Diseases 21(1): 223.	112		Cambodia	Not specified	ID	virus	hcv	adulthood	adulthood
536	Zaman, M. M., et al. (2021). "Estimated total cardiovascular risk in a rural area of Bangladesh: A household level cross-sectional survey done by local community health workers." BMJ Open 11(8): e046195.	113		Bangladesh	Rural	NCD	cvd/circulatory	elevated blood pressure/ hypertension	adulthood	adulthood
						NCD	endocrine and metabolic	elevated glucose/ prediabetes/ diabetes	adulthood	adulthood
						NCD	nutritional status	overweight/obese	adulthood	adulthood
161	Ghinai, I., et al. (2017). "Malaria epidemiology in central Myanmar: identification of a multi-species asymptomatic reservoir of infection." Malaria journal 16(1): 1-10.	114		Myanmar	Rural and urban	ID	parasite	malaria	lifespan	lifespan
276	Komada, K., et al. (2015). "Seroprevalence of chronic hepatitis B, as determined from dried blood spots, among children and their mothers in central Lao People's Democratic Republic: A multistage, stratified cluster sampling survey." International Journal of Infectious Diseases 36: 21-26.	115		Laos	Rural and urban	ID	virus	hbv	childhood	School-age child
371	Pothirat, C., et al. (2016). "Major Chronic Respiratory Diseases in Chiang Mai: Prevalence, Clinical Characteristics, and Their Correlations." Journal of the Medical Association of Thailand = Chotmaihet thangphaet 99(9): 1005-1013.	116		Thailand	Urban	NCD	cvd/circulatory	elevated blood pressure/ hypertension	adulthood	adulthood
						NCD	cvd/circulatory	non-specific	adulthood	adulthood
						NCD	endocrine and metabolic	elevated glucose/ prediabetes/ diabetes	adulthood	adulthood
						NCD	respiratory	COPD	adulthood	adulthood
						NCD	respiratory	asthma	adulthood	adulthood
						NCD	respiratory	chronic rhinitis	adulthood	adulthood
						ID	bacteria	TB	adulthood	adulthood
						General health			adulthood	adulthood
360	Phommasone, K., et al. (2016). "Asymptomatic Plasmodium infections in 18 villages of southern Savannakhet Province, Lao PDR (Laos)." Malaria journal 15(296).	117		Laos	Not specified	ID	parasite	malaria	adulthood	adulthood

						Symptoms	multiple/non-specific	fever	adulthood	adulthood
321	Moniruzzaman, M., et al. (2016). "Prevalence of disability in Manikganj district of Bangladesh: Results from a large-scale cross-sectional survey." BMJ Open 6(7): e010207.	118		Bangladesh	Rural and urban	Disability/Lim	Function &Activity	Function &Activity	lifespan	lifespan
237	Kabir, M. I., et al. (2016). "Climate change and health in Bangladesh: a baseline cross-sectional survey." Global Health Action 9(1): 1-N.PAG.	119		Bangladesh	Rural	NCD	Injury	Animal/insect	childhood	Pre-school child
						NCD	nutritional status	overweight/obese	Missing	Missing
						NCD	nutritional status	undernutrition	Missing	Missing
						ID	non_specific	Pneumonia	childhood	Pre-school child
						ID	parasite	malaria	childhood	Pre-school child
						ID	virus	dengue	Missing	Missing
						Symptoms	digestive	diarrhea	childhood	Pre-school child
26	Akter, F., et al. (2016). "Prevalence of, and risk factors for, symptomatic pelvic organ prolapse in Rural Bangladesh: a cross-sectional survey study." International Urogynecology Journal 27(11): 1753-1759.	120		Bangladesh	Rural	NCD	genitourinary	pelvic organ prolapse	adulthood	older adulthood and retirement age
						NCD	respiratory	COPD	adulthood	adulthood
						Symptoms	digestive	constipation	adulthood	adulthood
453	Sohn, W. M., et al. (2019). "Low-grade endemicity of opisthorchiasis, yangon, myanmar." Emerging Infectious Diseases 25(7): 1435-1437.	121		Myanmar	Urban	ID	parasite	intestine parasite	Missing	Missing
113	Chen, Y., et al. (2010). "No association between arsenic exposure from drinking water and diabetes mellitus: A cross-sectional study in Bangladesh." Environmental Health Perspectives 118(9): 1299-1305.	122		Bangladesh	Not specified	NCD	endocrine and metabolic	elevated glucose/ prediabetes/ diabetes	adulthood	adulthood
356	Pengpid, S., et al. (2019). "The prevalence, awareness, treatment, and control of hypertension among adults: the first cross-sectional national population-based survey in Laos." Vascular Health & Risk Management 15: 27-33.	123		Laos	Rural and urban	NCD	cvd/circulatory	elevated blood pressure/ hypertension	adulthood	reproductive age and older adulthood
						NCD	endocrine and metabolic	elevated cholesterol/ hyperlipidemia	adulthood	reproductive age and older adulthood
						NCD	endocrine and metabolic	elevated glucose/ prediabetes/ diabetes	adulthood	reproductive age and older adulthood
						NCD	nutritional status	overweight/obese	adulthood	reproductive age and older adulthood
						NCD	nutritional status	undernutrition	adulthood	reproductive age and older adulthood
						NCD	psycological/ mental	substance abuse	adulthood	reproductive age and older adulthood
297	Liu, Z., et al. (2019). "Geographical heterogeneity in prevalence of subclinical malaria infections at sentinel endemic sites of Myanmar." Parasites and Vectors 12(1).	124		Myanmar	Rural	ID	parasite	malaria	lifespan	lifespan
152	Ei, W. L. S. S., et al. (2019). "Nagaland health assessment: High mortality rates and difficulty accessing essential health services in Lahe Township, Republic of the Union of Myanmar." PLoS One 14(5): e0216925.	125		Myanmar	Rural and urban	General health			lifespan	lifespan
445	Siraj, S. and R. Patel (2014). "Malnutrition assessment in children under five years of age in the Meghna River islands of Narsingdi, Bangladesh." Journal of Investigative Medicine 62(2): 574.	126		Bangladesh	Rural	NCD	nutritional status	overweight/obese	childhood	Pre-school child
						NCD	nutritional status	undernutrition	childhood	Pre-school child

149	Diminic, S., et al. (2019). "The prevalence and service utilisation associated with mental and substance use disorders in Lao People's Democratic Republic: Findings from a cross-sectional survey." Epidemiology and psychiatric sciences 28(1): 54-65.	127		Laos	Rural and urban	NCD	psycological/ mental	Psychosis	childhood	Child
						NCD	psycological/ mental	anxiety	childhood	Child
						NCD	psycological/ mental	depression	childhood	Child
						NCD	psycological/ mental	ptsd	childhood	Child
						NCD	psycological/ mental	substance abuse	childhood	Child
110	Chaisiri, K., et al. (2019). "Parasitic infections in relation to practices and knowledge in a rural village in Northern Thailand with emphasis on fish-borne trematode infection." Epidemiology and Infection 147: e45.	128		Thailand	Rural	ID	parasite	intestine parasite	lifespan	lifespan
470	Suntaravitun, P. and A. Dokmaikaw (2018). "Prevalence of intestinal parasites and associated risk factors for infection among rural communities of Chachoengsao province, Thailand." Korean Journal of Parasitology 56(1): 33-39.	129		Thailand	Rural	ID	parasite	intestine parasite	lifespan	lifespan
366	Pongvongsa, T., et al. (2012). "Joint malaria surveys lead towards improved cross-border cooperation between Savannakhet province, Laos and Quang Tri province, Vietnam." Malaria journal 11: 262.	130		Laos	Rural	ID	parasite	malaria	lifespan	lifespan
432	Sattabongkot, J., et al. (2018). "Prevalence of asymptomatic Plasmodium infections with sub-microscopic parasite densities in the northwestern border of Thailand: A potential threat to malaria elimination." Malaria journal 17(1): 329.	131		Thailand	Rural	ID	parasite	malaria	lifespan	lifespan
424	Saha, U. K., et al. (2018). "Epidemiology of stroke: findings from a community-based survey in rural Bangladesh." Public Health 160: 26-32.	132		Bangladesh	Rural	NCD	cvd/circulatory	Stroke	adulthood	adulthood
						NCD	cvd/circulatory	elevated blood pressure/ hypertension	adulthood	adulthood
						NCD	endocrine and metabolic	elevated cholesterol/ hyperlipidemia	adulthood	adulthood
						NCD	endocrine and metabolic	elevated glucose/ prediabetes/ diabetes	adulthood	adulthood
						NCD	nutritional status	overweight/obese	adulthood	adulthood
						NCD	nutritional status	undernutrition	adulthood	adulthood
190	Horiuchi, Y., et al. (2019). "Urban-Rural Differences in Nutritional Status and Dietary Intakes of School-Aged Children in Cambodia." Nutrients 11(1): 14.	133		Cambodia	Natioanl representative	NCD	nutritional status	overweight/obese	childhood	School-age child
						NCD	nutritional status	undernutrition	childhood	School-age child
159	Fottrell, E., et al. (2018). "Diabetes knowledge and care practices among adults in rural Bangladesh: a cross-sectional survey." BMJ Global Health 3(4): e000891.	134		Bangladesh	Rural	NCD	endocrine and metabolic	elevated glucose/ prediabetes/ diabetes	adulthood	adulthood
332	Nahar, Q., et al. (2014). "Genital human papillomavirus infection among women in Bangladesh: findings from a population-based survey." PLoS One 9(10): e107675.	135		Bangladesh	Rural and urban	ID	virus	hpv	lifespan	lifespan
538	Zaw, M. T., et al. (2017). "Asymptomatic and sub-microscopic malaria infection in Kayah State, eastern Myanmar." Malaria journal 16(1).	136		Myanmar	Rural	ID	parasite	malaria	childhood	Pre-school child
537	Zaw, K. K., et al. (2017). "Prevalence of cardiovascular morbidities in Myanmar." BMC research notes 10(1): 99.	137		Myanmar	Rural and urban	NCD	cvd/circulatory	Stroke	adulthood	adulthood
						NCD	cvd/circulatory	angina pectoris	adulthood	adulthood

						NCD	cvd/circulatory	heart failure	adulthood	adulthood
						NCD	cvd/circulatory	myocardial infarction	adulthood	adulthood
502	Uddin, M. A., et al. (2017). "Factors related to quality of life among older adults in Bangladesh: A cross sectional survey." Nursing & Health Sciences 19(4): 518-524.	138		Bangladesh	Rural	NCD	psycological/ mental	depression	adulthood	older adulthood and retirement age
						General health			adulthood	older adulthood and retirement age
						Disability/Lim	Function	Function Non-specific	adulthood	older adulthood and retirement age
						Disability/Lim	Function	Function Sleep	adulthood	older adulthood and retirement age
						SPH	qol	qol	adulthood	older adulthood and retirement age
527	Yimnoi, P., et al. (2016). "A molecular epidemiological study of the hepatitis B virus in Thailand after 22 years of universal immunization." Journal of Medical Virology 88(4): 664-673.	139		Thailand	Not specified	ID	virus	hbv	lifespan	lifespan
233	Jirapramukpitak, T., et al. (2011). "Family violence and its 'adversity package': a community survey of family violence and adverse mental outcomes among young people." Social psychiatry and psychiatric epidemiology 46(9): 825-831.	140		Thailand	Urban	NCD	psycological/ mental	general	adulthood	reproductive age
326	Muhit, M., et al. (2016). "Generating Evidence for Program Planning: Rapid Assessment of Avoidable Blindness in Bangladesh." Ophthalmic Epidemiology 23(3): 176-184.	141		Bangladesh	Natioanl representative	NCD	eye	bilateral aphakia	adulthood	older adulthood and retirement age
						NCD	eye	unilateral aphakia	adulthood	older adulthood and retirement age
						Disability/Lim	Function	Function Vision	adulthood	older adulthood and retirement age
241	Kaewpitoon, S. J., et al. (2015). "A Cross-Sectional Survey of Intestinal Helminthiases in Rural Communities of Nakhon Ratchasima Province, Thailand." J Med Assoc Thai 98 Suppl 4: S27-32.	142		Thailand	Rural	ID	parasite	intestine parasite	Missing	Missing
330	Mya, K. S., et al. (2019). "Feeding practices and nutritional status of children age 6-23 months in Myanmar: A secondary analysis of the 2015-16 Demographic and Health Survey." PLoS One 14(1).	143		Myanmar	Natioanl representative	NCD	blood and blood forming organ	Anemia	adulthood	reproductive age
						NCD	nutritional status	undernutrition	childhood	Pre-school child
						Symptoms	digestive	diarrhea	childhood	Pre-school child
						Symptoms	multiple/non-specific	fever	childhood	Pre-school child
413	Roca-Feltrer, A., et al. (2014). "Field trial evaluation of the performances of point-of-care tests for screening G6PD deficiency in Cambodia." PLoS One 9(12): e116143.	144		Cambodia	Rural	NCD	blood and blood forming organ	G6PD deficiency	adulthood	adulthood
289	Kulaphisit, M., et al. (2017). "A comprehensive ethnic-based analysis of alpha thalassaemia allelle frequency in northern Thailand." Sci Rep 7(1): 4690.	145		Thailand	Rural	NCD	blood and blood forming organ	Hemoglobinpathy	adulthood	adulthood
60	Aye Aye, L., et al. (2017). "Sero-prevalence of hepatitis B and C viral infections in Myanmar: national and regional survey in 2015." Myanmar Health Sciences Research Journal 29(3): 167-175.	146		Myanmar	Natioanl representative	ID	virus	hbv	adulthood	adulthood
						ID	virus	hcv	adulthood	adulthood

367	Pongvongsa, T., et al. (2016). "Household clustering of asymptomatic malaria infections in Xepon district, Savannakhet province, Lao PDR." Malar J 15(1): 508.	147		Laos	Rural	ID	parasite	malaria	lifespan	lifespan
						Symptoms	multiple/non-specific	fever	lifespan	lifespan
418	Ruankham, W., et al. (2014). "Prevalence of helminthic infections and risk factors in villagers of Nanglae Sub-District, Chiang Rai Province, Thailand." J Med Assoc Thai 97 Suppl 4: S29-35.	148		Thailand	Not specified	ID	parasite	intestine parasite	lifespan	lifespan
77	Bhowmik, B., et al. (2013). "HbA(1c) as a diagnostic tool for diabetes and pre-diabetes: the Bangladesh experience." Diabet Med 30(3): e70-77.	149		Bangladesh	Rural	NCD	cvd/circulatory	elevated blood pressure/ hypertension	adulthood	adulthood
						NCD	endocrine and metabolic	elevated cholesterol/ hyperlipidemia	adulthood	adulthood
						NCD	endocrine and metabolic	elevated glucose/ prediabetes/ diabetes	adulthood	adulthood
						NCD	nutritional status	overweight/obese	adulthood	adulthood
						NCD	nutritional status	undernutrition	adulthood	adulthood
40	Alim, M. A., et al. (2014). "Respiratory involvements among women exposed to the smoke of traditional biomass fuel and gas fuel in a district of Bangladesh." Environ Health Prev Med 19(2): 126-134.	150		Bangladesh	Rural and urban	NCD	respiratory	asthma	adulthood	adulthood
						NCD	respiratory	chronic bronchitis	adulthood	adulthood
						NCD	respiratory	nasal allergy	adulthood	adulthood
						Symptoms	respiratory	wheezing	adulthood	adulthood
437	Semmarath, W., et al. (2019). "The Association between Frailty Indicators and Blood-Based Biomarkers in Early-Old Community Dwellers of Thailand." Int J Environ Res Public Health 16(18).	151		Thailand	Not specified	NCD	cvd/circulatory	myocardial infarction	adulthood	older adulthood and retirement age
						NCD	endocrine and metabolic	elevated cholesterol/ hyperlipidemia	adulthood	older adulthood and retirement age
						NCD	endocrine and metabolic	elevated glucose/ prediabetes/ diabetes	adulthood	older adulthood and retirement age
						NCD	musculoskeleton	Arthritis	adulthood	older adulthood and retirement age
						NCD	musculoskeleton	osteoporosis	adulthood	older adulthood and retirement age
						NCD	psycological/ mental	depression	adulthood	older adulthood and retirement age
						Disability/Lim	Function	Frailty	adulthood	older adulthood and retirement age
						Disability/Lim	Function	Function Cognition	adulthood	older adulthood and retirement age
480	Taburee, W., et al. (2020). "Health-Related Problems and Drivers of Health-Related Quality of Life Among Community-Dwelling Older Adults." J Prim Care Community Health 11: 2150132720913724.	152		Thailand	Rural	General health			adulthood	older adulthood and retirement age
						SPH	qol	qol	adulthood	older adulthood and retirement age
301	Lover, A. A., et al. (2018). "Prevalence and risk factors for asymptomatic malaria and genotyping of glucose 6-phosphate (G6PD) deficiencies in a vivax-predominant setting, Lao PDR: implications for sub-national elimination goals." Malar J 17(1): 218.	153		Laos	Rural	NCD	blood and blood forming organ	G6PD deficiency	childhood	Pre-school child

						ID	parasite	malaria	childhood	Pre-school child
						Symptoms	multiple/non-specific	fever	childhood	Pre-school child
406	Reinbott, A., et al. (2016). "Role of Breastfeeding and Complementary Food on Hemoglobin and Ferritin Levels in a Cambodian Cross-Sectional Sample of Children Aged 3 to 24 Months." PLoS One 11(3): e0150750.	154		Cambodia	Rural	NCD	blood and blood forming organ	Anemia	childhood	Pre-school child
						NCD	nutritional status	micronutrition deficiency	childhood	Pre-school child
290	Kyaw, W. T., et al. (2021). "Transdisciplinary online health assessment of an artisanal and small-scale gold mining community during the covid-19 pandemic in the mandalay region of Myanmar." International journal of environmental research and public health 18(21): 11206.	155		Myanmar	Rural	NCD	Injury	Poinson	Missing	Missing
						General health			Missing	Missing
455	Sperber, A. D., et al. (2021). "Worldwide Prevalence and Burden of Functional Gastrointestinal Disorders, Results of Rome Foundation Global Study." Gastroenterology 160(1): 99-114 e113.	156		Bangladesh	Not specified	NCD	digestive	Dyspepsia	adulthood	adulthood
						NCD	digestive	Irratable Bowel Syndrome	adulthood	adulthood
						NCD	digestive	functional bloating/distention	adulthood	adulthood
						NCD	digestive	functional constipation	adulthood	adulthood
						NCD	digestive	functional diarrhea	adulthood	adulthood
						SPH	qol	qol	adulthood	adulthood
156	Ferdushi, K. F., et al. (2020). Factors Associated with Coronary Heart Disease among Elderly People in Different Communities. Statistics for Data Science and Policy Analysis: 207-219.	157		Bangladesh	Rural and urban	NCD	cvd/circulatory	coronary heart disease	adulthood	older adulthood and retirement age
						NCD	cvd/circulatory	elevated blood pressure/ hypertension	adulthood	older adulthood and retirement age
						NCD	endocrine and metabolic	elevated glucose/ prediabetes/ diabetes	adulthood	older adulthood and retirement age
						NCD	nutritional status	overweight/obese	adulthood	older adulthood and retirement age
						NCD	psycological/ mental	depression	adulthood	older adulthood and retirement age
						General health			adulthood	older adulthood and retirement age
6	Adams, A. M., et al. (2020). "Healthcare seeking for chronic illness among adult slum dwellers in Bangladesh: A descriptive cross-sectional study in two urban settings." PLoS One 15(6): e0233635.	158		Bangladesh	Urban	General health			adulthood	reproductive age and older adulthood
295	Lithanatudom, P., et al. (2016). "Hemoglobin E Prevalence among Ethnic Groups Residing in Malaria-Endemic Areas of Northern Thailand and Its Lack of Association with Plasmodium falciparum Invasion In Vitro." PLoS One 11(1): e0148079.	159		Thailand	Rural	NCD	blood and blood forming organ	G6PD deficiency	adulthood	adulthood
						NCD	blood and blood forming organ	Hemoglobinpathy	adulthood	adulthood
						ID	parasite	malaria	adulthood	adulthood

111	Chandra Banik P, M. Z. M., Ahmed J and Reza Choudhury S (2017). "Prevalence of Behavioral Risk Factors of Noncommunicable Diseases in a Rural Population of Bangladesh." Austin Journal of Public Health and Epidemiology.	160		Bangladesh	Rural	NCD	cvd/circulatory	elevated blood pressure/ hypertension	adulthood	adulthood
						NCD	endocrine and metabolic	elevated glucose/ prediabetes/ diabetes	adulthood	adulthood
19	Ahmed, J., et al. (2017). "Prevalence of risk factors of non-communicable diseases in a rural area of Bangladesh." https://www.banglajol.info/index.php/CARDIO.	161		Bangladesh	Rural	NCD	cvd/circulatory	elevated blood pressure/ hypertension	adulthood	adulthood
						NCD	nutritional status	overweight/obese	adulthood	adulthood
						NCD	nutritional status	undernutrition	adulthood	adulthood
414	Roesler, A. L., et al. (2019). "Stunting, dietary diversity and household food insecurity among children under 5 years in ethnic communities of northern Thailand." Journal of Public Health 41(4): 772-780.	162		Thailand	Rural	NCD	nutritional status	undernutrition	childhood	Pre-school child
380	Rahim, M. A., et al. (2010). "Impaired fasting glucose and impaired glucose tolerance in rural population of Bangladesh." Bangladesh Med Res Counc Bull 36(2): 47-51.	163		Bangladesh	Rural	NCD	endocrine and metabolic	elevated glucose/ prediabetes/ diabetes	adulthood	adulthood
532	Zaman, K., et al. (2012). "Prevalence of smear-positive tuberculosis in persons aged >/= 15 years in Bangladesh: results from a national survey, 2007-2009." Epidemiol Infect 140(6): 1018-1027.	164		Bangladesh	Rural and urban	ID	bacteria	TB	adulthood	adulthood
539	Zhao, A., et al. (2012). "Prevalence of anemia and its risk factors among children 6-36 months old in Burma." Am J Trop Med Hyg 87(2): 306-311.	165		Myanmar	Rural	NCD	blood and blood forming organ	Anemia	childhood	Pre-school child
						NCD	nutritional status	overweight/obese	childhood	Pre-school child
						NCD	nutritional status	undernutrition	childhood	Pre-school child
						Symptoms	digestive	diarrhea	childhood	Pre-school child
						Symptoms	multiple/non-specific	fever	childhood	Pre-school child
7	Adisakwattana, P., et al. (2020). "Clinical helminthiases in Thailand border regions show elevated prevalence levels using qPCR diagnostics combined with traditional microscopic methods." Parasit Vectors 13(1): 416.	166		Thailand	Rural and urban	ID	parasite	intestine parasite	lifespan	lifespan
345	Niamnuy, N., et al. (2016). "Prevalence and Associated Risk Factors of Intestinal Parasites in Humans and Domestic Animals across Borders of Thailand and Lao Pdr: Focus on Hookworm and Threadworm." Southeast Asian J Trop Med Public Health 47(5): 901-911.	167		Laos Thailan	Rural	ID	parasite	intestine parasite	Missing	Missing
368	Pongvongsa, T., et al. (2018). "Human infection with Plasmodium knowlesi on the Laos-Vietnam border." Trop Med Health 46(1): 33.	168		Laos	Rural	ID	parasite	malaria	lifespan	lifespan
225	Janmohamed, A., et al. (2016). "Improved Sanitation Facilities are Associated with Higher Body Mass Index and Higher Hemoglobin Concentration Among Rural Cambodian Women in the First Trimester of Pregnancy." Am J Trop Med Hyg 95(5): 1211-1215.	169		Cambodia	Rural	NCD	blood and blood forming organ	Anemia	Missing	Missing
						NCD	nutritional status	overweight/obese	Missing	Missing
						NCD	nutritional status	undernutrition	Missing	Missing
53	Ashraf, H., et al. (2010). "Prevalence and risk factors of hepatitis B and C virus infections in an impoverished urban community in Dhaka, Bangladesh." BMC Infect Dis 10: 208.	170		Bangladesh	Urban	ID	virus	hbv	lifespan	lifespan
						ID	virus	hcv	lifespan	lifespan

401	Rasul, F. B., et al. (2019). "Determinants of health seeking behavior for chronic non-communicable diseases and related out-of-pocket expenditure: results from a cross-sectional survey in northern Bangladesh." J Health Popul Nutr 38(1): 48.	171	Bangladesh	Rural and urban	General health			adulthood	adulthood
419	Rusmevichientong, P., et al. (2021). "Dietary Salt-Related Determinants of Hypertension in Rural Northern Thailand." Int J Environ Res Public Health 18(2).	172	Thailand	Rural	NCD	cvd/circulatory	elevated blood pressure/ hypertension	childhood	Child
					NCD	nutritional status	overweight/obese	childhood	Child
					NCD	nutritional status	undernutrition	childhood	Child
428	Sandfort, M., et al. (2020). "Forest malaria in Cambodia: the occupational and spatial clustering of Plasmodium vivax and Plasmodium falciparum infection risk in a cross-sectional survey in Mondulkiri province, Cambodia." Malar J 19(1): 413.	173	Cambodia	Rural	ID	parasite	malaria	lifespan	lifespan
335	Naorungroj, S. and S. Thitasomakul (2020). "Negative impacts of self-reported five-year incident tooth loss and number of teeth on oral health-related quality of life." Journal of International Oral Health 12(7): 5-12.	174	Thailand	Not specified	NCD	Oral health	teeth	adulthood	reproductive age and older adulthood
					NCD	cvd/circulatory	elevated blood pressure/ hypertension	adulthood	reproductive age and older adulthood
					NCD	endocrine and metabolic	elevated glucose/ prediabetes/ diabetes	adulthood	reproductive age and older adulthood
					Disability/Lim	Activity	Activity Oral-impact	adulthood	reproductive age and older adulthood
203	Ir, P., et al. (2010). "Self-reported serious illnesses in rural Cambodia: a cross-sectional survey." PLoS One 5(6): e10930.	175	Cambodia	Rural	General health			lifespan	lifespan
250	Khalequzzaman, M., et al. (2017). "Prevalence of non-communicable disease risk factors among poor shantytown residents in Dhaka, Bangladesh: a community-based cross-sectional survey." BMJ Open 7(11): e014710.	176	Bangladesh	Urban	NCD	cvd/circulatory	elevated blood pressure/ hypertension	adulthood	reproductive age and older adulthood
					NCD	endocrine and metabolic	elevated cholesterol/ hyperlipidemia	adulthood	reproductive age and older adulthood
					NCD	endocrine and metabolic	elevated glucose/ prediabetes/ diabetes	adulthood	reproductive age and older adulthood
					NCD	nutritional status	overweight/obese	adulthood	reproductive age and older adulthood
					NCD	nutritional status	undernutrition	adulthood	reproductive age and older adulthood
304	Mahmood, M. A., et al. (2018). "Snakebite incidence in two townships in Mandalay Division, Myanmar." PLoS Negl Trop Dis 12(7): e0006643.	177	Myanmar	Rural	NCD	Injury	Animal/insect	lifespan	lifespan
467	Sukkriang, N. and K. Somrak (2021). "Correlation Between Mini Nutritional Assessment and Anthropometric Measurements Among Community-Dwelling Elderly Individuals in Rural Southern Thailand." J Multidiscip Healthc 14: 1509-1520.	178	Thailand	Rural	NCD	cvd/circulatory	elevated blood pressure/ hypertension	adulthood	older adulthood and retirement age
					NCD	nutritional status	overweight/obese	adulthood	older adulthood and retirement age
					NCD	nutritional status	undernutrition	adulthood	older adulthood and retirement age
					General health			adulthood	older adulthood and retirement age

272	Klongthalay, S. and K. Suriyaprom (2020). "Increased Uric Acid and Life Style Factors Associated with Metabolic Syndrome in Thais." Ethiop J Health Sci 30(2): 199-208.	179	Thailand	Urban	NCD	cvd/circulatory	elevated blood pressure/ hypertension	adulthood	adulthood
					NCD	endocrine and metabolic	Metabolic Syndrome	adulthood	adulthood
					NCD	endocrine and metabolic	elevated cholesterol/ hyperlipidemia	adulthood	adulthood
					NCD	endocrine and metabolic	elevated glucose/ prediabetes/ diabetes	adulthood	adulthood
					NCD	nutritional status	overweight/obese	adulthood	adulthood
412	Ridwanur, R., et al. (2010). "Annual incidence of snake bite in rural Bangladesh." PLoS neglected tropical diseases 4(10): e860.	180	Bangladesh	Rural	NCD	Injury	Animal/insect	lifespan	lifespan
359	Phanmany, S., et al. (2019). "Molecular prevalence of thalassemia and hemoglobinopathies among the Lao Loum Group in the Lao People's Democratic Republic." Int J Lab Hematol 41(5): 650-656.	181	Laos	Not specified	NCD	blood and blood forming organ	Hemoglobinpathy	adulthood	adulthood
402	Rattanapitoon, S. K., et al. (2020). "Prevalence and intensity of helminths among inhabitants of the Chi River and Lahanna water reservoir areas of Northeastern Thailand." Tropical Biomedicine 37(3): 730-743.	182	Thailand	Rural	ID	parasite	intestine parasite	lifespan	lifespan
69	Banik, P. C., et al. (2018). "Prevalence of Risk Factors of non-communicable Diseases in an Adult Population of Rural Bangladesh." https://www.banglajol.info/index.php/CARDIO.	183	Bangladesh	Rural	NCD	cvd/circulatory	elevated blood pressure/ hypertension	adulthood	reproductive age and older adulthood
					NCD	endocrine and metabolic	elevated cholesterol/ hyperlipidemia	adulthood	reproductive age and older adulthood
					NCD	endocrine and metabolic	elevated glucose/ prediabetes/ diabetes	adulthood	reproductive age and older adulthood
					NCD	nutritional status	overweight/obese	adulthood	reproductive age and older adulthood
					NCD	nutritional status	undernutrition	adulthood	reproductive age and older adulthood
475	Suwanprathes, P., et al. (2010). "Epidemiology of sleep-related complaints associated with sleep-disordered breathing in Bangkok, Thailand." Sleep medicine 11(10): 1025-1030.	184	Thailand	Urban	Disability/Lim	Function	Function Sleep	adulthood	adulthood
313	Mistry, S. K., et al. (2021). "Exploring depressive symptoms and its associates among Bangladeshi older adults amid COVID-19 pandemic: findings from a cross-sectional study." Soc Psychiatry Psychiatr Epidemiol 56(8): 1487-1497.	185	Bangladesh	Rural and urban	NCD	psycological/ mental	depression	adulthood	older adulthood and retirement age
					NCD	psycological/ mental	low memory or concentration	adulthood	older adulthood and retirement age
468	Sultana, M., et al. (2016). "Inequalities in Health Status from EQ-5D Findings: A Cross-Sectional Study in Low-Income Communities of Bangladesh." International journal of health policy and management 5(5): 301-308.	186	Bangladesh	Rural and urban	SPH	qol	qol	adulthood	adulthood
446	Siripongpreeda, B., et al. (2016). "High prevalence of advanced colorectal neoplasia in the Thai population: a prospective screening colonoscopy of 1,404 cases." BMC gastroenterology 16: 101.	187	Thailand	Not specified	NCD	digestive	hyperplastic polyp of colon	adulthood	older adulthood and retirement age
					NCD	digestive	other pathologies of the colon (inflammatory polyp, colitis, lipoma)	adulthood	older adulthood and retirement age

						NCD	neoplasma	cancer of colon	adulthood	older adulthood and retirement age
						NCD	neoplasma	colon adenoma	adulthood	older adulthood and retirement age
136	Cook, J., et al. (2012). "Sero-epidemiological evaluation of changes in Plasmodium falciparum and Plasmodium vivax transmission patterns over the rainy season in Cambodia." Malar J 11: 86.	188		Cambodia	Rural	ID	parasite	malaria	Missing	Missing
375	Prasitwattanaseree, S., et al. (2016). "Risk Factors of Malnutrition among Karen Children in Chiang Mai, Thailand." Open Journal of Statistics 06(05): 756-765.	189		Thailand	Not specified	NCD	blood and blood forming organ	Anemia	childhood	School-age child
						NCD	nutritional status	undernutrition	childhood	School-age child
498	Thomas, T. A., et al. (2010). "Malnutrition and helminth infection affect performance of an interferon gamma-release assay." Pediatrics 126(6): e1522-1529.	190		Bangladesh	Urban	ID	bacteria	TB	childhood	School-age child
						ID	parasite	intestine parasite	childhood	School-age child
355	Peansukwech, U., et al. (2017). "Family history of liver cancer may indicate chronic hepatitis B virus infection in an endemic area." Asian Pacific Journal of Tropical Disease 7(11): 650-652.	191		Thailand	Not specified	NCD	neoplasma	family history of liver cancer	Missing	Missing
						ID	virus	hbv	Missing	Missing
223	Jankhotkaew, J., et al. (2017). "The impact of heavy drinkers on others' health and well-being in Lao PDR and Thailand." Journal of Substance Use 22(6): 617-623.	192		Laos Thailan	Rural and urban	NCD	psycological/ mental	substance abuse	adulthood	reproductive age and older adulthood
						NCD	psycological/ mental	wellbeing	adulthood	reproductive age and older adulthood
						SPH	qol	qol	adulthood	reproductive age and older adulthood
72	Baum, E., et al. (2016). "Common asymptomatic and submicroscopic malaria infections in Western Thailand revealed in longitudinal molecular and serological studies: A challenge to malaria elimination." Malaria journal 15(1): 333.	193		Thailand	Rural	ID	parasite	malaria	lifespan	lifespan
510	Wang, R. B., et al. (2016). "Lessons on malaria control in the ethnic minority regions in Northern Myanmar along the China border, 2007-2014." Infect Dis Poverty 5(1): 95.	194		Myanmar	Rural	ID	parasite	malaria	adulthood	adulthood
530	Yoonuan, T., et al. (2010). "Molecular and serological survey on taeniasis and cysticercosis in Kanchanaburi Province, Thailand." Parasitology International 59(3): 326-330.	195		Thailand	Rural	ID	parasite	tapeworm (Taeniasis, cysticercosis)	lifespan	lifespan
224	Jankhotkaew, J., et al. (2020). "Alcohol's harm to others and subjective well-being: Cross-sectional studies in Lao People's Democratic Republic and Thailand." Drug Alcohol Rev 39(5): 546-554.	196		Laos Thailan	Natioanl representative	NCD	psycological/ mental	substance abuse	adulthood	reproductive age and older adulthood
						NCD	psycological/ mental	wellbeing	adulthood	reproductive age and older adulthood
523	Wongprachum, K., et al. (2016). "Molecular Heterogeneity of Thalassemia among Pregnant Laotian Women." Acta Haematol 135(2): 65-69.	197		Laos	Not specified	NCD	blood and blood forming organ	Hemoglobinpathy	Missing	Missing
174	Haque, U., et al. (2011). "The role of age, ethnicity and environmental factors in modulating malaria risk in Rajasthali, Bangladesh." Malar J 10: 367.	198		Bangladesh	Rural	ID	parasite	malaria	lifespan	lifespan

354	Parr, J. D., et al. (2011). "Diagnosis of chronic conditions with modifiable lifestyle risk factors in selected urban and rural areas of Bangladesh and sociodemographic variability therein." Bmc Health Services Research 11(1): 309-309.	199		Bangladesh	Rural and urban	General health			adulthood	adulthood
376	Preux, P. M., et al. (2011). "First-ever, door-to-door cross-sectional representative study in Prey Veng province (Cambodia)." Epilepsia 52(8): 1382-1387.	200		Cambodia	Rural	NCD	neurology	Epilepsy/seizure	lifespan	lifespan
74	Bhalla, D., et al. (2013). "Epilepsy in Cambodia-treatment aspects and policy implications: a population-based representative survey." PLoS One 8(9): e74817.	201		Cambodia	Rural	NCD	neurology	Epilepsy/seizure	lifespan	lifespan
427	Samnieng, P., et al. (2011). "Oral health status and chewing ability is related to mini-nutritional assessment results in an older adult population in Thailand." Journal of nutrition in gerontology and geriatrics 30(3): 291-304.	202		Thailand	Not specified	NCD	Oral health	teeth	adulthood	older adulthood and retirement age
						NCD	nutritional status	overweight/obese	adulthood	older adulthood and retirement age
						NCD	nutritional status	undernutrition	adulthood	older adulthood and retirement age
						General health			adulthood	older adulthood and retirement age
						Disability/Lim	Function	Function Ingestive	adulthood	older adulthood and retirement age
114	Cherry, N., et al. (2012). "Disability among elderly rural villagers: report of a survey from Gonoshasthaya Kendra, Bangladesh." BMC Public Health 12: 379.	203		Bangladesh	Rural	General health			adulthood	older adulthood and retirement age
						Disability/Lim	Activity ADL	Activity ADL	adulthood	older adulthood and retirement age
197	Huda, M. N., et al. (2012). "Prevalence of Chronic Kidney Disease and Its Association with Risk Factors in Disadvantageous Population." International Journal of Nephrology 2012: 1-7.	204		Bangladesh	Urban	NCD	cvd/circulatory	elevated blood pressure/ hypertension	adulthood	reproductive age and older adulthood
						NCD	endocrine and metabolic	elevated glucose/ prediabetes/ diabetes	adulthood	reproductive age and older adulthood
						NCD	genitourinary	CKD	adulthood	reproductive age and older adulthood
						NCD	nutritional status	overweight/obese	adulthood	reproductive age and older adulthood
						Symptoms	genitourinary	proteinuria	adulthood	reproductive age and older adulthood
						Symptoms	genitourinary	raised serum creatinine	adulthood	reproductive age and older adulthood
24	Akkhavong, K., et al. (2013). "Helminth infection in southern Laos: High prevalence and low awareness." Parasites and Vectors 6(1): 328.	205		Laos	Rural	ID	parasite	intestine parasite	lifespan	lifespan
58	Aung, T., et al. (2013). "Incidence of pediatric diarrhea and public-private preferences for treatment in rural Myanmar: a randomized cluster survey." J Trop Pediatr 59(1): 10-16.	206		Myanmar	Rural	Symptoms	digestive	diarrhea	childhood	Pre-school child
140	Das, S. K., et al. (2013). "Health care-seeking behavior for childhood diarrhea in Mirzapur, rural Bangladesh." The American journal of tropical medicine and hygiene 89(1): 62-68.	207		Bangladesh	Rural	Symptoms	digestive	diarrhea	childhood	Pre-school child

194	Hossain, S., et al. (2013). "Tuberculin survey in Bangladesh, 2007-2009: prevalence of tuberculous infection and implications for TB control." Int J Tuberc Lung Dis 17(10): 1267-1272.	208		Bangladesh	Natioanl representative	ID	bacteria	TB	childhood	School-age child
236	Jong-Yil, C., et al. (2013). "Hyperendemicity of Haplorchis taichui Infection among Riparian People in Saravane and Champasak Province, Lao PDR." The Korean Journal of Parasitology: 305-311.	209		Laos	Rural	ID	parasite	intestine parasite	lifespan	lifespan
258	Khan, N. Z., et al. (2013). "Validation of a home-based neurodevelopmental screening tool for under 2-year-old children in Bangladesh." Child: Care, Health and Development 39(5): 643-650.	210		Bangladesh	Urban	NCD	child development	child development	childhood	Pre-school child
308	Mayxay, M., et al. (2013). "Respiratory illness healthcare-seeking behavior assessment in the Lao People's Democratic Republic (Laos)." BMC Public Health 13: 444.	211		Laos	Rural and urban	ID	non_specific	ARI	Missing	Missing
46	Anand, S., et al. (2014). "High prevalence of chronic kidney disease in a community survey of urban Bangladeshis: a cross-sectional study." Globalization and health 10: 9.	212		Bangladesh	Urban	NCD	cvd/circulatory	elevated blood pressure/ hypertension	adulthood	adulthood
						NCD	endocrine and metabolic	Metabolic Syndrome	adulthood	adulthood
						NCD	genitourinary	CKD	adulthood	adulthood
504	Vathesatogkit, P., et al. (2012). "Effects of lifestyle factors, disease history and awareness on health-related quality of life in a Thai population." European Heart Journal 33(SUPPL. 1): 763-764.	213		Thailand	Not specified	General health			adulthood	reproductive age and older adulthood
						SPH	qol	qol	adulthood	reproductive age and older adulthood
172	Hannan, M. A., et al. (2014). "Prevalence of Gingivitis, Plaque accumulation and Decayed, Missing and Filled Teeth among slum population in Bangladesh." Bangladesh Medical Research Council bulletin 40(2): 47-51.	214		Bangladesh	Urban	NCD	Oral health	periodontal	lifespan	lifespan
204	Isipradit, S., et al. (2014). "The first rapid assessment of avoidable blindness (RAAB) in Thailand." PLoS One 9(12): e114245.	215		Thailand	Natioanl representative	Disability/Lim	Function	Function Vision	adulthood	older adulthood and retirement age
320	Mollica, R. F., et al. (2014). "The enduring mental health impact of mass violence: a community comparison study of Cambodian civilians living in Cambodia and Thailand." Int J Soc Psychiatry 60(1): 6-20.	216		Cambodia T	Rural	NCD	psycological/ mental	anxiety	adulthood	adulthood
						NCD	psycological/ mental	depression	adulthood	adulthood
						NCD	psycological/ mental	ptsd	adulthood	adulthood
316	Moe Dr, S., et al. (2012). "Health seeking behaviour of elderly in Myanmar." International Journal of Collaborative Research on Internal Medicine and Public Health 4(8): 1538-1544.	218		Myanmar	Rural	SPH	srh	srh	adulthood	older adulthood and retirement age
379	Puavilai, W., et al. (2011). "Prevalence and some important risk factors of hypertension in Ban Paew District, second report." Journal of the Medical Association of Thailand 94(9): 1069-1076.	219		Thailand	Rural	NCD	cvd/circulatory	elevated blood pressure/ hypertension	adulthood	reproductive age and older adulthood
251	Khan, A. K. (2013). "Ocular injury: Prevalence in different rural population of Bangladesh." Bangladesh Medical Research Council bulletin 39(3): 130-138.	220		Bangladesh	Rural	NCD	Injury	Eye	lifespan	lifespan
329	Murthy, G. V. S., et al. (2014). "Assessing the prevalence of sensory and motor impairments in childhood in Bangladesh using key informants." Archives of Disease in Childhood 99(12): 1103-1108.	221		Bangladesh	Rural	NCD	neurology	Epilepsy/seizure	childhood	Child
						Disability/Lim	Body structure&function	Body structure&function	childhood	Child

491	Thaewnongiew, K., et al. (2014). "Prevalence and risk factors for Opisthorchis viverrini infections in upper Northeast Thailand." Asian Pacific Journal of Cancer Prevention 15(16): 6609-6612.	222		Thailand	Rural	ID	parasite	intestine parasite	adulthood	adulthood
143	Davis, W. W., et al. (2015). "Health and Human Rights in Karen State, Eastern Myanmar." PLoS One 10(8): e0133822.	223		Myanmar	Rural	NCD	nutritional status	undernutrition	childhood	Pre-school child
						Symptoms	digestive	diarrhea	childhood	Pre-school child
						Disability/Lim	Function	Function Vision	adulthood	adulthood
167	Hafiz, I., et al. (2015). "Clinical case estimates of lymphatic filariasis in an endemic district of Bangladesh after a decade of mass drug administration." Trans R Soc Trop Med Hyg 109(11): 700-709.	224		Bangladesh	Rural	ID	parasite	intestine parasite	lifespan	lifespan
292	Law, I., et al. (2015). "The first national tuberculosis prevalence survey of Lao PDR (2010-2011)." Tropical Medicine and International Health 20(9): 1146-1154.	225		Laos	Natioanl representative	ID	bacteria	TB	adulthood	adulthood
370	Pothirat, C., et al. (2015). "A comparative study of COPD burden between urban vs rural communities in northern Thailand." International journal of chronic obstructive pulmonary disease 10: 1035-1042.	226		Thailand	Rural and urban	NCD	nutritional status	overweight/obese	adulthood	adulthood
						NCD	respiratory	COPD	adulthood	adulthood
535	Zaman, M. M., et al. (2015). "Prevalence of Stroke in a Rural Population of Bangladesh." Global heart 10(4): 333-334.	227		Bangladesh	Rural	NCD	cvd/circulatory	Stroke	adulthood	adulthood
205	Islam, F. M. A. (2019). "Psychological distress and its association with socio-demographic factors in a rural district in Bangladesh: A cross-sectional study." PLoS One 14(3): e0212765.	228		Bangladesh	Rural	NCD	psycological/ mental	psychological distress	adulthood	adulthood
268	Khotcharrat, R., et al. (2015). "Epidemiology of Age-Related Macular Degeneration among the Elderly Population in Thailand." Journal of the Medical Association of Thailand = Chotmaihet thangphaet 98(8): 790-797.	229		Thailand	Rural and urban	NCD	eye	age-related macular degeneration	adulthood	older adulthood and retirement age
466	Sukeepaisalncharoen, W., et al. "Who should be checked for hepatitis C virus infection in endemic areas?" Asian Pac J Trop Med 9(10): 991-994.	230		Thailand	Rural and urban	ID	virus	hcv	Missing	Missing
337	Nasreen, H. E., et al. (2011). "Prevalence and associated factors of depressive and anxiety symptoms during pregnancy: a population based study in rural Bangladesh." BMC women's health 11: 22.	331		Bangladesh	Rural	NCD	psycological/ mental	anxiety	Missing	Missing
						NCD	psycological/ mental	depression	Missing	Missing
36	Ali, D., et al. (2013). "Household food insecurity is associated with higher child undernutrition in Bangladesh, Ethiopia, and Vietnam, but the effect is not mediated by child dietary diversity." J Nutr 143(12): 2015-2021.	231	alive an	Bangladesh	Rural	NCD	blood and blood forming organ	Anemia	childhood	Pre-school child
41	International Food Policy Research Institute (IFPRI). 2020. Bangladesh Alive & Thrive Baseline Survey 2010: Shasthya Kormi (SK). Washington, DC: IFPRI [dataset]. https://doi.org/10.7910/DVN/SYENH1. Harvard Dataverse. Version 1.					NCD	nutritional status	overweight/obese	childhood	Pre-school child
423	Saha KK, Bamezai A, Khaled A, Subandoro A, Rawat R, Menon P. Alive & Thrive Baseline Survey Report: Bangladesh. Washington, D.C.: Alive & Thrive, 2011.					NCD	nutritional status	undernutrition	childhood	Pre-school child
						Symptoms	digestive	diarrhea	childhood	Pre-school child
						Symptoms	multiple/non-specific	fever	childhood	Pre-school child
						Symptoms	respiratory	cough	childhood	Pre-school child
57	Aung, M. N., et al. (2012). "Assessing awareness and knowledge of hypertension in an at-risk population in the Karen ethnic rural community, Thasongyang, Thailand." Int J Gen Med 5: 553-561.	232		Thailand	Rural	NCD	cvd/circulatory	elevated blood pressure/ hypertension	adulthood	adulthood

300	Lorga, T., et al. (2012). "Predicting prediabetes in a rural community: a survey among the Karen ethnic community, Thasongyang, Thailand." Int J Gen Med 5: 219-225.					NCD	cvd/circulatory	non-specific	adulthood	adulthood
						NCD	endocrine and metabolic	elevated glucose/ prediabetes/ diabetes	adulthood	adulthood
						NCD	nutritional status	overweight/obese	adulthood	adulthood
						NCD	nutritional status	undernutrition	adulthood	adulthood
62	B-National micronutrient survey 2011:	233	b-natior	Bangladesh	Natioanl representative	NCD	blood and blood forming organ	Anemia	adulthood	Reproductive age
						NCD	nutritional status	micronutrition deficiency	adulthood	reproductive age
						NCD	nutritional status	overweight/obese	childhood	Pre-school child
						NCD	nutritional status	undernutrition	childhood	Pre-school child
261	Khanam, M. A., et al. "Undiagnosed and uncontrolled hypertension among the adults in rural Bangladesh: findings from a community-based study." J Hypertens 33(12): 2399-2406.	234	b-steps2	Bangladesh	Rural	NCD	cvd/circulatory	elevated blood pressure/ hypertension	adulthood	reproductive age and older adulthood
263	Khanam, M. A., et al. (2015). "Prevalence and determinants of pre-hypertension and hypertension among the adults in rural Bangladesh: findings from a community-based study." BMC Public Health 15(1): 203.					NCD	nutritional status	overweight/obese	adulthood	reproductive age and older adulthood
404	Razzaque, A., et al. (2011). "Sociodemographic differentials of selected noncommunicable diseases risk factors among adults in Matlab, Bangladesh: findings from a WHO STEPS survey." Asia-Pacific journal of public health 23(2): 183-191.					NCD	nutritional status	undernutrition	adulthood	reproductive age and older adulthood
28	Akter, S., et al. (2015). "Higher circulatory level of endothelin-1 in hypertensive subjects screened through a cross-sectional study of rural Bangladeshi women." Hypertension research : official journal of the Japanese Society of Hypertension 38(3): 208-212.	235	b-steps2	Bangladesh	Natioanl representative	NCD	cvd/circulatory	elevated blood pressure/ hypertension	adulthood	adulthood
331	NCD Risk factor survey Bangladesh 2010					NCD	endocrine and metabolic	elevated glucose/ prediabetes/ diabetes	adulthood	adulthood
395	Rahman, M., et al. (2018). "Prevalence, treatment patterns, and risk factors of hypertension and pre-hypertension among Bangladeshi adults." J Hum Hypertens 32(5): 334-348.					NCD	nutritional status	overweight/obese	adulthood	adulthood
534	Zaman, M. M., et al. (2015). "Clustering of non-communicable diseases risk factors in Bangladeshi adults: An analysis of STEPS survey 2013." BMC Public Health 15: 659.	236	b-steps2	Bangladesh	Natioanl representative	NCD	cvd/circulatory	elevated blood pressure/ hypertension	adulthood	adulthood
						NCD	endocrine and metabolic	elevated glucose/ prediabetes/ diabetes	adulthood	adulthood
						NCD	nutritional status	overweight/obese	adulthood	adulthood
341	Report: National STEPS Survey for Non-communicable Disease Risk Factors in Bangladesh 2018	237	b-steps2	Bangladesh	Natioanl representative	NCD	Oral health	overall	adulthood	adulthood
411	Riaz, B. K., et al. (2020). "Risk factors for non-communicable diseases in Bangladesh: findings of the population-based cross-sectional national survey 2018." BMJ Open 10(11): e041334.					NCD	cvd/circulatory	elevated blood pressure/ hypertension	adulthood	adulthood
443	Simmons, S. S., et al. (2021). "The Influence of Anthropometric Indices and Intermediary Determinants of Hypertension in Bangladesh." Int J Environ Res Public Health 18(11).					NCD	cvd/circulatory	non-specific	adulthood	adulthood

						NCD	endocrine and metabolic	elevated cholesterol/ hyperlipidemia	adulthood	adulthood
						NCD	endocrine and metabolic	elevated glucose/ prediabetes/ diabetes	adulthood	adulthood
						NCD	nutritional status	overweight/obese	adulthood	adulthood
						NCD	nutritional status	undernutrition	adulthood	adulthood
						Symptoms	cvd/circulatory	abnormal paulse rate	adulthood	adulthood
193	Hossain, S., et al. (2012). "Socio economic position in TB prevalence and access to services: results from a population prevalence survey and a facility-based survey in Bangladesh." PLoS One 7(9): e44980.	238	b-tb200	Bangladesh	Natioanl representative	ID	bacteria	TB	childhood	School-age child
408	Report of the nationwide tuberculosis prevalence diseases-cum-infection survey (2007-2009)									
533	Zaman, K., et al. (2012). "Prevalence of smear-positive tuberculosis in persons aged >= 15 years in Bangladesh: results from a national survey, 2007-2009." Epidemiology and Infection 140(6): 1018-1027.									
325	Muangpaisan, W., et al. (2017). "Development of a Thai Parkinson's Disease Screening Tool and the Prevalence of Parkinsonism and Parkinson's Disease, Based on a Community Survey in Bangkok." Neuroepidemiology 49(1-2): 74-81.	251		Thailand	Urban	NCD	neurology	parkinson's disease	adulthood	older adulthood and retirement age
						Disability/Lim	Activity ADL	Activity ADL	adulthood	older adulthood and retirement age
439	Shawon, R. A., et al. (2019). "Alarming rise in fatal electrocutions in Bangladesh: Comparison of two national surveys." Burns 45(6): 1471-1476.	255	banglad	Bangladesh	Not specified	NCD	Injury	Electrocution	lifespan	lifespan
439	Shawon, R. A., et al. (2019). "Alarming rise in fatal electrocutions in Bangladesh: Comparison of two national surveys." Burns 45(6): 1471-1476.	254	banglad	Bangladesh	Not specified	NCD	Injury	Electrocution	lifespan	lifespan
66	Islam RM, Bell RJ, Hossain MB, Davis SR. Bangladesh Midlife Women's Health Study (BMWHS): methods, challenges and experiences. Maturitas. 2015 Jan;80(1):89-94. doi: 10.1016/j.maturitas.2014.10.005. Epub 2014 Oct 22. PMID: 25459366.	252	banglad	Bangladesh	Natioanl representative	NCD	genitourinary	Menopause	adulthood	reproductive age and older adulthood
220	Islam, R. M., et al. (2018). "Types of urinary incontinence in Bangladeshi women at midlife: Prevalence and risk factors." Maturitas 116: 18-23.					NCD	genitourinary	pelvic floor distress	adulthood	reproductive age and older adulthood
						NCD	genitourinary	urinary incontinence	adulthood	reproductive age and older adulthood
						SPH	qol	qol	adulthood	reproductive age and older adulthood
208	Islam, F. M., et al. (2016). "Factors Associated with Disability in Rural Bangladesh: Bangladesh Population-Based Diabetes and Eye Study (BPDES)." PLoS One 11(12): e0165625.	253	banglad	Bangladesh	Rural	NCD	cvd/circulatory	elevated blood pressure/ hypertension	adulthood	adulthood
209	Islam, F. M., et al. (2016). "Prediabetes, diagnosed and undiagnosed diabetes, their risk factors and association with knowledge of diabetes in rural Bangladesh: The Bangladesh Population-based Diabetes and Eye Study." J Diabetes 8(2): 260-268.					NCD	endocrine and metabolic	elevated glucose/ prediabetes/ diabetes	adulthood	adulthood
210	Islam, F. M., et al. (2016). "Undiagnosed hypertension in a rural district in Bangladesh: The Bangladesh Population-based Diabetes and Eye Study (BPDES)." J Hum Hypertens 30(4): 252-259.					Disability/Lim	Function &Activity	Function &Activity	adulthood	adulthood

362	Phuong Hong, N., et al. (2021). "Adolescent birth and child undernutrition: an analysis of demographic and health surveys in Bangladesh, 1996-2017." Annals reports. 1500: 69-81.	239	bdhs199	Bangladesh	Natioanl representative	NCD	nutritional status	undernutrition	childhood	Pre-school child
362	Phuong Hong, N., et al. (2021). "Adolescent birth and child undernutrition: an analysis of demographic and health surveys in Bangladesh, 1996-2017." Annals reports. 1500: 69-81.	240	bdhs199	Bangladesh	Natioanl representative	NCD	nutritional status	undernutrition	childhood	Pre-school child
87	Biswas, R. K., et al. (2019). "Double burden of underweight and overweight among women of reproductive age in Bangladesh." Public health nutrition 22(17): 3163-3174.	241	bdhs200	Bangladesh	Natioanl representative	NCD	nutritional status	overweight/obese	adulthood	reproductive age
362	Phuong Hong, N., et al. (2021). "Adolescent birth and child undernutrition: an analysis of demographic and health surveys in Bangladesh, 1996-2017." Annals reports. 1500: 69-81.					NCD	nutritional status	undernutrition	childhood	Pre-school child
						NCD	nutritional status	undernutrition	adulthood	reproductive age
42	Alom, J., et al. (2012). "Nutritional status of under-five children in Bangladesh: a multilevel analysis." J Biosoc Sci 44(5): 525-535.	242	bdhs200	Bangladesh	Natioanl representative	NCD	nutritional status	overweight/obese	adulthood	reproductive age
87	Biswas, R. K., et al. (2019). "Double burden of underweight and overweight among women of reproductive age in Bangladesh." Public health nutrition 22(17): 3163-3174.					NCD	nutritional status	undernutrition	childhood	Pre-school child
362	Phuong Hong, N., et al. (2021). "Adolescent birth and child undernutrition: an analysis of demographic and health surveys in Bangladesh, 1996-2017." Annals reports. 1500: 69-81.					NCD	nutritional status	undernutrition	adulthood	reproductive age
421	SM, M. K. (2011). "Socio-economic determinants of severe and moderate stunting among under-five children of rural Bangladesh." Malaysian Journal of Nutrition 17(1): 105-118.									
20	Ahmed, S., et al. (2021). "Household biomass fuel use is associated with chronic childhood malnutrition: Result from a nationwide cross-sectional survey in Bangladesh." Indoor air.	243	bdhs201	Bangladesh	Natioanl representative	NCD	blood and blood forming organ	Anemia	adulthood	reproductive age
27	Akter, S., et al. (2014). "Prevalence of diabetes and prediabetes and their risk factors among Bangladeshi adults: a nationwide survey." Bulletin of the World Health Organization 92(3): 204-213.					NCD	cvd/circulatory	elevated blood pressure/ hypertension	adulthood	adulthood
30	Al Kibria, G. M., et al. (2019). "Sex differences in prevalence and associated factors of prehypertension and hypertension among Bangladeshi adults." Int J Cardiol Hypertens 1: 100006.					NCD	endocrine and metabolic	elevated glucose/ prediabetes/ diabetes	adulthood	adulthood
31	Al Kibria, G. M., et al. (2021). "Clustering of hypertension, diabetes and overweight/obesity according to socioeconomic status among Bangladeshi adults." Journal of biosocial science 53(2): 157-166.					NCD	nutritional status	overweight/obese	adulthood	adulthood
37	Ali, M., et al. (2021). "Maternal health status and household food security on determining childhood anemia in Bangladesh -a nationwide cross-sectional study." BMC Public Health 21(1): 1581.					NCD	nutritional status	undernutrition	childhood	Child
39	Ali, N., et al. (2019). "Sex-specific prevalence, inequality and associated predictors of hypertension, diabetes, and comorbidity among Bangladeshi adults: results from a nationwide cross-sectional demographic and health survey." BMJ Open 9(9): e029364.					NCD	nutritional status	undernutrition	adulthood	adulthood
84	Bishwajit, G., et al. (2017). "Diabetes mellitus and high blood pressure in relation to BMI among adult non-pregnant women in Bangladesh." Diabetes Metab Syndr 11 Suppl 1: S217-S221.					ID	non_specific	ARI	childhood	Pre-school child

84	Bishwajit, G., et al. (2017). "Diabetes mellitus and high blood pressure in relation to BMI among adult non-pregnant women in Bangladesh." Diabetes Metab Syndr 11 Suppl 1: S217-S221.					ID	non_specific	STI	adulthood	reproductive age
84	Bishwajit, G., et al. (2017). "Diabetes mellitus and high blood pressure in relation to BMI among adult non-pregnant women in Bangladesh." Diabetes Metab Syndr 11 Suppl 1: S217-S221.					Symptoms	digestive	diarrhea	childhood	Pre-school child
87	Biswas, R. K., et al. (2019). "Double burden of underweight and overweight among women of reproductive age in Bangladesh." Public health nutrition 22(17): 3163-3174.					Symptoms	multiple/non-specific	fever	childhood	Pre-school child
88	Biswas, T., et al. (2016). "Socio-Economic Inequality of Chronic Non-Communicable Diseases in Bangladesh." PLoS One 11(11): e0167140.									
89	Biswas, T., et al. (2017). "The prevalence of underweight, overweight and obesity in Bangladeshi adults: Data from a national survey." PLoS One 12(5): e0177395.									
90	Biswas, T., et al. (2019). "Association between socioeconomic status and prevalence of non-communicable diseases risk factors and comorbidities in Bangladesh: findings from a nationwide cross-sectional survey." BMJ Open 9(3): e025538.									
119	Chowdhury and M. M. H. Khan (2016). "Risk Factors for Child Malnutrition in Bangladesh: A Multilevel Analysis of a Nationwide Population-Based Survey (vol 172, pg 194, 2016)." Journal of Pediatrics 172: 232-232.									
121	Chowdhury, M. A. B., et al. (2021). "Changes in prevalence and risk factors of hypertension among adults in Bangladesh: An analysis of two waves of nationally representative surveys." PLoS One 16(12): e0259507.									
122	Chowdhury, M. A., et al. "Type 2 diabetes and its correlates among adults in Bangladesh: a population based study." BMC Public Health 15: 1070.									
123	Chowdhury, M. A., et al. (2016). "Hypertension among adults in Bangladesh: evidence from a national cross-sectional survey." BMC Cardiovasc Disord 16: 22.									
124	Chowdhury, M. R., et al. (2016). "Risk Factors for Child Malnutrition in Bangladesh: A Multilevel Analysis of a Nationwide Population-Based Survey." J Pediatr 172: 194-201 e191.									
175	Harshfield, E., et al. (2015). "Association of hypertension and hyperglycaemia with socioeconomic contexts in resource-poor settings: the Bangladesh Demographic and Health Survey." International Journal of Epidemiology 44(5): 1625-1636.									
176	Hasan, M. M., et al. (2019). "Socioeconomic Inequalities of Undiagnosed Diabetes in a Resource-Poor Setting: Insights from the Cross-Sectional Bangladesh Demographic and Health Survey 2011." International journal of environmental research and public health 16(1).									
187	Hoque, A., et al. (2019). "Health inequality in Bangladeshi children of age 6-59 months using hemoglobin data: a nonparametric analysis." Journal of Developing Areas 53(3): 0041.									
189	Hoque, M. E., et al. (2017). "Double burden of underweight and overweight among Bangladeshi adults differs between men and women: evidence from a nationally representative survey." Public health nutrition 20(12): 2183-2191.									

212	Islam, G. M. R. (2017). "The risk of developing cardiovascular disease in Bangladesh: does diabetes mellitus matter? Which socioeconomic status does it impact? A cross sectional study." Journal of the American Society of Hypertension : JASH 11(1): 45-53.e41.									
218	Islam, M. T., et al. (2020). "Influence of height on blood pressure and hypertension among Bangladeshi adults." International Journal of Cardiology. Hypertension 5: 100028.									
229	Jesmin, S., et al. (2015). "A nationwide assessment of hypertension among the adult population in Bangladesh." Journal of Hypertension 33: e17.									
230	Jesmin, S., et al. (2015). "Nationwide prevalence, awareness, treatment and control of hypertension among the adult population in Bangladesh." European Heart Journal 36: 849-1187.									
242	Kamruzzaman, M., et al. (2015). "Differentials in the prevalence of anemia among non-pregnant, ever-married women in Bangladesh: multilevel logistic regression analysis of data from the 2011 Bangladesh Demographic and Health Survey." BMC women's health 15: 54.									
254	Khan, J. R., et al. (2021). "A negative association between prevalence of diabetes and urban residential area greenness detected in nationwide assessment of urban Bangladesh." Sci Rep 11(1): 19513.									
257	Khan, M. M. H., et al. (2014). "The geography of diabetes among the general adults aged 35 years and older in Bangladesh: recent evidence from a cross-sectional survey." PLoS One 9(10): e110756.									
269	Kibria, G. M. A., et al. (2018). "Determinants of hypertension among adults in Bangladesh as per the Joint National Committee 7 and 2017 American College of Cardiology/American Hypertension Association hypertension guidelines." JOURNAL OF THE AMERICAN SOCIETY OF HYPERTENSION 12(11): e45-e55.									
270	Kibria, G. M. A., et al. (2019). "Differences in prevalence and determinants of hypertension according to rural-urban place of residence among adults in Bangladesh." J Biosoc Sci 51(4): 578-590.									
277	Kothari, M., et al. (2013). "Emerging disease burden transition in developing countries: The case of bangladesh." Annals of Nutrition and Metabolism 63(SUPPL. 1): 360.									
338	National Institute of Population Research and Training (NIPORT), Mitra and Associates, and ICF<br><br>International. 2013. Bangladesh Demographic and Health Survey 2011. Dhaka, Bangladesh and Calverton, Maryland, USA: NIPORT, Mitra and Associates, and ICF International									
362	Phuong Hong, N., et al. (2021). "Adolescent birth and child undernutrition: an analysis of demographic and health surveys in Bangladesh, 1996-2017." Annals reports. 1500: 69-81.									
390	Rahman, M. S., et al. (2015). "Awareness, treatment, and control of diabetes in Bangladesh: a nationwide population-based study." PLoS One 10(2): e0118365.									

392	Rahman, M., et al. (2015). "Socioeconomic differences in the prevalence, awareness, and control of diabetes in Bangladesh." J Diabetes Complications 29(6): 788-793.									
393	Rahman, M., et al. (2017). "Gender differences in hypertension awareness, antihypertensive use and blood pressure control in Bangladeshi adults: findings from a national cross-sectional survey." Journal of health, population, and nutrition 36(1): 23.									
399	Raman, M., et al. (2015). "Prevalence and control of hypertension in Bangladesh: a multilevel analysis of a nationwide population-based survey"									
417	Roy, P. K., et al. (2019). "Exploring socio-demographic-and geographical-variations in prevalence of diabetes and hypertension in Bangladesh: Bayesian spatial analysis of national health survey data." Spatial and spatio-temporal epidemiology 29: 71-83.									
425	Saha, U. R., et al. (2019). "Trends, prevalence and determinants of childhood chronic undernutrition in regional divisions of Bangladesh: Evidence from demographic health surveys, 2011 and 2014." PLoS One 14(8): e0220062.									
425	Saha, U. R., et al. (2019). "Trends, prevalence and determinants of childhood chronic undernutrition in regional divisions of Bangladesh: Evidence from demographic health surveys, 2011 and 2014." PLoS One 14(8): e0220062.									
426	Saha, U. R., et al. (2020). Risk factors and spatial variation of stunting among under-fives in Bangladesh: Challenges to reach the sustainable development goal: 323-345.									
438	Shamima, A., et al. (2014). "Nationwide survey of prevalence and risk factors for diabetes and prediabetes in Bangladeshi adults." Diabetes Care 37(1): e9-e10.									
477	Swasey, K. K., et al. (2020). "Determinants of diabetes in Bangladesh using two approaches: an analysis of the Demographic and Health Survey 2011." Journal of biosocial science 52(4): 585-595.									
481	Talukder, A. and M. Z. Hossain (2020). "Prevalence of Diabetes Mellitus and Its Associated Factors in Bangladesh: Application of Two-level Logistic Regression Model." Sci Rep 10(1): 10237.									
485	Tareque, M. I., et al. (2015). "Are the rates of hypertension and diabetes higher in people from lower socioeconomic status in Bangladesh? Results from a nationally representative survey." PLoS One 10(5): e0127954.									
1	(2016). "Bangladesh Demographic and Health Survey 2014." Bangladesh Demographic and Health Survey 2014: xxiv-pp.	244	bdhs201	Bangladesh	Natioanl representative	NCD	genitourinary	Menopause	adulthood	reproductive age
5	Abdur, R. and S. Nusrat Jahan (2021). "Sociodemographic risk factors of being underweight among ever-married Bangladeshi women of reproductive age: a multilevel analysis." Asia-Pacific journal of public health 33(2/3): 220-226.					NCD	nutritional status	overweight/obese	adulthood	reproductive age
25	Akram, R., et al. (2018). "Prevalence and Determinants of Stunting Among Preschool Children and Its Urban-Rural Disparities in Bangladesh." Food and Nutrition Bulletin 39(4): 521-535.					NCD	nutritional status	undernutrition	childhood	Child
49	Anik, A. I., et al. (2019). "Double burden of malnutrition at household level: A comparative study among Bangladesh, Nepal, Pakistan, and Myanmar." PLoS One 14(8): e0221274.					NCD	nutritional status	undernutrition	adulthood	reproductive age

81	Bishwajit, G. (2017). "Household wealth status and overweight and obesity among adult women in Bangladesh and Nepal." Obesity science & practice 3(2): 185-192.					ID	non_specific	ARI	childhood	Pre-school child
87	Biswas, R. K., et al. (2019). "Double burden of underweight and overweight among women of reproductive age in Bangladesh." Public health nutrition 22(17): 3163-3174.					ID	non_specific	STI	adulthood	reproductive age
141	Das, S. and J. Gulshan (2017). "Different forms of malnutrition among under five children in Bangladesh: a cross sectional study on prevalence and determinants." BMC nutrition 3(1): 1-12.					Symptoms	digestive	diarrhea	childhood	Pre-school child
162	Ghose, B. (2017). "Frequency of TV viewing and prevalence of overweight and obesity among adult women in Bangladesh: a cross-sectional study." BMJ Open 7(1): e014399.					Symptoms	multiple/non-specific	fever	childhood	Pre-school child
180	Hashan, M. R., et al. (2020). "Differences in prevalence and associated factors of underweight and overweight/obesity according to rural-urban residence strata among women of reproductive age in Bangladesh: evidence from a cross-sectional national survey." BMJ Open 10(2): e034321.									
191	Hossain, F. B., et al. (2020). "Double burden of malnutrition in children aged 24 to 59 months by socioeconomic status in five South Asian countries: evidence from demographic and health surveys." BMJ Open 10(3): e032866.									
310	Mia, M. N., et al. (2018). "Sociodemographic and geographical inequalities in under- and overnutrition among children and mothers in Bangladesh: a spatial modelling approach to a nationally representative survey." Public Health Nutr 21(13): 2471-2481.									
319	Mohsena, M., et al. (2010). "Association between socio-economic status and childhood undernutrition in Bangladesh; a comparison of possession score and poverty index." Public Health Nutr 13(10): 1498-1504.									
362	Phuong Hong, N., et al. (2021). "Adolescent birth and child undernutrition: an analysis of demographic and health surveys in Bangladesh, 1996-2017." Annals reports. 1500: 69-81.									
382	Rahman, M. A., et al. (2019). "The double burden of under- and overnutrition among Bangladeshi women: Socioeconomic and community-level inequalities." PLoS One 14(7): e0219968.									
383	Rahman, M. A., et al. (2020). "Risk factors of chronic childhood malnutrition: an analysis of the Bangladesh demographic and health survey 2014 data." Journal of Public Health 30(2): 309-321.									
391	Rahman, M. S., et al. (2020). "Prevalence of undernutrition in Bangladeshi children." Journal of biosocial science 52(4): 596-609.									
425	Saha, U. R., et al. (2019). "Trends, prevalence and determinants of childhood chronic undernutrition in regional divisions of Bangladesh: Evidence from demographic health surveys, 2011 and 2014." PLoS One 14(8): e0220062.									
425	Saha, U. R., et al. (2019). "Trends, prevalence and determinants of childhood chronic undernutrition in regional divisions of Bangladesh: Evidence from demographic health surveys, 2011 and 2014." PLoS One 14(8): e0220062.									
430	Sarker, A. R., et al. (2016). "Prevalence and Health Care-Seeking Behavior for Childhood Diarrheal Disease in Bangladesh." Glob Pediatr Health 3: 2333794X16680901.									

469	Sultana, M., et al. (2019). "Prevalence, determinants and health care-seeking behavior of childhood acute respiratory tract infections in Bangladesh." PLoS One 14(1): e0210433.									
509	Wali, N., et al. (2021). "Wasting and associated factors among children under 5 years in five south Asian countries (2014-2018): analysis of demographic health surveys." International journal of environmental research and public health 18(9).									
17	Afrin, I., et al. (2021). "Demographic, socioeconomic, and biological correlates of hypertension in an adult population: evidence from the Bangladesh demographic and health survey 2017-18." BMC Public Health 21(1229).	245	bdhs201	Bangladesh	Natioanl representative	NCD	cvd/circulatory	elevated blood pressure/ hypertension	adulthood	adulthood
32	Al Kibria, G. M., et al. (2021). "Prevalence, awareness, and control of hypertension among Bangladeshi adults: an analysis of demographic and health survey 2017-18." Clin Hypertens 27(1): 17.					NCD	endocrine and metabolic	elevated glucose/ prediabetes/ diabetes	adulthood	adulthood
33	Al-Zubayer, M. A., et al. "Double and triple burden of non-communicable diseases and its determinants among adults in Bangladesh: Evidence from a recent demographic and health survey." International journal of clinical practice.					NCD	genitourinary	Menopause	adulthood	reproductive age
50	Anik, A. I., et al. (2021). "Urban-rural differences in the associated factors of severe under-5 child undernutrition based on the composite index of severe anthropometric failure (CISAF) in Bangladesh." BMC Public Health 21(1): 2147.					NCD	nutritional status	overweight/obese	adulthood	adulthood
68	Bangladesh demographic and health survey 2018					NCD	nutritional status	undernutrition	childhood	Pre-school child
118	Chow, C. K., et al. (2013). "Prevalence, awareness, treatment, and control of hypertension in rural and urban communities in high-, middle-, and low-income countries." JAMA 310(9): 959-968.					NCD	nutritional status	undernutrition	adulthood	adulthood
121	Chowdhury, M. A. B., et al. (2021). "Changes in prevalence and risk factors of hypertension among adults in Bangladesh: An analysis of two waves of nationally representative surveys." PLoS One 16(12): e0259507.					NCD	pregnancy, childbirth and the puerperium	childbirth	Missing	Missing
164	Gupta, R. D., et al. (2021). "Association of household wealth and education level with hypertension and diabetes among adults in Bangladesh: a propensity score-based analysis." Tropical medicine & international health : TM & IH.					NCD	pregnancy, childbirth and the puerperium	perinatal mortality	adulthood	reproductive age
192	Hossain, M. B., et al. (2021). "Role of hypertension in the association of overweight and obesity with diabetes among adults in Bangladesh: a population-based, cross-sectional nationally representative survey." BMJ Open 11(7): e050493.					ID	non_specific	ARI	childhood	Pre-school child
252	Khan, J. R. and J. Gulshan (2021). "Assessing the double burden of malnutrition among Bangladeshi reproductive-aged women: A comparison between unconditional and conditional quantile regression." Health Science Reports 4(4): e391.					Symptoms	digestive	diarrhea	childhood	Pre-school child
255	Khan, J. R., et al. (2021). "Household cooking fuels associated with elevated blood pressure among adult women: a national-wide assessment in Bangladesh." Environmental science and pollution research international.					Symptoms	multiple/non-specific	fever	childhood	Pre-school child
256	Khan, J. R., et al. (2021). "Relationship between blood pressure and body mass index in young adult population: A national-level assessment in Bangladesh." British Journal of Nutrition.									

264	Khanam, M., et al. (2021). "Prevalence and Factors of Hypertension Among Bangladeshi Adults." High Blood Pressure and Cardiovascular Prevention 28(4): 393-403.									
305	Mansura, K., et al. (2021). "Underweight, overweight and obesity among reproductive Bangladeshi women: a nationwide survey." Nutrients 13(12).									
362	Phuong Hong, N., et al. (2021). "Adolescent birth and child undernutrition: an analysis of demographic and health surveys in Bangladesh, 1996-2017." Annals reports. 1500: 69-81.									
384	Rahman, M. A., et al. (2021). "Prevalence of and factors associated with hypertension according to JNC 7 and ACC/AHA 2017 guidelines in Bangladesh." Scientific reports 11(1): 15420.									
385	Rahman, M. A., et al. (2021). "Prevalence of and factors associated with prehypertension and hypertension among Bangladeshi young adults: an analysis of the Bangladesh demographic and health survey 2017-18." Clinical Epidemiology and Global Health 12.									
429	Sarker, A. R. and M. Khanam "Socio-economic inequalities in diabetes and prediabetes among Bangladeshi adults." Diabetology International.									
76	Bhowmik, B., et al. (2012). "Prevalence of type 2 diabetes and impaired glucose regulation with associated cardiometabolic risk factors and depression in an urbanizing rural community in bangladesh: a population-based cross-sectional study." Diabetes Metab J 36(6): 422-432.	256		Bangladesh	Rural	NCD	cvd/circulatory	elevated blood pressure/ hypertension	adulthood	adulthood
78	Bhowmik, B., et al. (2014). "Anthropometric indices of obesity and type 2 diabetes in Bangladeshi population: Chandra Rural Diabetes Study (CRDS)." Obesity research & clinical practice 8(3): e201-298.					NCD	endocrine and metabolic	elevated cholesterol/ hyperlipidemia	adulthood	adulthood
442	Siddiquee, T., et al. (2015). "Prevalence of obesity in a rural Asian Indian (Bangladeshi) population and its determinants." BMC Public Health 15: 860.					NCD	endocrine and metabolic	elevated glucose/ prediabetes/ diabetes	adulthood	adulthood
						NCD	nutritional status	overweight/obese	adulthood	adulthood
						NCD	nutritional status	undernutrition	adulthood	adulthood
						NCD	psycological/ mental	depression	adulthood	adulthood
						SPH	qol	qol	adulthood	adulthood
63	Bangladesh Multiple Indicator Cluster Survey 2012-2013, ProgotirPathey: Final Report. Bangladesh Bureau of Statistics (BBS) and UNICEF Bangladesh, 2014, Dhaka, Bangladesh	246	bmics20	Bangladesh	Natioanl representative	NCD	child development	low birth weight	childhood	Pre-school child
125	Chowdhury, T. R., et al. (2018). "Socio-economic risk factors for early childhood underweight in Bangladesh." Globalization and health 14(1): 54.					NCD	nutritional status	overweight/obese	childhood	Pre-school child
126	Chowdhury, T. R., et al. (2020). "Factors associated with stunting and wasting in children under 2 years in Bangladesh." Heliyon 6(9): e04849.					NCD	nutritional status	undernutrition	childhood	Pre-school child
216	Islam, M. M., et al. (2019). "Does parental migration have any impact on nutritional disorders among left-behind children in Bangladesh?" Public Health Nutr 22(1): 95-103.					NCD	psycological/ mental	aggressive behavior	childhood	Child
253	Khan, J. R., et al. (2018). "Analysis of low birth weight and its co-variants in Bangladesh based on a sub-sample from nationally representative survey." BMC Pediatr 18(1): 100.					ID	non_specific	ARI	childhood	Pre-school child
						Symptoms	digestive	diarrhea	childhood	Pre-school child
64	Bangladesh Bureau of Statistics (BBS) and UNICEF Bangladesh. 2019. Progotir Pathey, Bangladesh Multiple Indicator Cluster Survey 2019, Survey Findings Report. Dhaka, Bangladesh: Bangladesh Bureau of Statistics (BBS).	247	bmics20	Bangladesh	Natioanl representative	NCD	nutritional status	overweight/obese	childhood	Pre-school child

127	Chowdhury, T. R., et al. (2021). "Effects of Parental Education and Wealth on Early Childhood Stunting in Bangladesh." Archives of Public Health.					NCD	nutritional status	undernutrition	childhood	Pre-school child
						NCD	pregnancy, childbirth and the puerperium	antepartum	adulthood	reproductive age
						NCD	pregnancy, childbirth and the puerperium	postpartum	adulthood	reproductive age
						NCD	psycological/ mental	aggressive behavior	childhood	Child
						NCD	psycological/ mental	wellbeing	adulthood	reproductive age
						ID	non_specific	ARI	childhood	Pre-school child
						ID	non_specific	Uterine infection	adulthood	reproductive age
						Symptoms	digestive	diarrhea	childhood	Pre-school child
						Symptoms	multiple/non-specific	fever	childhood	Pre-school child
						Disability/Lim	Function &Activity	Function &Activity	childhood	Pre-school child
144	De, P. K. and N. S. Murshid (2018). "Associations of intimate partner violence with screening for mental health disorders among women in urban Bangladesh." International journal of public health 63(8): 913-921.	248	buhs200	Bangladesh	Urban	NCD	cvd/circulatory	elevated blood pressure/ hypertension	Missing	Missing
482	Talukder, S., et al. (2010). "Hypertension disparities among place of residence: Findings from Bangladesh Urban Health Survey 2006." Circulation 122(2): e79.					NCD	psycological/ mental	suicidality	adulthood	reproductive age and older adulthood
						Symptoms	neurology	headache	adulthood	reproductive age and older adulthood
21	Ahsan, K. Z., et al. (2017). "Effects of individual, household and community characteristics on child nutritional status in the slums of urban Bangladesh." Archives of public health = Archives belges de sante publique 75: 9.	249	buhs201	Bangladesh	Natioanl representative	NCD	nutritional status	overweight/obese	childhood	Pre-school child
67	Bangladesh Urban Health Survey 2013					NCD	nutritional status	undernutrition	childhood	Pre-school child
177	Hasan, M., et al. (2019). "Association of biomass fuel smoke with respiratory symptoms among children under 5 years of age in urban areas: results from Bangladesh Urban Health Survey, 2013." Environmental health and preventive medicine 24(1): 65.					NCD	pregnancy, childbirth and the puerperium	antepartum	Missing	Missing
219	Islam, M. and N. Sultana (2019). "Risk factors for pregnancy related complications among urban slum and non-slum women in Bangladesh." BMC Pregnancy & Childbirth 19(1): N.PAG-N.PAG.					NCD	pregnancy, childbirth and the puerperium	childbirth	adulthood	reproductive age
						ID	non_specific	ARI	childhood	Pre-school child
						Symptoms	respiratory	cough	childhood	Pre-school child
						Symptoms	respiratory	dyspnea/shortness of breath	childhood	Pre-school child
259	Khan, N., et al. (2019). "Prevalence of multimorbidity among Bangladeshi adult population: a nationwide cross-sectional study." BMJ Open 9(11): e030886.	257		Bangladesh	Natioanl representative	NCD	cvd/circulatory	Stroke	adulthood	adulthood
312	Mistry, S. K., et al. (2019). "Individual-, maternal- and household-level factors associated with stunting among children aged 0-23 months in Bangladesh." Public Health Nutr 22(1): 85-94.					NCD	cvd/circulatory	elevated blood pressure/ hypertension	adulthood	adulthood
315	Mitra, D. K., et al. (2018). "Demographic, Socio-economic and Lifestyle Determinants of Under- and Over-nutrition among Bangladeshi Adult Population: Results from a Large Cross-Sectional Study." J Epidemiol Glob Health 8(3-4): 134-142.					NCD	cvd/circulatory	heart disease	adulthood	adulthood

531	Yunus, F. M., et al. (2018). "Relationship of sleep pattern and snoring with chronic disease: findings from a nationwide population-based survey." Sleep Health 4(1): 40-48.					NCD	endocrine and metabolic	elevated glucose/ prediabetes/ diabetes	adulthood	adulthood
						NCD	neoplasma	cancer	adulthood	adulthood
						NCD	nutritional status	overweight/obese	adulthood	adulthood
						NCD	nutritional status	undernutrition	childhood	Pre-school child
						NCD	nutritional status	undernutrition	adulthood	adulthood
						NCD	respiratory	COPD	adulthood	adulthood
						General health			adulthood	adulthood
82	Bishwajit, G., et al. (2017). "Association between depression and fruit and vegetable consumption among adults in South Asia." BMC psychiatry 17(1): 15.	250	bwhs	Bangladesh	Natioanl representative	NCD	Oral health	teeth	adulthood	adulthood
83	Bishwajit, G., et al. (2017). "Burden of asthma, dyspnea, and chronic cough in South Asia." International Journal of COPD 12: 1093-1099.					NCD	musculoskeleton	Arthritis	adulthood	adulthood
85	Bishwajit, G., et al. (2017). "Participation in physical activity and back pain among an elderly population in South Asia." Journal of pain research 10: 905-913.					NCD	musculoskeleton	Soreness/Pain	adulthood	older adulthood and retirement age
86	Bishwajit, G., et al. (2017). "Physical inactivity and self-reported depression among middle- and older-aged population in South Asia: World health survey." BMC geriatrics 17(1): 100.					NCD	psycological/ mental	Psychosis	adulthood	adulthood
131	Cifuentes, M., et al. (2010). "National income, self-reported wheezing and asthma diagnosis from the World Health Survey." European Respiratory Journal 35(2): 279-286.					NCD	psycological/ mental	depression	adulthood	adulthood
137	Cristobal-Narvaez, P., et al. (2020). "Perceived stress and depression in 45 low- and middle-income countries." Journal of Affective Disorders 274: 799-805.					NCD	respiratory	asthma	adulthood	adulthood
348	Nuevo, R., et al. (2012). "The continuum of psychotic symptoms in the general population: a cross-national study." Schizophr Bull 38(3): 475-485.					Symptoms	respiratory	cough	adulthood	adulthood
462	Stubbs, B., et al. (2016). "Physical activity and depression: a large cross-sectional, population-based study across 36 low- and middle-income countries." Acta Psychiatrica Scandinavica 134(6): 546-556.					Symptoms	respiratory	dyspnea/shortness of breath	adulthood	adulthood
463	Stubbs, B., et al. (2017). "Lifetime self-reported arthritis is associated with elevated levels of mental health burden: A multi-national cross sectional study across 46 low- and middle-income countries." Sci Rep 7(1): 7138.					General health			adulthood	adulthood
499	To, T., et al. (2012). "Global asthma prevalence in adults: findings from the cross-sectional world health survey." BMC Public Health 12: 204.					Disability/Lim	Overall	Overall	adulthood	adulthood
501	Tyrovolas, S., et al. (2016). "Population prevalence of edentulism and its association with depression and self-rated health." Scientific reports 6: 37083.					SPH	qol	qol	adulthood	adulthood
						SPH	srh	srh	adulthood	adulthood
45	An, Y., et al. (2013). "Appropriate Body Mass Index and Waist Circumference Cutoff for Overweight and Central Obesity among Adults in Cambodia." PLoS One 8(10): e77897.	258	c-steps2	Cambodia	Natioanl representative	NCD	cvd/circulatory	elevated blood pressure/ hypertension	adulthood	reproductive age and older adulthood
99	Cambodia STEPS 2010					NCD	endocrine and metabolic	elevated cholesterol/ hyperlipidemia	adulthood	reproductive age and older adulthood
165	Gupta, V., et al. (2013). "The prevalence and associated factors for prehypertension and hypertension in Cambodia." Heart Asia 5(1): 253-258.					NCD	endocrine and metabolic	elevated glucose/ prediabetes/ diabetes	adulthood	reproductive age and older adulthood

						NCD	nutritional status	overweight/obese	adulthood	reproductive age and older adulthood
						NCD	nutritional status	undernutrition	adulthood	reproductive age and older adulthood
306	Mao, T. E., et al. (2014). "Cross-sectional studies of tuberculosis prevalence in Cambodia between 2002 and 2011." Bulletin of the World Health Organization 92(8): 573-581.	259	c-tb201(	Cambodia	Natioanl representative	ID	bacteria	TB	adulthood	adulthood
436	National Tuberculosis Control Program (2012), Report: Second National Tuberculosis Prevalence Survey Cambodia, 2011, Phnom Penh, Cambodia									
100	Royal University of Phnom Penh Department of Psychology, (2012), Cambodian Mental health survey 2012	263	cambod	Cambodia	Natioanl representative	NCD	ear	tinnitus	adulthood	adulthood
182	Hinton, D. E., et al. (2019). "Culturally sensitive assessment of anxious-depressive distress in the Cambodian population: Avoiding category truncation." Transcultural Psychiatry 56(4): 643-666.					NCD	musculoskeleton	Soreness/Pain	adulthood	adulthood
						NCD	psycological/ mental	aggressive behavior	adulthood	adulthood
						NCD	psycological/ mental	anxiety	adulthood	adulthood
						NCD	psycological/ mental	depression	adulthood	adulthood
						NCD	psycological/ mental	ptsd	adulthood	adulthood
						NCD	psycological/ mental	suicidality	adulthood	adulthood
						Symptoms	multiple/non-specific	dizziness	adulthood	adulthood
						Symptoms	multiple/non-specific	weakness	adulthood	adulthood
						Disability/Lim	Function	Function Vision	adulthood	adulthood
101	Cantor-Graae, E., et al. (2014). "Long-term psychiatric consequences of exposure to trauma in Cambodia: a regional household survey." Social science & medicine (1982) 123: 133-140.	264		Cambodia	Rural	NCD	psycological/ mental	anxiety	adulthood	reproductive age and older adulthood
227	Jarl, J., et al. (2015). "Trauma and Poor Mental Health in Relation to Economic Status: The Case of Cambodia 35 Years Later." PLoS One 10(8): e0136410.					NCD	psycological/ mental	depression	adulthood	reproductive age and older adulthood
						NCD	psycological/ mental	ptsd	adulthood	reproductive age and older adulthood
						Disability/Lim	Activity ADL	Activity ADL	adulthood	reproductive age and older adulthood
						SPH	qol	qol	adulthood	reproductive age and older adulthood
138	Darapheak, C., et al. (2013). "Consumption of animal source foods and dietary diversity reduce stunting in children in Cambodia." Int Arch Med 6(1): 29.	260	cdhs200	Cambodia	Natioanl representative	NCD	nutritional status	undernutrition	childhood	Pre-school child
						Symptoms	digestive	diarrhea	childhood	Pre-school child
340	National Institute of Statistics, Directorate General for Health, and ICF Macro, 2011. Cambodia Demographic<br><br>and Health Survey 2010. Phnom Penh, Cambodia and Calverton, Maryland, USA: National Institute of<br><br>Statistics, Directorate General for Health, and ICF Macro.	261	cdhs201	Cambodia	Natioanl representative	NCD	Injury	Non-specific	lifespan	lifespan

						NCD	blood and blood forming organ	Anemia	adulthood	reproductive age
						NCD	nutritional status	overweight/obese	adulthood	reproductive age
						NCD	nutritional status	undernutrition	childhood	Pre-school child
						ID	non_specific	ARI	childhood	Pre-school child
						Symptoms	digestive	diarrhea	childhood	Pre-school child
						Symptoms	multiple/non-specific	fever	childhood	Pre-school child
						General health			lifespan	lifespan
						Disability/Lim	Body structure	Body structure	lifespan	lifespan
115	Chhea, C., et al. (2018). "Low birth weight of institutional births in Cambodia: Analysis of the Demographic and Health Surveys 2010-2014." PLoS One 13(11): 16.	262	cdhs201	Cambodia	Natioanl representative	NCD	Injury	Non-specific	lifespan	lifespan
339	National Institute of Statistics, Directorate General for Health, and ICF International, 2015. Cambodia<br><br>Demographic and Health Survey 2014. Phnom Penh, Cambodia, and Rockville, Maryland, USA: National<br><br>Institute of Statistics, Directorate General for Health, and ICF International.					NCD	blood and blood forming organ	Anemia	adulthood	reproductive age
364	Pisey, V., et al. (2021). "The association of socio-demographic and environmental factors on childhood diarrhea in Cambodia." F1000Research 9: 303.					NCD	blood and blood forming organ	Hemoglobinpathy	childhood	Pre-school child
450	Smith, G., et al. (2016). "High Prevalence of Vitamin D Deficiency in Cambodian Women: A Common Deficiency in a Sunny Country." Nutrients 8(5).					NCD	child development	low birth weight	adulthood	reproductive age
516	Whitfield, K. C., et al. (2017). "High prevalence of thiamine (vitamin B1) deficiency in early childhood among a nationally representative sample of Cambodian women of childbearing age and their children." PLoS neglected tropical diseases 11(9): e0005814.					NCD	nutritional status	micronutrition deficiency	adulthood	reproductive age
522	Wieringa, F. T., et al. (2016). "The High Prevalence of Anemia in Cambodian Children and Women Cannot Be Satisfactorily Explained by Nutritional Deficiencies or Hemoglobin Disorders." Nutrients 8(6).					NCD	nutritional status	overweight/obese	adulthood	reproductive age
						NCD	nutritional status	undernutrition	childhood	Pre-school child
						ID	non_specific	ARI	childhood	Pre-school child
						ID	parasite	intestine parasite	childhood	Pre-school child
						Symptoms	digestive	diarrhea	childhood	Pre-school child
						Symptoms	multiple/non-specific	fever	childhood	Pre-school child
						General health			lifespan	lifespan
						Disability/Lim	Function &Activity	Function &Activity	lifespan	lifespan
154	Esie, P., et al. (2021). "Social norms and the association between intimate partner violence and depression in rural Bangladesh-a multilevel analysis." Soc Psychiatry Psychiatr Epidemiol 56(12): 2217-2226.	265		Bangladesh	Rural	NCD	psycological/ mental	depression	Missing	Missing
75	Bhan, N., et al. (2017). "Socioeconomic patterning of chronic conditions and behavioral risk factors in rural South Asia: a multi-site cross-sectional study." Int J Public Health 62(9): 1019-1028.	266		Bangladesh	Rural	NCD	cvd/circulatory	elevated blood pressure/ hypertension	adulthood	adulthood
						NCD	endocrine and metabolic	elevated glucose/ prediabetes/ diabetes	adulthood	adulthood

						NCD	nutritional status	overweight/obese	adulthood	adulthood
						NCD	psycological/ mental	depression	adulthood	adulthood
						Symptoms	respiratory	impaired lung function	adulthood	adulthood
						Disability/Lim	Function Vision	Function Vision	adulthood	adulthood
						Disability/Lim	Overall	Overall	adulthood	adulthood
128	Chu, C. H., et al. (2012). "Oral health status and behaviours of children in Myanmar - a pilot study in four villages in rural areas." Oral Health Prev Dent 10(4): 365-371.	217		Myanmar	Rural	NCD	Oral health	teeth	childhood	School-age child
						SPH	qol	qol	childhood	School-age child
132	Conlan, J. V., et al. (2012). "A cross-sectional study of Taenia solium in a multiple taeniid-endemic region reveals competition may be protective." Am J Trop Med Hyg 87(2): 281-291.	267		Laos	Rural	ID	parasite	IP-STH	lifespan	lifespan
133	Conlan, J. V., et al. (2012). "Soil-transmitted helminthiasis in Laos: a community-wide cross-sectional study of humans and dogs in a mass drug administration environment." Am J Trop Med Hyg 86(4): 624-634.					ID	parasite	IP-nematodes (trichinella)	lifespan	lifespan
134	Conlan, J. V., et al. (2014). "Patterns and risks of trichinella infection in humans and pigs in northern Laos." PLoS Negl Trop Dis 8(7): e3034.					ID	parasite	tapeworm (Taeniasis, cysticercosis)	lifespan	lifespan
135	Conlan, J. V., et al. (2015). "Patterns of Flavivirus Seroprevalence in the Human Population of Northern Laos." Am J Trop Med Hyg 93(5): 1010-1013.					ID	virus	dengue	lifespan	lifespan
						ID	virus	je	lifespan	lifespan
378	Psaki, S., et al. (2012). "Household food access and child malnutrition: results from the eight-country MAL-ED study." Popul Health Metr 10(1): 24.	268		Bangladesh	Urban	NCD	nutritional status	undernutrition	childhood	Pre-school child
147	Dickson, B. F. R., et al. (2018). "The prevalence of lymphatic filariasis infection and disease following six rounds of mass drug administration in Mandalay Region, Myanmar." PLoS neglected tropical diseases 12(11): e0006944.	269		Myanmar	Rural	NCD	genitourinary	hydrocoele	adulthood	adulthood
148	Dickson, B. F. R., et al. (2021). "Risk factors for lymphatic filariasis and mass drug administration non-participation in Mandalay Region, Myanmar." Parasit Vectors 14(1): 72.					ID	parasite	intestine parasite	lifespan	lifespan
						ID	parasite	lymphatic filariasis	lifespan	lifespan
184	Ho, F. K., et al. (2021). "Association of Early Nutritional Status With Child Development in the Asia Pacific Region." JAMA Network Open 4(12): e2139543.	270		Cambodia	Natioanl representative	NCD	nutritional status	undernutrition	childhood	Pre-school child
400	Rao, N., et al. (2020). "Associations Among Early Stimulation, Stunting, and Child Development in Four Countries in the East Asia–Pacific." International Journal of Early Childhood 52(2): 175-193.									
226	Janssen, M. F., et al. (2019). "Population norms for the EQ-5D-3L: a cross-country analysis of population surveys for 20 countries." European Journal of Health Economics 20(2): 205-216.	271		Thailand	Not specified	SPH	qol	qol	adulthood	adulthood
29	Akuze, J., et al. (2020). "Randomised comparison of two household survey modules for measuring stillbirths and neonatal deaths in five countries: the Every Newborn-INDEPTH study." The Lancet. Global health 8(4): e555-e566.	272	every ne	Bangladesh	Urban	NCD	pregnancy, childbirth and the puerperium	childbirth	adulthood	reproductive age
117	Choudhury, N., et al. (2017). "Determinants of age-specific undernutrition in children aged less than 2 years-the Bangladesh context." Matern Child Nutr 13(3).	273		Bangladesh	Natioanl representative	NCD	nutritional status	overweight/obese	childhood	Pre-school child
						NCD	nutritional status	undernutrition	childhood	Pre-school child

157	Forrer, A., et al. (2012). "Spatial distribution of, and risk factors for, Opisthorchis viverrini infection in southern Lao PDR." PLoS Negl Trop Dis 6(2): e1481.	274		Laos	Rural	NCD	blood and blood forming organ	Anemia	childhood	Child
158	Forrer, A., et al. (2015). "Risk profiling of hookworm infection and intensity in southern Lao People's Democratic Republic using Bayesian models." PLoS Negl Trop Dis 9(3): e0003486.					NCD	nutritional status	undernutrition	childhood	Child
434	Sayasone, S., et al. (2015). "Multiparasitism and intensity of helminth infections in relation to symptoms and nutritional status among children: a cross-sectional study in southern Lao People's Democratic Republic." Acta Trop 141(Pt B): 322-331.					ID	parasite	intestine parasite	childhood	Child
						Symptoms	digestive	multiple symptoms	childhood	Child
464	S. Iruday Rajan Sreerupa (2017), Study on accessing health care by older population in Myanmar	332	health a	Myanmar	Natioanl representative	General health			adulthood	older adulthood and retirement age
526	Yamada, H., et al. (2020). "Geriatric Depressive Symptoms in Myanmar: Incidence and Associated Factors." J Appl Gerontol 39(11): 1230-1239.					Disability/Lim	Activity ADL	Activity ADL	adulthood	older adulthood and retirement age
						Disability/Lim	Function &Activity	Function &Activity	adulthood	older adulthood and retirement age
						SPH	srh	srh	adulthood	older adulthood and retirement age
34	Alam, M. Z. "Women outweighed men at life expectancy in Bangladesh: does it mean a better quality of life?" Heliyon 7(7): e07618.	333		Bangladesh	Not specified	NCD	psycological/ mental	depression	adulthood	older adulthood and retirement age
						SPH	qol	qol	adulthood	older adulthood and retirement age
34	Alam, M. Z. "Women outweighed men at life expectancy in Bangladesh: does it mean a better quality of life?" Heliyon 7(7): e07618.	275	househo	Bangladesh	Natioanl representative	General health			adulthood	older adulthood and retirement age
142	Datta, B. K., et al. (2018). "Noncommunicable disease-attributable medical expenditures, household financial stress and impoverishment in Bangladesh." SSM - Population Health 6: 252-258.					Disability/Lim	Function &Activity	Function &Activity	adulthood	older adulthood and retirement age
217	Islam, M. S., et al. (2017). "Urban-rural differences in disability-free life expectancy in Bangladesh using the 2010 HIES data." PLoS One 12(7): e0179987.					Disability/Lim	Overall	Overall	adulthood	older adulthood and retirement age
407	Report of the household income & expenditure survey 2010									
484	Tareque, M. I., et al. (2013). "Gender Differences in Disability-Free Life Expectancy at Old Ages in Bangladesh." Journal of Aging & Health 25(8): 1299-1312.									
488	Tareque, M. I., et al. (2017). "Gender differences in functional disability and self-care among seniors in Bangladesh." BMC Geriatr 17(1): 177.									
372	Pothisiri, W. and P. M. M. Vicerra (2021). "Psychological distress during COVID-19 pandemic in low-income and middle-income countries: A cross-sectional study of older persons in Thailand." BMJ Open 11(4): e047650.	276		Thailand	Rural and urban	NCD	psycological/ mental	psychological distress	adulthood	older adulthood and retirement age
						Disability/Lim	Function &Activity	Function &Activity	adulthood	older adulthood and retirement age
						SPH	srh	srh	adulthood	older adulthood and retirement age

10	Aekplakorn, W., et al. (2011). "Prevalence of metabolic syndrome defined by the International Diabetes Federation and National Cholesterol Education Program criteria among Thai adults." Asia Pac J Public Health 23(5): 792-800.	277	interasia	Thailand	Rural and urban	NCD	endocrine and metabolic	Metabolic Syndrome	adulthood	adulthood
206	Islam, F. M. A., et al. (2015). "Factors Associated with Awareness, Attitudes and Practices Regarding Common Eye Diseases in the General Population in a Rural District in Bangladesh: The Bangladesh Population-based Diabetes and Eye Study (BPDES)." PLoS One 10(7): e0133043.	278		Bangladesh	Rural	NCD	cvd/circulatory	elevated blood pressure/ hypertension	adulthood	adulthood
207	Islam, F. M. A., et al. (2015). "Rationale and methodology for a population-based study of diabetes and common eye diseases in a rural area in Bangladesh: Bangladesh Population based Diabetes and Eye Study (BPDES)." Bangladesh Journal of Medical Science 14(4): 367-375.					NCD	endocrine and metabolic	elevated glucose/ prediabetes/ diabetes	adulthood	adulthood
						NCD	eye	eye problem	adulthood	adulthood
213	Islam, J. Y., et al. (2018). "Epidemiology of hypertension among Bangladeshi adults using the 2017 ACC/AHA Hypertension Clinical Practice Guidelines and Joint National Committee 7 Guidelines." J Hum Hypertens 32(10): 668-680.	279		Bangladesh	Natioanl representative	NCD	cvd/circulatory	elevated blood pressure/ hypertension	adulthood	adulthood
214	Islam, J. Y., et al. (2019). "Prevalence and determinants of hyperglycaemia among adults in Bangladesh: results from a population-based national survey." BMJ Open 9(7): e029674.					NCD	endocrine and metabolic	elevated glucose/ prediabetes/ diabetes	adulthood	adulthood
						NCD	nutritional status	overweight/obese	adulthood	adulthood
276	Komada, K., et al. (2015). "Seroprevalence of chronic hepatitis B, as determined from dried blood spots, among children and their mothers in central Lao People's Democratic Republic: A multistage, stratified cluster sampling survey." International Journal of Infectious Diseases 36: 21-26.	280		Laos	Rural and urban	ID	virus	hbv	adulthood	Reproductive age
347	Norizuki, M., et al. (2019). "Serologic testing of randomly selected children after hepatitis B vaccination: a cross-sectional population-based study in Lao People's Democratic Republic." BMC Infect Dis 19(1): 507.									
357	Pengpid, S., et al. (2020). "The prevalence of underweight and overweight/obesity and its correlates among adults in Laos: a cross-sectional national population-based survey, 2013." Eat Weight Disord 25(2): 265-273.	281	l-steps2(	Laos	Natioanl representative	NCD	cvd/circulatory	elevated blood pressure/ hypertension	adulthood	reproductive age and older adulthood
505	Vonglokham, M., et al. (2019). "Prevalence and social and health determinants of pre-diabetes and diabetes among adults in Laos: a cross-sectional national population-based survey, 2013." Trop Med Int Health 24(1): 65-72.					NCD	endocrine and metabolic	elevated cholesterol/ hyperlipidemia	adulthood	reproductive age and older adulthood
						NCD	endocrine and metabolic	elevated glucose/ prediabetes/ diabetes	adulthood	reproductive age and older adulthood
						NCD	nutritional status	overweight/obese	adulthood	reproductive age and older adulthood
						NCD	nutritional status	undernutrition	adulthood	reproductive age and older adulthood
47	Andersson, M. and A. Lundin (2015). "Socioeconomic Inequalities in Global and Relative Self-Rated Health in Laos: A Cross-sectional Study of 24 162 Men and Women." Asia-Pacific journal of public health 27(2): NP1060-1070.	285	lao expe	Laos	Natioanl representative	General health			adulthood	adulthood
						SPH	srh	srh	adulthood	adulthood
358	Petersen, A. B., et al. (2019). "Smoked tobacco, air pollution, and tuberculosis in lao PDR: Findings from a national sample." International journal of environmental research and public health 16(17): 3059.	286	lao natic	Laos	Natioanl representative	ID	bacteria	TB	adulthood	adulthood

333	Nanthavong, N., et al. (2015). "Diphtheria in Lao PDR: Insufficient Coverage or Ineffective Vaccine?" PLoS One 10(4): e0121749.	335	large va	Laos	Rural	ID	bacteria	diphtheria	childhood	Pre-school child
334	Nanthavong, N., et al. (2017). "High prevalence of intestinal worms in children up to 5 years of age in Huaphan province, Lao People's Democratic Republic (PDR)." Parasite Epidemiol Control 2(3): 114-117.					ID	bacteria	tetanus	childhood	Pre-school child
						ID	parasite	intestine parasite	childhood	Pre-school child
474	Suwannatrai, A. T., et al. (2021). "Opisthorchis viverrini and Strongyloides stercoralis mono- and co-infections: Bayesian geostatistical analysis in an endemic area, Thailand." Acta Trop 223: 106079.	287		Thailand	Rural	ID	parasite	IP-trematodes (O. viverrini) and nematode (S. stercoalis)	adulthood	reproductive age and older adulthood
273	Knowles, J., et al. (2013). "Impact of inflammation on the biomarkers of iron status in a cross-sectional survey of Lao women and children." Br J Nutr 110(12): 2285-2297.	282	lmics20(	Laos	Natioanl representative	NCD	nutritional status	micronutrition deficiency	childhood	Pre-school child
						Symptoms	multiple/non-specific	presence of inflammation (acute-phase proteins)	adulthood	reproductive age
247	Keokenchanh, S., et al. (2021). "Prevalence of Anemia and Its Associate Factors among Women of Reproductive Age in Lao PDR: Evidence from a Nationally Representative Survey." Anemia 2021: 8823030.	283	lsis 2017	Laos	Natioanl representative	NCD	blood and blood forming organ	Anemia	adulthood	reproductive age
291	Lao Statistics Bureau. 2018. Lao Social Indicator Survey II 2017, Survey Findings Report. Vientiane, Lao PDR: Lao Statistics Bureau and UNICEF.					NCD	nutritional status	overweight/obese	adulthood	reproductive age
322	Morita, A., et al. (2021). "Prevalence of anemia and its associated factors among children aged 6-59 months in the Lao People's Democratic Republic: A multilevel analysis." PLoS One 16(3 March): e0248969.					NCD	nutritional status	undernutrition	childhood	Pre-school child
						NCD	nutritional status	undernutrition	adulthood	reproductive age
						NCD	pregnancy, childbirth and the puerperium	childbirth	adulthood	reproductive age
						ID	non_specific	ARI	childhood	Pre-school child
						Symptoms	digestive	diarrhea	childhood	Pre-school child
						Symptoms	multiple/non-specific	fever	childhood	Pre-school child
						Symptoms	respiratory	cough	childhood	Pre-school child
						Disability/Lim	Function &Activity	Function &Activity	childhood	Pre-school child
131	Cifuentes, M., et al. (2010). "National income, self-reported wheezing and asthma diagnosis from the World Health Survey." European Respiratory Journal 35(2): 279-286.	284	lwhs	Laos	Natioanl representative	NCD	Oral health	teeth	adulthood	adulthood
137	Cristobal-Narvaez, P., et al. (2020). "Perceived stress and depression in 45 low- and middle-income countries." Journal of Affective Disorders 274: 799-805.					NCD	musculoskeleton	Arthritis	adulthood	adulthood
348	Nuevo, R., et al. (2012). "The continuum of psychotic symptoms in the general population: a cross-national study." Schizophr Bull 38(3): 475-485.					NCD	psycological/ mental	Psychosis	adulthood	adulthood
463	Stubbs, B., et al. (2017). "Lifetime self-reported arthritis is associated with elevated levels of mental health burden: A multi-national cross sectional study across 46 low- and middle-income countries." Sci Rep 7(1): 7138.					NCD	psycological/ mental	depression	adulthood	adulthood
499	To, T., et al. (2012). "Global asthma prevalence in adults: findings from the cross-sectional world health survey." BMC Public Health 12: 204.					NCD	respiratory	asthma	adulthood	adulthood

501	Tyrovolas, S., et al. (2016). "Population prevalence of edentulism and its association with depression and self-rated health." Scientific reports 6: 37083.					General health			adulthood	adulthood
						Disability/Lim	Overall	Overall	adulthood	adulthood
						SPH	qol	qol	adulthood	adulthood
						SPH	srh	srh	adulthood	adulthood
59	Aung, W. P., et al. (2019). "Trends in Diabetes Prevalence, Awareness, Treatment and Control in Yangon Region, Myanmar, Between 2004 and 2014, Two Cross-Sectional Studies." Int J Environ Res Public Health 16(18).	288	m-steps	Myanmar	Rural and urban	NCD	cvd/circulatory	elevated blood pressure/ hypertension	adulthood	adulthood
274	Ko Ko, Z., et al. (2011). "Prevalence of hypertension and its associated factors in the adult population in Yangon Division, Myanmar." Asia-Pacific journal of public health 23(4): 496-506.					NCD	endocrine and metabolic	elevated cholesterol/ hyperlipidemia	adulthood	adulthood
						NCD	endocrine and metabolic	elevated glucose/ prediabetes/ diabetes	adulthood	adulthood
						NCD	nutritional status	overweight/obese	adulthood	adulthood
91	Bjertness, M. B., et al. (2016). "Prevalence and determinants of hypertension in Myanmar - a nationwide cross-sectional study." BMC Public Health 16: 590.	289	m-steps	Myanmar	Natioanl representative	NCD	cvd/circulatory	elevated blood pressure/ hypertension	adulthood	reproductive age and older adulthood
507	WHO: Noncommunicable disease risk factor survey Myanmar 2009. In. New Delhi: WHO; 2011					NCD	endocrine and metabolic	elevated glucose/ prediabetes/ diabetes	adulthood	reproductive age and older adulthood
						NCD	nutritional status	overweight/obese	adulthood	reproductive age and older adulthood
						NCD	nutritional status	undernutrition	adulthood	reproductive age and older adulthood
410	Report on National survey of diabetes Mellitus and riska factors for Non-communicable diseases in Myanamr (2014)	290	m-steps	Myanmar	Natioanl representative	NCD	cvd/circulatory	elevated blood pressure/ hypertension	adulthood	reproductive age and older adulthood
493	Thapa, R., et al. (2021). "Urban-rural differences in overweight and obesity among 25-64 years old Myanmar residents: a cross-sectional, nationwide survey." BMJ Open 11(3): e042561.					NCD	endocrine and metabolic	elevated cholesterol/ hyperlipidemia	adulthood	reproductive age and older adulthood
						NCD	endocrine and metabolic	elevated glucose/ prediabetes/ diabetes	adulthood	adulthood
						NCD	nutritional status	overweight/obese	adulthood	reproductive age and older adulthood
						NCD	nutritional status	undernutrition	adulthood	reproductive age and older adulthood
195	Htet, K. K. K., et al. (2021). "Sensitivity and specificity of tuberculosis signs and symptoms screening and adjunct role of social pathology characteristics in predicting bacteriologically confirmed tuberculosis in Myanmar." Trop Med Health 49(1): 3.	291	m-tb200	Myanmar	Natioanl representative	NCD	cvd/circulatory	elevated blood pressure/ hypertension	adulthood	adulthood
409	Ministry of Health, Department of Health, Government of Myanmar, Report on National TB Prevalence Survey 2009-2010, Myanmar					NCD	endocrine and metabolic	elevated glucose/ prediabetes/ diabetes	adulthood	adulthood
						NCD	nutritional status	overweight/obese	adulthood	adulthood
						NCD	nutritional status	undernutrition	adulthood	adulthood
						ID	bacteria	TB	adulthood	adulthood
						ID	virus	hiv	adulthood	adulthood

309	Mean, V., et al. (2018). "Identification and characterization of areas of high and low risk for asymptomatic malaria infections at sub-village level in Ratanakiri, Cambodia." Malaria journal 17(1): 27.	294		Cambodia	Rural	ID	parasite	malaria	lifespan	lifespan
2	Ministry of Health and Sports (MoHS) and ICF. 2017. Myanmar Demographic and Health Survey 2015-16. Nay Pyi Taw, Myanmar, and Rockville, Maryland USA: Ministry of Health and Sports and ICF.	292	mdhs20	Myanmar	Natioanl representative	NCD	blood and blood forming organ	Anemia	adulthood	Reproductive age
49	Anik, A. I., et al. (2019). "Double burden of malnutrition at household level: A comparative study among Bangladesh, Nepal, Pakistan, and Myanmar." PLoS One 14(8): e0221274.					NCD	genitourinary	Menopause	adulthood	reproductive age
92	Blankenship, J. L., et al. (2020). "Childhood stunting and wasting in Myanmar: Key drivers and implications for policies and programmes." Maternal and Child Nutrition 16: e12710.					NCD	nutritional status	overweight/obese	adulthood	reproductive age
139	Das Gupta, R., et al. (2019). "Frequency of television viewing and association with overweight and obesity among women of the reproductive age group in Myanmar: results from a nationwide cross-sectional survey." BMJ Open 9(3): e024680.					NCD	nutritional status	undernutrition	childhood	Pre-school child
185	Hong, S. (2021). "Prevalence and regional variations of coexistence of child stunting and maternal overweight or obesity in Myanmar." Public health nutrition 24(8): 2248-2258.					NCD	nutritional status	undernutrition	adulthood	reproductive age
186	Hong, S. A., et al. (2018). "The prevalence of underweight, overweight and obesity and their related socio-demographic and lifestyle factors among adult women in Myanmar, 2015-16." PLoS One 13(3): e0194454.					ID	non_specific	ARI	childhood	Pre-school child
243	Kang, Y. and J. Kim (2020). "Risk factors for undernutrition among children 0-59 months of age in Myanmar." Proceedings of the Nutrition Society 79(OCE2).					ID	non_specific	STI	adulthood	reproductive age
249	Khaing, H. T., et al. (2019). "Risk factors and regional variations of malnutrition among children under 5 in Myanmar: cross-sectional analyses at national and subnational levels." BMJ Open 9(9): e030894.					ID	parasite	intestine parasite	childhood	Pre-school child
311	Min, K. T., et al. (2020). "Utilization of insecticide-treated bed nets and care-seeking for fever and its associated socio-demographic and geographical factors among under-five children in different regions: evidence from the Myanmar Demographic and Health Survey, 2015-2016." Malar J 19(1): 7.					Symptoms	digestive	diarrhea	childhood	Pre-school child
						Symptoms	multiple/non-specific	fever	childhood	Pre-school child
						Symptoms	respiratory	cough	childhood	Pre-school child
160	George, J., et al. (2012). "Genetic hemoglobin disorders, infection, and deficiencies of iron and vitamin A determine anemia in young Cambodian children." J Nutr 142(4): 781-787.	295		Cambodia	Rural and urban	NCD	blood and blood forming organ	Anemia	childhood	Pre-school child
328	Munkongdee, T., et al. (2016). "Molecular Epidemiology of Hemoglobinopathies in Cambodia." Hemoglobin 40(3): 163-167.					NCD	blood and blood forming organ	Hemoglobinpathy	childhood	Pre-school child
						NCD	nutritional status	micronutrition deficiency	childhood	Pre-school child
						NCD	nutritional status	undernutrition	childhood	Pre-school child
						ID	non_specific	ARI	childhood	Pre-school child
						ID	parasite	intestine parasite	childhood	Pre-school child
						Symptoms	digestive	diarrhea	childhood	Pre-school child
131	Cifuentes, M., et al. (2010). "National income, self-reported wheezing and asthma diagnosis from the World Health Survey." European Respiratory Journal 35(2): 279-286.	293	mwhs	Myanmar	Natioanl representative	NCD	Oral health	teeth	adulthood	adulthood

137	Cristobal-Narvaez, P., et al. (2020). "Perceived stress and depression in 45 low- and middle-income countries." Journal of Affective Disorders 274: 799-805.				NCD	musculoskeleton	Arthritis	adulthood	adulthood
348	Nuevo, R., et al. (2012). "The continuum of psychotic symptoms in the general population: a cross-national study." Schizophr Bull 38(3): 475-485.				NCD	psycological/ mental	Psychosis	adulthood	adulthood
463	Stubbs, B., et al. (2017). "Lifetime self-reported arthritis is associated with elevated levels of mental health burden: A multi-national cross sectional study across 46 low- and middle-income countries." Sci Rep 7(1): 7138.				NCD	psycological/ mental	depression	adulthood	adulthood
499	To, T., et al. (2012). "Global asthma prevalence in adults: findings from the cross-sectional world health survey." BMC Public Health 12: 204.				NCD	respiratory	asthma	adulthood	adulthood
501	Tyrovolas, S., et al. (2016). "Population prevalence of edentulism and its association with depression and self-rated health." Scientific reports 6: 37083.				Symptoms	respiratory	wheezing	adulthood	adulthood
					General health			adulthood	adulthood
					Disability/Lim	Overall	Overall	adulthood	adulthood
					SPH	qol	qol	adulthood	adulthood
					SPH	srh	srh	adulthood	adulthood
489	Teerawichitchainan, B. and J. Knodel (2015). "Economic Status and Old-Age Health in Poverty-Stricken Myanmar." Journal of aging and health 27(8): 1462-1484.	296		Myanmar	Natioanl representative	General health		adulthood	older adulthood and retirement age
495	The situation of older persons in Myanmar (results from the 2012 Survey of Older persons)					Disability/Lim	Activity ADL	Activity ADL	adulthood	older adulthood and retirement age
					Disability/Lim	Function	Function Non-specific	adulthood	older adulthood and retirement age
					Disability/Lim	Function	Function Sensor	adulthood	older adulthood and retirement age
					SPH	srh	srh	adulthood	older adulthood and retirement age
					SPH	srh memory	srh memory	adulthood	older adulthood and retirement age
490	Teerawichitchainan, B. and J. Knodel (2018). "Long-Term Care Needs in the Context of Poverty and Population Aging: the Case of Older Persons in Myanmar." J Cross Cult Gerontol 33(2): 143-162.	297		Myanmar	Natioanl representative	Disability/Lim	Activity ADL	Activity ADL	adulthood	older adulthood and retirement age
					Disability/Lim	Function	Function Non-specific	adulthood	older adulthood and retirement age
396	Rahman, S., et al. (2016). "Determinants of iron status and Hb in the Bangladesh population: the role of groundwater iron." Public Health Nutr 19(10): 1862-1874.	299	national	Bangladesh	Natioanl representative	NCD	blood and blood forming organ	Anemia	adulthood	reproductive age
397	Rahman, S., et al. (2017). "Vitamin A deficiency and determinants of vitamin A status in Bangladeshi children and women: findings of a national survey." Public health nutrition 20(6): 1114-1125.				NCD	nutritional status	micronutrition deficiency	lifespan	lifespan
116	Chher, T., et al. (2016). "Dental Caries Experience in Cambodia: Findings from the 2011 Cambodia National Oral Health Survey." Journal of International Oral Health 8(1): 1-7.	300	national	Cambodia	Rural and urban	NCD	Oral health	teeth	lifespan	lifespan
228	Jenchitr, W., et al. (2011). "Prevalence of age-related macular degeneration in Thailand." Ophthalmic Epidemiol 18(1): 48-52.	301		Thailand	Natioanl representative	NCD	eye	age-related macular degeneration	adulthood	older adulthood and retirement age

38	Ali, N. B., et al. (2019). "Association of food security and other socioeconomic factors with dietary diversity and nutritional statuses of children aged 6-59 months in rural Bangladesh." PLoS One 14(8): e0221929.	298		Bangladesh	Rural and urban	NCD	blood and blood forming organ	Anemia	adulthood	reproductive age
102	Centre for Child and Adolescent Health(CCAH),&International Centre for Diarrhoeal Disease Research Bangladesh(icddr,b), Evaluation of Bangladesh Nutrition Initiative Project/Nobokoli: Findings from Baseline Survey, 2014					NCD	nutritional status	overweight/obese	childhood	Pre-school child
						NCD	nutritional status	undernutrition	childhood	Pre-school child
						ID	non_specific	Pneumonia	childhood	Pre-school child
						Symptoms	digestive	diarrhea	childhood	Pre-school child
						General health			childhood	Pre-school child
365	Pongsachareonnont, P., et al. (2018). "Association between visual status and mental health status in Thai rural elderly: a community-based study." Int J Ophthalmol 11(5): 852-857.	336		Thailand	Not specified	NCD	nutritional status	overweight/obese	adulthood	older adulthood and retirement age
						NCD	nutritional status	undernutrition	adulthood	older adulthood and retirement age
						NCD	psycological/ mental	general	adulthood	older adulthood and retirement age
						Disability/Lim	Activity ADL	Activity ADL	adulthood	older adulthood and retirement age
						Disability/Lim	Function	Function Vision	adulthood	older adulthood and retirement age
215	Islam, J. Y., et al. (2020). "Estimation of total cardiovascular risk using the 2019 WHO CVD prediction charts and comparison of population-level costs based on alternative drug therapy guidelines: a population-based study of adults in Bangladesh." BMJ Open 10(7): e035842.	337		Bangladesh	Natioanl representative	NCD	cvd/circulatory	elevated blood pressure/ hypertension	adulthood	adulthood
						NCD	cvd/circulatory	non-specific	adulthood	adulthood
						NCD	endocrine and metabolic	elevated glucose/ prediabetes/ diabetes	adulthood	adulthood
						NCD	nutritional status	overweight/obese	adulthood	adulthood
262	Khanam, M. A., et al. (2011). "The metabolic syndrome: prevalence, associated factors, and impact on survival among older persons in rural Bangladesh." PLoS One 6(6): e20259.	302		Bangladesh	Rural	NCD	cvd/circulatory	elevated blood pressure/ hypertension	adulthood	older adulthood and retirement age
						NCD	endocrine and metabolic	Metabolic Syndrome	adulthood	older adulthood and retirement age
						NCD	endocrine and metabolic	elevated cholesterol/ hyperlipidemia	adulthood	older adulthood and retirement age
						NCD	endocrine and metabolic	elevated glucose/ prediabetes/ diabetes	adulthood	older adulthood and retirement age
						NCD	nutritional status	overweight/obese	adulthood	older adulthood and retirement age
						NCD	nutritional status	undernutrition	adulthood	older adulthood and retirement age
513	Wasitthankasem, R., et al. (2018). "Birth-cohort HCV screening target in Thailand to expand and optimize the national HCV screening for public health policy." PLoS One 13(8): e0202991.	303		Thailand	Not specified	NCD	psycological/ mental	substance abuse	adulthood	reproductive age
						ID	virus	hcv	adulthood	reproductive age

405	Razzaque, A., et al. (2014). "Do self-reported health indicators predict mortality? Evidence from Matlab, Bangladesh." Journal of biosocial science 46(5): 621-634.	304	sage 20(	Bangladesh	Rural	Disability/Lim	Overall	Overall	adulthood	older adulthood and retirement age
						SPH	qol	qol	adulthood	older adulthood and retirement age
						SPH	srh	srh	adulthood	older adulthood and retirement age
3	A million person household survey: understanding the burden of injuries in Bangladesh	307	saving li	Bangladesh	Rural	NCD	Injury	Animal/insect	adulthood	adulthood
43	Alonge, O., et al. (2017). "Fatal and non-fatal injury outcomes: results from a purposively sampled census of seven rural subdistricts in Bangladesh." Lancet Glob Health 5(8): e818-e827.					NCD	Injury	Blunt object	adulthood	adulthood
54	Ashraf, L., et al. (2019). "Burden of Lesser-Known Unintentional Non-Fatal Injuries in Rural Bangladesh: Findings from a Large-Scale Population-Based Study." International journal of environmental research and public health 16(18).					NCD	Injury	Burn	adulthood	adulthood
79	Bhuiyan, M. A. A., et al. (2019). "Animal-related injuries and fatalities: evidence from a large-scale population-based cross-sectional survey in rural Bangladesh." BMJ Open 9(11): e030039.					NCD	Injury	Cut	adulthood	adulthood
181	He, S., et al. (2017). "Epidemiology of Burns in Rural Bangladesh: An Update." Int J Environ Res Public Health 14(4).					NCD	Injury	Drowning	adulthood	adulthood
188	Hoque, D. M. E., et al. (2017). "Impact of First Aid on Treatment Outcomes for Non-Fatal Injuries in Rural Bangladesh: Findings from an Injury and Demographic Census." Int J Environ Res Public Health 14(7).					NCD	Injury	Electrocution	adulthood	adulthood
381	Rahman, A., et al. (2017). "Epidemiology of Drowning in Bangladesh: An Update." Int J Environ Res Public Health 14(5).					NCD	Injury	Fall	adulthood	adulthood
503	Ul Baset, M. K., et al. (2017). "Pattern of Road Traffic Injuries in Rural Bangladesh: Burden Estimates and Risk Factors." Int J Environ Res Public Health 14(11).					NCD	Injury	Machine	adulthood	adulthood
508	Wadhwaniya, S., et al. (2017). "Epidemiology of fall injury in rural Bangladesh." International journal of environmental research and public health 14(8): 900.					NCD	Injury	Non-specific	adulthood	adulthood
						NCD	Injury	Poinson	adulthood	adulthood
						NCD	Injury	Road traffic	adulthood	adulthood
						NCD	Injury	Suffocation	adulthood	adulthood
						NCD	Injury	Voilence	adulthood	adulthood
						NCD	psycological/ mental	suicidality	lifespan	lifespan
65	BRAC James P Grant School of Public Health, BRAC University and Japan International Cooperation Agency. Baseline Survey of Strengthening Health Systems through Organizing Communities (SHASTO). 2018. Dhaka, Bangladesh. BRAC James P Grant School of Public Health, BRAC University	305	shasto	Bangladesh	Rural and urban	NCD	cvd/circulatory	elevated blood pressure/ hypertension	adulthood	adulthood
178	Hasan, M., et al. (2021). "Prevalence and associated factors of hypertension in selected urban and rural areas of Dhaka, Bangladesh: Findings from SHASTO baseline survey." BMJ Open 11(1): e038975.					NCD	endocrine and metabolic	elevated glucose/ prediabetes/ diabetes	adulthood	adulthood
						NCD	nutritional status	overweight/obese	adulthood	adulthood
						NCD	nutritional status	undernutrition	adulthood	adulthood

487	Tareque, M. I., et al. (2015). "Healthy life expectancy and the correlates of self-rated health in an ageing population in Rajshahi district of Bangladesh." Ageing & Society 35(5): 1075-1094.	308		Bangladesh	Not specified	SPH	srh	srh	adulthood	older adulthood and retirement age
73	Bee Koon, P., et al. (2016). "25-hydroxy-vitamin D demography and the risk of vitamin D insufficiency in the South East Asian Nutrition Surveys (SEANUTS)." Asia Pacific Journal of Clinical Nutrition 25(3): 538-548.	309	south ea	Thailand	Natioanl representative	NCD	blood and blood forming organ	Anemia	childhood	Child
416	Rojroongwasinkul, N., et al. (2013). "SEANUTS: the nutritional status and dietary intakes of 0.5-12-year-old Thai children." Br J Nutr 110 Suppl 3: S36-44.					NCD	nutritional status	micronutrition deficiency	childhood	Child
						NCD	nutritional status	overweight/obese	childhood	Child
						NCD	nutritional status	undernutrition	childhood	Child
170	Hanif, A. A. M., et al. (2021). "Gender-specific prevalence and associated factors of hypertension among elderly Bangladeshi people: Findings from a nationally representative cross-sectional survey." BMJ Open 11(1): e038326.	310	state of	Bangladesh	Natioanl representative	NCD	cvd/circulatory	elevated blood pressure/ hypertension	adulthood	adulthood
171	Hanif, A. A. M., et al. (2021). "Ten-years cardiovascular risk among Bangladeshi population using non-laboratory-based risk chart of the World Health Organization: Findings from a nationally representative survey." PLoS One 16(5): e0251967.					NCD	cvd/circulatory	heart disease	adulthood	older adulthood and retirement age
461	State of Food security and nutrition in Bangladesh 2018- 2019					NCD	endocrine and metabolic	elevated glucose/ prediabetes/ diabetes	adulthood	adulthood
						NCD	genitourinary	kidney diesease	adulthood	older adulthood and retirement age
						NCD	nutritional status	micronutrition deficiency	adulthood	reproductive age and older adulthood
						NCD	nutritional status	overweight/obese	adulthood	adulthood
						NCD	nutritional status	undernutrition	childhood	Pre-school child
						NCD	nutritional status	undernutrition	adulthood	older adulthood and retirement age
						NCD	psycological/ mental	general	adulthood	older adulthood and retirement age
						NCD	respiratory	asthma	adulthood	older adulthood and retirement age
173	Haque, M. A., et al. (2021). "Determinants of maternal low mid-upper arm circumference and its association with child nutritional status among poor and very poor households in rural Bangladesh." Maternal & Child Nutrition 17(4): 1-12.	306	suchana	Bangladesh	Rural	NCD	nutritional status	overweight/obese	childhood	Pre-school child
465	Choudhury, N., Raihan, M.J., Ahmed, S.M.T. et al. The evaluation of Suchana, a large-scale development program to prevent chronic undernutrition in north-eastern Bangladesh. BMC Public Health 20, 744 (2020). https://doi.org/10.1186/s12889-020-08769-4					NCD	nutritional status	undernutrition	childhood	Pre-school child
						NCD	nutritional status	undernutrition	adulthood	reproductive age
48	Andrews, J. R., et al. (2020). "Healthcare Utilization Patterns for Acute Febrile Illness in Bangladesh, Nepal, and Pakistan: Results from the Surveillance for Enteric Fever in Asia Project." Clin Infect Dis 71(Suppl 3): S248-S256.	311	surveilla	Bangladesh	Urban	Symptoms	multiple/non-specific	fever	childhood	Child

8	Aekplakorn, W. (2011). "Prevalence, treatment, and control of metabolic risk factors by BMI status in Thai adults: National Health Examination Survey III." Asia Pac J Public Health 23(3): 298-306.	312	t-nhes ii	Thailand	Natioanl representative	NCD	cvd/circulatory	elevated blood pressure/ hypertension	adulthood	adulthood
13	Aekplakorn, W., et al. (2015). "Association of serum bisphenol a with hypertension in thai population." Int J Hypertens 2015: 594189.					NCD	endocrine and metabolic	elevated cholesterol/ hyperlipidemia	adulthood	adulthood
267	Khonputsa, P., et al. (2012). "Joint prevalence and control of hypercholesterolemia and hypertension in Thailand: third national health examination survey." Asia Pac J Public Health 24(1): 185-194.					NCD	endocrine and metabolic	elevated glucose/ prediabetes/ diabetes	adulthood	adulthood
						NCD	nutritional status	overweight/obese	adulthood	adulthood
						NCD	nutritional status	undernutrition	adulthood	adulthood
9	Aekplakorn, W., et al. (2011). "Prevalence and management of diabetes and metabolic risk factors in Thai adults: the Thai National Health Examination Survey IV, 2009." Diabetes Care 34(9): 1980-1985.	313	t-nhes iv	Thailand	Natioanl representative	NCD	Injury	Fall	adulthood	older adulthood and retirement age
11	Aekplakorn, W., et al. (2011). "Urban and rural variation in clustering of metabolic syndrome components in the Thai population: results from the fourth National Health Examination Survey 2009." BMC Public Health 11: 854.					NCD	Injury	Non-specific	adulthood	adulthood
12	Aekplakorn, W., et al. (2012). "Changes in prevalence, awareness, treatment and control of hypertension in Thai population, 2004-2009: Thai National Health Examination Survey III-IV." J Hypertens 30(9): 1734-1742.					NCD	blood and blood forming organ	Anemia	adulthood	adulthood
13	Aekplakorn, W., et al. (2015). "Association of serum bisphenol a with hypertension in thai population." Int J Hypertens 2015: 594189.					NCD	cvd/circulatory	Stroke	adulthood	adulthood
14	Aekplakorn, W., et al. (2015). "Relationship of serum bisphenol A with diabetes in the Thai population, National Health Examination Survey IV, 2009." Journal of diabetes 7(2): 240-249.					NCD	cvd/circulatory	elevated blood pressure/ hypertension	adulthood	adulthood
52	Apinonkul, B., et al. (2015). "Gender differences in health expectancies across the disablement process among older Thais." PLoS One 10(3): e0121310.					NCD	endocrine and metabolic	Metabolic Syndrome	adulthood	adulthood
104	Chailurkit, L. O., et al. (2011). "Regional variation and determinants of vitamin D status in sunshine-abundant Thailand." BMC Public Health 11: 853.					NCD	endocrine and metabolic	elevated cholesterol/ hyperlipidemia	adulthood	adulthood
106	Chailurkit, L. O., et al. (2016). "The Association of Serum Bisphenol A with Thyroid Autoimmunity." Int J Environ Res Public Health 13(11).					NCD	endocrine and metabolic	elevated glucose/ prediabetes/ diabetes	adulthood	adulthood
107	Chailurkit, L.-O., et al. (2012). "The association between vitamin D status and type 2 diabetes in a Thai population, a cross-sectional study." Clinical Endocrinology 77(5): 658-664.					NCD	endocrine and metabolic	positive for thyroid autoantibodies	adulthood	adulthood
108	Chailurkit, L.-o., et al. (2013). "High vitamin D status in younger individuals is associated with low circulating thyrotropin." Thyroid : official journal of the American Thyroid Association 23(1): 25-30.					NCD	endocrine and metabolic	positive serum thyrotropin, tpoab, tgab	adulthood	adulthood
294	Lertrit, A., et al. (2019). "Thyroid function is associated with body mass index and fasting plasma glucose in Thai euthyroid population." Diabetes Metab Syndr 13(1): 468-473.					NCD	nutritional status	micronutrition deficiency	adulthood	adulthood
343	Nguyen Ngoc, H., et al. (2019). "Association of Adiposity Indices with Hypertension in Middle-Aged and Elderly Thai Population: National Health Examination Survey 2009 (NHES-IV)." J Cardiovasc Dev Dis 6(1).					NCD	nutritional status	overweight/obese	adulthood	adulthood
456	Srinonprasert, V., et al. "Vitamin D insufficiency predicts mortality among older men, but not women: A nationwide retrospective cohort from Thailand." Geriatr Gerontol Int 18(12): 1585-1590.					NCD	nutritional status	undernutrition	adulthood	adulthood

457	Sriphrapradang, C., et al. (2014). "Reference ranges of serum TSH, FT4 and thyroid autoantibodies in the Thai population: the national health examination survey." Clin Endocrinol (Oxf) 80(5): 751-756.					NCD	psycological/ mental	dementia	adulthood	older adulthood and retirement age
517	Wichai Ekplakorn, (Ed.), The report of the Thai people's health survey by physical examination the 4th time, 2008					NCD	psycological/ mental	depression	adulthood	adulthood
520	Wichaidit, W., et al. (2014). "Religious affiliation and disparities in risk of non-communicable diseases and health behaviours: findings from the fourth Thai National Health Examination Survey." Glob Public Health 9(4): 426-435.					General health			adulthood	older adulthood and retirement age
						Disability/Lim	Activity ADL	Activity ADL	adulthood	older adulthood and retirement age
						Disability/Lim	Body structure&function	Body structure&function	adulthood	older adulthood and retirement age
						Disability/Lim	Function	Function Cognition	adulthood	older adulthood and retirement age
15	Aekplakorn, W., et al. (2018). "Prevalence of Diabetes and Relationship with Socioeconomic Status in the Thai Population: National Health Examination Survey, 2004-2014." J Diabetes Res 2018: 1654530.	314	t-nhes v	Thailand	Natioanl representative	NCD	Injury	Fall	adulthood	older adulthood and retirement age
16	Aekplakorn, W., et al. (2021). "Women and other risk factors for chronic kidney disease of unknown etiology in Thailand: National Health Examination V Survey." Scientific reports 11(1): 21366.					NCD	Injury	Non-specific	adulthood	adulthood
56	Assanangkornchai, S., et al. (2020). "Socio-economic inequalities in the association between alcohol use disorder and depressive disorder among Thai adults: a population-based study." BMC psychiatry 20(1): 553.					NCD	blood and blood forming organ	Anemia	adulthood	adulthood
271	Kitiyakara, C. and W. Aekplakorn (2021). "Prevalence and regional distribution of CKD and CKD of unknown aetiology in Thailand: National health examination v survey." Nephrology 26(SUPPL 1): 8.					NCD	cvd/circulatory	elevated blood pressure/ hypertension	adulthood	adulthood
374	Prasitsiriphon, O. and D. Weber (2019). "Objective physical measures and their association with subjective functional limitations in a representative study population of older Thais." BMC geriatrics 19(1): 73.					NCD	endocrine and metabolic	Metabolic Syndrome	childhood	School-age child
447	Siwarom, S., et al. (2021). "Metabolic syndrome in Thai adolescents and associated factors: the Thai National Health Examination Survey V (NHES V)." BMC Public Health 21(1): 678.					NCD	endocrine and metabolic	elevated cholesterol/ hyperlipidemia	adulthood	adulthood
448	Siwarom, S., et al. (2022). "Waist-to-Height Ratio Is a Good Predictor of Metabolic Syndrome in Adolescents: A Report From the Thai National Health Examination Survey V, 2014." Asia Pac J Public Health 34(1): 36-43.					NCD	endocrine and metabolic	elevated glucose/ prediabetes/ diabetes	adulthood	adulthood
511	Washirasaksiri, C., et al. (2021). "Increasing glycaemia is associated with a significant decline in HDL cholesterol in women with prediabetes in two national populations." Scientific reports 11(1): 12194.					NCD	genitourinary	CKD	adulthood	adulthood
518	Wichai Ekplakorn, (Ed.), The report of the Thai people's health survey by physical examination the 5th time, 2014					NCD	nutritional status	overweight/obese	adulthood	adulthood
						NCD	nutritional status	undernutrition	adulthood	adulthood
						NCD	psycological/ mental	dementia	adulthood	older adulthood and retirement age
						NCD	psycological/ mental	depression	adulthood	adulthood
						NCD	psycological/ mental	substance abuse	adulthood	adulthood
						Symptoms	genitourinary	proteinuria	adulthood	adulthood
						General health			adulthood	adulthood

						Disability/Lim	Activity ADL	Activity ADL	adulthood	older adulthood and retirement age
						Disability/Lim	Body structure&function	Body structure&function	adulthood	older adulthood and retirement age
						Disability/Lim	Function	Function Cognition	adulthood	older adulthood and retirement age
519	Wichai Ekplakorn, (Ed.), The report of the Thai people's health survey by physical examination the 6th time, 2019-2020	315	t-nhes v	Thailand	Natioanl representative	NCD	Injury	Fall	adulthood	older adulthood and retirement age
						NCD	Injury	Non-specific	adulthood	adulthood
						NCD	blood and blood forming organ	Anemia	adulthood	adulthood
						NCD	cvd/circulatory	elevated blood pressure/ hypertension	adulthood	adulthood
						NCD	endocrine and metabolic	elevated cholesterol/ hyperlipidemia	adulthood	adulthood
						NCD	endocrine and metabolic	elevated glucose/ prediabetes/ diabetes	adulthood	adulthood
						NCD	nutritional status	overweight/obese	adulthood	adulthood
						NCD	psycological/ mental	depression	adulthood	adulthood
						General health			adulthood	adulthood
						Disability/Lim	Activity ADL	Activity ADL	adulthood	older adulthood and retirement age
						Disability/Lim	Body structure&function	Body structure&function	adulthood	older adulthood and retirement age
168	Hanchaiphiboolkul, S., et al. (2011). "Prevalence of stroke and stroke risk factors in thailand: Thai epidemiologic stroke (TES) study." Journal of the Medical Association of Thailand 94(4): 427-436.	317	thai epic	Thailand	Rural and urban	NCD	cvd/circulatory	Stroke	adulthood	adulthood
169	Hanchaiphiboolkul, S., et al. (2013). "Risk of metabolic syndrome for stroke is not greater than the sum of its components: Thai Epidemiologic Stroke (TES) study." Journal of stroke and cerebrovascular diseases : the official journal of National Stroke Association 22(8): e264-270.					NCD	endocrine and metabolic	Metabolic Syndrome	adulthood	adulthood
234	Jitnarin, N., et al. (2010). "Risk factors for overweight and obesity among Thai adults: Results of the national Thai food consumption survey." Nutrients 2(1): 60-74.	318	thai food	Thailand	Natioanl representative	NCD	nutritional status	overweight/obese	lifespan	lifespan
235	Jitnarin, N., et al. (2011). "Prevalence of overweight and obesity in Thai population: results of the National Thai Food Consumption Survey." Eat Weight Disord 16(4): e242-249.					NCD	nutritional status	overweight/obese	adulthood	adulthood
						NCD	nutritional status	undernutrition	adulthood	adulthood
459	Srisurapanont, M., et al. (2012). "Patterns of alcohol dependence in Thai drinkers: a differential item functioning analysis of gender and age bias." Addict Behav 37(2): 173-178.	323	thai nati	Thailand	Natioanl representative	NCD	psycological/ mental	anxiety	adulthood	reproductive age and older adulthood
473	Suttajit, S., et al. (2012). "Risks of major depressive disorder and anxiety disorders among Thais with alcohol use disorders and illicit drug use: findings from the 2008 Thai National Mental Health survey." Addictive behaviors 37(12): 1395-1399.					NCD	psycological/ mental	depression	adulthood	reproductive age and older adulthood

						NCD	psycological/ mental	substance abuse	adulthood	reproductive age and older adulthood
55	Assanangkornchai, S., et al. (2016). "Gambling disorders, gambling type preferences, and psychiatric comorbidity among the Thai general population: Results of the 2013 national mental health survey." Journal of Behavioral Addictions 5(3): 410-418.	319	thai nati	Thailand	Natioanl representative	NCD	psycological/ mental	Psychosis	adulthood	adulthood
471	Supanya, S., et al. (2017). "Prevalence of psychotic experiences and traumatic events in the 2013 Thai National mental health survey." Schizophrenia Bulletin 43(Supplement 1): S183.					NCD	psycological/ mental	agoraphobia	adulthood	adulthood
472	Supanya, S., et al. (2017). "Psychotic experiences in Thailand: Results from the 2013 Thai National Mental Health Survey." European Archives of Psychiatry and Clinical Neuroscience 267(1 Supplement 1): S61.					NCD	psycological/ mental	anxiety	adulthood	adulthood
						NCD	psycological/ mental	depression	adulthood	adulthood
						NCD	psycological/ mental	intermittent explosive disorder	adulthood	adulthood
						NCD	psycological/ mental	pathological gambling, problem gambling	adulthood	adulthood
						NCD	psycological/ mental	ptsd	adulthood	adulthood
						NCD	psycological/ mental	suicidality	adulthood	adulthood
444	Singsalasang, A., et al. (2017). "Socioeconomic disparities in income, education and geographic location for hypertension among Thai adults: Results from the National Socioeconomic Survey." F1000Res 6: 1836.	321	thai nati	Thailand	Natioanl representative	NCD	cvd/circulatory	elevated blood pressure/ hypertension	adulthood	adulthood
302	Luenam, A., et al. (2018). "Socioeconomic disparities and chronic respiratory diseases in Thailand: The National Socioeconomics Survey." Inform Health Soc Care 43(4): 348-361.	320	thai nati	Thailand	Natioanl representative	General health			lifespan	lifespan
179	Haseen, F., et al. (2010). "Self-assessed health among Thai elderly." BMC geriatrics 10: 30.	322	thai nati	Thailand	Natioanl representative	NCD	Oral health	teeth	adulthood	older adulthood and retirement age
458	Srisilapanan, P., et al. (2016). "Associations between social inequality and tooth loss in a household sample of elderly Thai people aged >/=60 years old." Gerodontology 33(2): 201-208.					NCD	cvd/circulatory	elevated blood pressure/ hypertension	adulthood	older adulthood and retirement age
						NCD	endocrine and metabolic	elevated glucose/ prediabetes/ diabetes	adulthood	older adulthood and retirement age
						NCD	neoplasma	cancer	adulthood	older adulthood and retirement age
						NCD	neurology	paralysis	adulthood	older adulthood and retirement age
						NCD	psycological/ mental	general	adulthood	older adulthood and retirement age
						Disability/Lim	Function	Function Non-specific	adulthood	older adulthood and retirement age
						SPH	srh	srh	adulthood	older adulthood and retirement age
494	The situation of Thailand's older population: An update based on the 2014 Survey of Older Persons in Thailand	324	thai olde	Thailand	Rural and urban	Disability/Lim	Activity ADL	Activity ADL	adulthood	older adulthood and retirement age
						SPH	srh	srh	adulthood	older adulthood and retirement age

201	Ingsathit, A., et al. (2010). "Prevalence and risk factors of chronic kidney disease in the Thai adult population: Thai SEEK study." Nephrol Dial Transplant 25(5): 1567-1575.	325	thai seel	Thailand	Rural and urban	NCD	blood and blood forming organ	Anemia	adulthood	adulthood
492	Thakkinstian, A., et al. (2011). "A simplified clinical prediction score of chronic kidney disease: a cross-sectional-survey study." BMC Nephrol 12: 45.					NCD	cvd/circulatory	Stroke	adulthood	adulthood
						NCD	cvd/circulatory	elevated blood pressure/ hypertension	adulthood	adulthood
						NCD	cvd/circulatory	heart disease	adulthood	adulthood
						NCD	endocrine and metabolic	elevated cholesterol/ hyperlipidemia	adulthood	adulthood
						NCD	endocrine and metabolic	elevated glucose/ prediabetes/ diabetes	adulthood	adulthood
						NCD	genitourinary	CKD	adulthood	adulthood
						NCD	genitourinary	kidney stone	adulthood	adulthood
279	Krisdapong, S. and A. Sheiham (2014). "Which aspects of an oral health-related quality of life measure are mainly associated with global ratings of oral health in children?" Community Dent Oral Epidemiol 42(2): 129-138.	328	thailand	Thailand	Natioanl representative	NCD	Oral health	overall	childhood	School-age child
280	Krisdapong, S., et al. (2012). "Impacts of recurrent aphthous stomatitis on quality of life of 12- and 15-year-old Thai children." Qual Life Res 21(1): 71-76.					Disability/Lim	Activity ADL	Activity ADL	childhood	School-age child
281	Krisdapong, S., et al. (2012). "Relationships between oral diseases and impacts on Thai schoolchildren's quality of life: Evidence from a Thai national oral health survey of 12- and 15-year-olds." Community Dentistry & Oral Epidemiology 40(6): 550-559.					Disability/Lim	Activity	Activity Oral-impact	childhood	School-age child
282	Krisdapong, S., et al. (2012). "Setting oral health goals that include oral health-related quality of life measures: a study carried out among adolescents in Thailand." Cadernos de saude publica 28(10): 1881-1892.					SPH	qol oral	qol oral	childhood	School-age child
283	Krisdapong, S., et al. (2012). "The impacts of gingivitis and calculus on Thai children's quality of life." J Clin Periodontol 39(9): 834-843.					SPH	srh oral	srh oral	childhood	School-age child
284	Krisdapong, S., et al. (2012). "Using associations between oral diseases and oral health-related quality of life in a nationally representative sample to propose oral health goals for 12-year-old children in Thailand." Int Dent J 62(6): 320-330.									
285	Krisdapong, S., et al. (2013). "Impacts on quality of life related to dental caries in a national representative sample of thai 12-and 15-year-olds." Caries research 47(1): 9-17.									
286	Krisdapong, S., et al. (2013). "Sociodemographic differences in oral health-related quality of life related to dental caries in thai school children." Community Dent Health 30(2): 112-118.									
287	Krisdapong, S., et al. (2014). "Associations between perceived needs for dental treatment, oral health-related quality of life and oral diseases in school-aged Thai children." Community Dentistry & Oral Epidemiology 42(4): 323-332.									
44	Amornsuradech, S. and W. Vejvithee (2019). "Socioeconomic inequality and dental caries among Thai working age population Analysis of Thailand National Oral Health Survey." Journal of Health Research 33(6): 517-528.	326	thailand	Thailand	Natioanl representative	NCD	Oral health	oral cavity	childhood	School-age child

373	Prasertsom, P., et al. (2020). "Condition-Specific Oral Health Impacts in Thai Children and Adolescents: Findings From the National Oral Health-Related Quality of Life Survey." Asia Pac J Public Health 32(1): 49-56.					NCD	endocrine and metabolic	elevated glucose/ prediabetes/ diabetes	adulthood	reproductive age
						NCD	nutritional status	overweight/obese	adulthood	reproductive age
						NCD	nutritional status	undernutrition	adulthood	reproductive age
						Disability/Lim	Activity	Activity Oral-impact	childhood	School-age child
351	Oshiro, A., et al. (2021). "Inequalities in periodontal disease according to insurance schemes in Thailand." International journal of environmental research and public health 18(11): 5945.	327	thailand	Thailand	Natioanl representative	NCD	Oral health	periodontal	adulthood	reproductive age
350	Okubo, T., et al. (2020). "Risk factors modifying the double burden of malnutrition of young children in Thailand." Maternal & Child Nutrition 16: 1-9.	316	tmics20	Thailand	Natioanl representative	NCD	child development	low birth weight	childhood	Pre-school child
441	Shinsugi, C. and A. Mizumoto (2021). "Associations of Nutritional Status with Full Immunization Coverage and Safe Hygiene Practices among Thai Children Aged 12-59 Months." Nutrients 14(1).					NCD	nutritional status	overweight/obese	childhood	Pre-school child
479	TMICS2015 (Thailand MICS)					NCD	nutritional status	undernutrition	childhood	Pre-school child
						NCD	psycological/ mental	aggressive behavior	childhood	Child
						ID	non_specific	ARI	childhood	Pre-school child
						Symptoms	digestive	diarrhea	childhood	Pre-school child
						Symptoms	multiple/non-specific	fever	childhood	Pre-school child
398	Rakhshanda, S., et al. (2021). "Malnutrition in all its forms and associated factors affecting the nutritional status of adult rural population in Bangladesh: results from a cross-sectional survey." BMJ Open 11(10): e051701.	329	who pac	Bangladesh	Rural	NCD	nutritional status	overweight/obese	adulthood	adulthood
						NCD	nutritional status	undernutrition	adulthood	adulthood
486	Tareque, M. I., et al. (2015). "Healthy life expectancy and the correlates of self-rated health in Bangladesh in 1996 and 2002." BMC Public Health 15: 312.	330	world va	Bangladesh	Natioanl representative	SPH	srh	srh	adulthood	adulthood
